# Meeting abstracts from the 10th International Conference on cGMP: Generators, Effectors and Therapeutic Implications

**DOI:** 10.1186/s12967-022-03800-1

**Published:** 2023-01-31

## ORAL PRESENTATIONS

## Session 1 | Pre-Clinical Translation & Back-Translation

## O1 Applying translational approaches for the nonclinical and clinical evaluation of the sGC stimulator CY6463 in CNS diseases

### Christopher J. Winrow

#### Cyclerion Therapeutics, Cambridge Massachusetts, USA

##### **Correspondence:** Christopher J. Winrow (cwinrow@cyclerion.com)

*J Transl Med* 2022, **21(1)**:O1

**Introduction:** The NO-sGC-cGMP pathway plays a critical role in central nervous system (CNS) function and is impacted across a range of neurological and psychiatric diseases. NO is recognized as a key neurotransmitter that is produced on-demand within the CNS and can act through sGC and cGMP to govern a range of downstream effects. We have identified CY6463, a CNS-penetrant sGC stimulator, with demonstrated pharmacological effects in nonclinical and clinical studies. By acting as a selective positive allosteric modulator of sGC, CY6463 can amplify endogenous NO signaling while maintaining upstream spatial and temporal regulation. This enables the on-demand production of cGMP and propagation of downstream signals within the CNS.

**Methods:** A range of nonclinical studies were conducted to understand the in vitro and in vivo properties of CY6463 and supported advancement into clinical development. Phase 1 clinical studies included single-ascending dose, multiple-ascending dose and food interaction studies along with a translational pharmacology study in healthy elderly participants.

**Results:** This presentation will describe the nonclinical pharmacology of CY6463, along with clinical data from Phase 1 studies including the pharmacokinetic, safety, and pharmacodynamic results of our clinical translational pharmacology study in elderly participants. Furthermore, we will discuss our translational biomarker strategy that has been carried through into clinical studies in three separate patient populations and provide outlines of these clinical studies and updates on progress to date.

**Conclusions:** Applying a translational biomarker based approach to the development of CY6463 has enabled advancement of clinical studies in well-defined patient populations to help understand the potential opportunity for modulating sGC function in neuropsychiatric and neurodegenerative diseases.

**Acknowledgements:** CJW is an employee of Cyclerion Therapeutics and gratefully acknowledges the contributions of the Cyclerion team members and collaborators to this project.

## O2 sGC modulators as cognitive enhancers: neuronal and/or vascular?

### Jos Prickaerts^1^, Ellis Nelissen^1^, Tim Vanmierlo^1,2^, Peter Sandner^3,4^

#### ^1^Maastricht University, Psychiatry and Neuropsychology, Maastricht, Netherlands; Hasselt University, Biomedical Research Institute, Hasselt, Belgium; ^3^Bayer AG, Cardiovascular Research, Wuppertal, Germany; ^4^Hannover Medical School, Hannover, Germany

##### **Correspondence:** Jos Prickaerts (jos.prickaerts@maastrichtuniversity.nl)

*J Transl Med* 2022, **21(1)**:O2

**Introduction:** Cognitive impairment is one of the main symptoms of Alzheimer’s disease or Vascular dementia, which negatively impacts the quality of life of patients. Therefore, a pharmacological intervention that has memory enhancing effects would be beneficial to patients. Vascular dementia is characterized by impairments in cerebral blood flow, endothelial function and blood–brain barrier integrity. These processes are all physiologically regulated by the soluble guanylate cyclase (sGC)-cGMP signaling pathway in blood vessel cells. Additionally, neuronal cGMP signaling plays an important role in long-term potentiation underlying memory formation. Therefore, targeting the NO-sGC-cGMP pathway may be a therapeutic strategy for treating neuronal- and/or vascular-based dementias.

**Methods:** sGC stimulators acting on heme‐bound sGC and one sGC activator acting on heme‐free sGC were tested in the object location task (OLT) on acquisition memory processes, in healthy rodents and in deficit models. Vascular function and neuroplasticity were assessed.

**Results:** The non-brain penetrant sGC stimulators riociguat and vericiguat improved memory acquisition in the OLT in rodents. Riociguat attenuated memory deficits in a cerebral vasoconstriction model for memory impairment induced by sumatriptan. The effective doses of vericiguat had no effect on mean arterial blood pressure and cerebral blood volume. This suggests that non-brain penetrant sGC stimulators improve memory via an effect on the cerebral microvasculature. The brain penetrant sGC stimulator BAY-747 and activator runcaciguat both improved memory acquisition in the OLT in rats. Interestingly only BAY‐747 reversed the NOS inhibitor L‐NAME induced memory impairment. Both BAY‐747 and runcaciguat increased mBDNF levels in the hippocampus. Both drugs also enhanced GluA1‐containing AMPA receptors trafficking in a chemical LTP model for memory acquisition using mouse hippocampal slices. Yet, only for runcaciguat this involved phosphorylation of the receptor on S845.

**Conclusions:** Both sGC stimulators and activators have potential as cognitive enhancers. sGC stimulators have effects on microvasculature as well as neuroplasticity. Effects on neuroplasticity are also exerted by sGC activators, yet with different underlying mechanisms than sGC stimulators. Further elucidating these properties will help in determining which type of sGC modulator can optimally improve cognition in a vascular and/or neuronal type of dementia.

**Funding:** Funding was in part supported by a restricted grant from Bayer AG and Merck Sharp & Dohme Corp.

## O3 Discovery and preclinical profiling of the oral sGC activator runcaciguat (BAY 1101042): a novel and effective treatment approach for chronic kidney disease?

### Peter Sandner^1,2^

#### ^1^Bayer AG, Research and Early Development, Wuppertal, Germany; ^2^Hannover Medical School, Department of Pharmacology, Hannover, Germany

##### **Correspondence:** Peter Sandner (peter.sandner@bayer.com)

*J Transl Med* 2022, **21(1)**:O3

*Peter Sandner is presenting on behalf of the entire research and development teams working on runcaciguat (BAY 1101042) in recent years, especially Michael G. Hahn [1], Thomas Lampe [1], Sherif El Sheikh 1], Niels Griebenow [1], Elisabeth Woltering [1], Karl-Heinz Schlemmer [1], Lisa Dietz [1], Michael Gerisch [1], Frank Wunder [1], Eva Maria Becker-Pelster [1], Thomas Mondritzki [1,2], Hanna Tinel [1], Andreas Knorr [1], Achim Kern [1], Dieter Lang [1],Tibor Schomber [1], Agnes Benardeau [2], Antje Kahnert [1], Laura Popp [1], Julia Vienenkoetter [1], Heidrun Ellinger-Ziegelbauer [1], Mira Pavkovic [1], Axel Kretschmer [1], Bettina Lawrenz [1], Elke Hartmann [1], Krystyna Siudak [1], Alexius Freyberger [1], Ina Hagelschuer [1], Jutta Meyer [1], Jan R. Kraehling [1], Ilka Mathar [1], Joachim Mittendorf [1], Hubert Truebel [2,4], Joerg Hueser [1], Volker Geiss [1], Frank Eitner [1,5], and Johannes-Peter Stasch [1,6];*

- ^*1*^ *Bayer AG* *,* *Research and Early Development, Pharma Research Center, 42096 Wuppertal, Germany,*
- ^*2*^ *University of Witten/Herdecke, 58455 Witten, Germany,*
- ^*3*^ *Current employer: Novo Nordisk A/S* *,* *Cardio-Renal Biology, Måløv, Denmark Novo Nordisk,*
- ^*4*^ *Current employer: AiCuris AG* *,* *Wuppertal, Germany,*
- ^*5*^ *Division of Nephrology and Clinical Immunology, RWTH Aachen University, 52062 Aachen, Germany,*
- ^*6*^ *Institute of Pharmacy, University Halle-Wittenberg, 06120 Halle, Germany,*
- ^*7*^ *Department of Pharmacology, Hannover Medical School, 30625 Hannover, Germany.*

**Introduction:** The class of sGC activators has an unique mode of action by activating the oxidized and heme-free form of sGC which is not responsive to nitric oxide (NO). The sGC activators can restore cGMP signaling under oxidative stress conditions, which is present in a variety of diseases and which could potentially result in a broad therapeutic profile of the sGC activators. However, the first generation of sGC activators exhibits limitations and was discontinued. With the discovery of the novel, oral sGC activator runcaciguat (BAY 1101042), the second generation of sGC activators is available with improved solubility, permeability, metabolism, and drug-drug interactions parameters. The discovery and preclinical profiling of runcaciguat in vitro, ex vivo, and in vivo, in both mechanistic and disease models, will be presented.

**Methods:** Runcaciguat was broadly profiled in vitro, ex vivo, and in vivo. Runcaciguat was tested in mechanistic in vitro, and ex vivo assays and mechanistically studied in vivo models. Moreover, runcaciguat, was tested in different disease models, especially in models for CKD with different etiologies and comorbidities.

**Results:** Runcaciguat exhibits the profile of a potent and selective sGC activator, which independently of NO stimulates cGMP production in vitro, on the isolated sGC enzyme as in cellular tests. Ex vivo, runcaciguat can relax vascular tissues in different vascular beds. In vivo, runcaciguat leads to a dose-dependent decrease in blood pressure. In different rat CKD models, in renin transgenic (RenTG) and angiotensin-supplemented (ANG-SD) rats, but also in rats with diabetic and metabolic CKD, e.g. the Zucker diabetic fatty (ZDF) rat and ZSF-1 rat, runcaciguat significantly reduced proteinuria. In addition, biomarkers and histopathological markers of kidney damage were also significantly reduced. These kidney-protective effects were also significant at doses that did not or only moderately decrease systemic blood pressure.

**Conclusions:** In summary, these data demonstrate that runcaciguat (BAY 1101042) exhibits a typical profile of an oral sGC activator in vitro, ex vivo, and in vivo. Moreover, runcaciguat treatment leads to significant renal protection at doses that do not reduce blood pressure and is effective in hypertensive as well as diabetic and metabolic CKD models. Therefore, the sGC activator runcaciguat could represent an efficient treatment approach for CKD (Figure 1). Runcaciguat is currently developed in phase 2 in patients with chronic kidney disease (CONCORD trial, NCT04507061).
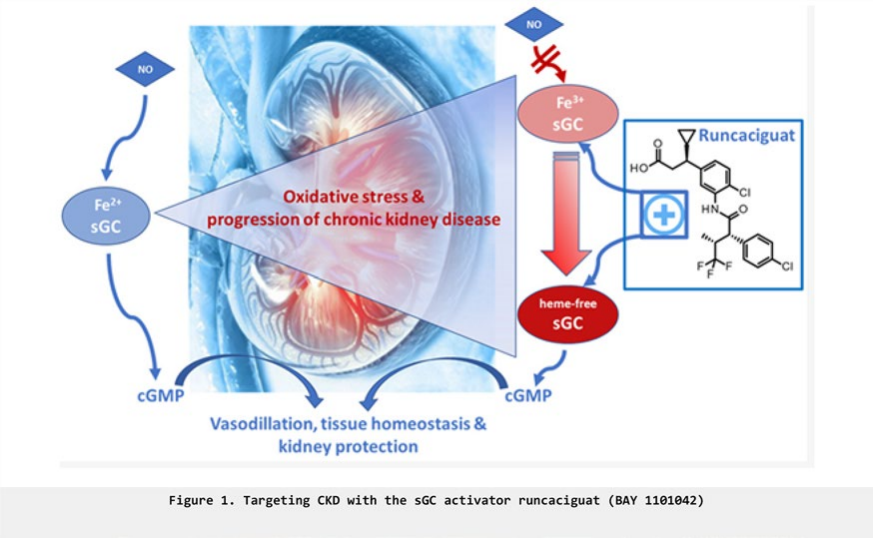


## O4 From phenotype to endotype: preclinical validation of ROS-cGMPopathies in the Estonian Biobank

### Cristian Nogales^1^, Jaanika Kronberg^2^, Alexandra Petraina^1^, Mayra Pacheco Pachado^1,3^, Jerremy Weerts^4^, Arantxa Barandiaran Aizpurua^4^, Vanessa van Empel^4^, Mari Nelis^2^, Lili Milani^2^, Tōnu Esko^2^, Andres Metspalu^2^, Reedik Mägi^2^, Harald H. H. W. Schmidt^1^

#### ^1^Maastricht University, Department of Pharmacology & Personalised Medicine, Maastricht, Netherlands; ^2^University of Tartu, Institute of Genomics, Tartu, Estonia; ^3^Maastricht University, Department of Psychiatry and Neuropsychology, Maastricht, Netherlands; ^4^Department of Cardiology, Cardiovascular Research Institute Maastricht (CARIM), Maastricht University Medical Centre + (MUMC +), Maastricht, The Netherlands

##### **Correspondence:** Cristian Nogales (cnogales@ppmlab.net)

*J Transl Med* 2022, **21(1)**:O4

**Introduction:** Most disease definitions are organ- or symptom- based and rarely reflect the causal mechanism of the disease, meaning we hardly understand any disease mechanistically and low precision drug interventions are the norm in the clinics [1, 2]. Naming a disease after a symptom also runs the risk that different molecular mechanisms that cause a similar symptom are subsumed under one umbrella term. They are thus converted into one common and complex disease entity, which is impossible to untangle based on the current diagnostic tools. Recent efforts have been made to redefine diseases by moving from symptom and organ to mechanism and cause [3]. The ultimate aim is to endotype patients and reach precision medicine. cGMPopathies is a newly described term, which describes a dysfunctional cGMP signalling by (i) reduced synthesis, (ii) increased breakdown or (iii) defective downstream signalling, of cGMP [4]. We have previously identified a disease mechanism, involving genes related to reactive oxygen species (ROS) formation and cGMP signalling (ROCG) i.e., ROS-cGMPopathies. This disease mechanism or disease module is causal for a heterogeneous diseasome cluster of metabolic-cerebro-cardiovascular disease phenotypes [5].

**Methods:** In collaboration with the Estonian biobank, we use a high-throughput screening plasma antibody-based assay to analyze over 1000 human samples from the 14 ROS-cGMPopathies of the ROCG cluster.

**Results:** Here, we investigate one of the best studied clusters of twelve heterogeneous cardiovascular, neuro-psychiatric, metabolic and pulmonary comorbid disease phenotypes of unmet medical need, defined—at least in part—by reactive oxygen species-induced dysfunction of and cGMP signalling. Hypertension and heart failure have been validated within the ROCG endotype in biobanked patient samples (1 out of 5 and 1 out of 3 patients, respectively). Next, in samples from the Estonian biobank, the prevalence of the ROCG endotype is described for each of the 14 ROS-cGMPopathies of the ROCG disease cluster.

**Conclusions:** Stratifying patients according to both phenotype and endotype will enable high precision therapeutic interventions, i.e. low number needed to treat. These data show that even for supposedly complex diseases, endotyping for precision medicine, and mechanistic disease re-definition is possible.

**Funding:** The research described in this review has been sponsored by the REPO-TRIAL project. The REPO-TRIAL project has received funding from the European Union’s Horizon 2020 research and innovation program under grant agreement No. 777111. This reflects only the author’s view, and the European Commission is not responsible for any use that may be made of the information it contains.

**References**

1. Schork, NJ 2015, ‘Personalized medicine: Time for one-person trials’, Nature, 520: 609–611.
2. Wieseler, B, McGauran, N, Kaiser, T 2019 ‘New drugs: where did we go wrong and what can we do better?’, BMJ, 366: l4340.
3. Nogales, C, Mamdouh, ZM, List, M, Kiel, C, Casas, AI, Schmidt, HHHW 2022, ‘Network pharmacology: curing causal mechanisms instead of treating symptoms’, Trends Pharmacol Sci., 43: 136–150.
4. Petraina, A, Nogales, C, Krahn, T, Mucke, H, Lüscher, TF, Fischmeister, R, et al. 2021, ‘Cyclic GMP modulating drugs in cardiovascular diseases: Mechanism-based network pharmacology’, Cardiovasc Res.
5. Langhauser, F, Casas, AI, Dao, V-T-V, Guney, E, Menche, J, Geuss, E, et al. 2018, ‘A diseasome cluster-based drug repurposing of soluble guanylate cyclase activators from smooth muscle relaxation to direct neuroprotection’, NPJ Syst Biol Appl., 4: 8

## Session 2 | Brain & Neurosensory System

## O5 Role of NO-GC1 and NO-GC2 in hippocampal cGMP responses induced by glutamatergic agonists

### Jan Giesen, Evanthia Mergia, Doris Koesling, Michael Russwurm

#### Ruhr-Universität Bochum, Pharmakologie, Bochum, Germany

##### **Correspondence:** Doris Koesling (doris.koesling@ruhr-uni-bochum.de)

*J Transl Med* 2022, **21(1)**:O5

NO-sensitive guanylyl cyclases (NO-GC) catalyse the formation of the signalling molecule cGMP that is involved in the regulation of various physiological cardiovascular events. In the central nervous system, nitric oxide is known to play an important role in synaptic transmission. Most of the effects of NO are mediated by NO-GCs, the receptors for NO, whose cGMP-forming activity is stimulated up to 200-fold upon NO binding to the enzymes prosthetic heme group. Two isoforms of NO-GC, NO-GC1 and NO-GC2, exist that exhibit comparable regulatory and enzymatic properties. An impairment of long-term potentiation in isoform-specific knockout (KO) mice demonstrated the importance of either NO-GC isoform for synaptic plasticity. Electrophysiological experiments in brain slices indicated pre- and postsynaptic localisation of NO-GC1 and NO-GC2 in hippocampal glutamatergic neurons and facilitation of neurotransmitter release and increase of NMDA receptor-mediated currents as physiological responses, respectively. In live cell fluorescence resonance energy transfer (FRET) imaging, NMDA receptor agonist-induced cGMP increases were demonstrated in cultured hippocampal neurons. Unexpectedly, NO-dependent NMDA receptor-independent cGMP signals were also brought about by stimulation of AMPA receptors with the calcium being supplied via L-type voltage gated calcium channels. Here, we asked which of the NO-GC isoform is responsible for NMDA- and AMPA-induced cGMP signals in hippocampal neurons and therefore monitored the respective FRET responses in neurons of NO-GC1- and NO-GC2-deficient mice. In addition, information on the impact of PDEs on the cGMP response was gathered by monitoring shaping of the cGMP signals by PDE inhibitors.

## O6 A localized scaffold for cGMP increaseis required for apical dendrite development in embryonic principal neurons

### Maya Shelly

#### Stony Brook University, Neurobiology and Behavior, New York New York, USA

##### **Correspondence:** Maya Shelly (maya.shelly@stonybrook.edu)

*J Transl Med* 2022, **21(1)**:O6

**Introduction:** An essential early event in mammalian embryonic brain development is neuronal polarization, in which neurons form the distinct compartments of the axon and dendrite. Axons and dendrites inherently differ in the molecular composition of their cytoplasm, cytoskeleton, and plasma membrane. These differences underlie the unique morphology and function of these compartments and are responsible for directed information flow in the brain. How polarity arises from seemingly equivalent neurites remains an outstanding question. Specification of the axon has dominated studies on neuron polarization, yielding an understanding of the molecular events underlying axonal identity, its specification and growth. However, the events in the polarizing neuron that lead to dendrite development have remained elusive. Studies in cultured neurons have established that axon specification instructs neuronal polarization and is necessary for dendrite development. However, dendrite formation in vivo occurs when axon formation is prevented. We find that apical dendrite development in principal pyramidal neurons is directed by spatially organized extrinsic cues and mediated by a localized cGMP-synthesizing complex. We show that the scaffolding protein Scribble associates with Plexin co-receptors for Semaphorin3A and the cGMP-synthesizing enzyme soluble-Guanylate-Cyclase (sGC). We find that the critical associations within the complex are necessary for distinctly higher cGMP generation in dendrites and for apical dendrite development in the CA1 region of the embryonic hippocampus. Local cGMP elevation or sGC expression rescued effects of Scribble knockdown on dendrite development indicating that Scribble is an upstream regulator of cGMP-activity. Together, we show that during neuronal polarization, apical dendrite development is directed by a scaffold that links signals of extracellular cues to localized cGMP increase.

**Methods:** cGMP-FRET, In Utero genetic manipulations in embryonic hippocampus, Biochemistry, Material engineering.

**Results**

- Dendrites have higher cGMP than the axon in a Scribble-dependent manner
- Scribble/sGC expression are enriched at apical dendrites
- Genetic manipulations within the Scribble complex caused severe defects in apical dendrite development
- Expressing sGC in developing CA1 neurons in vivo rescued effects of Scribble knockdown
- Scribble also associates with PlexinA3 co-receptor for Semaphorin3A
- PlexinA3-Scribble association is necessary for Sema3A-mediated cGMP increase in dendrites and for apical dendrite development

**Conclusions:** A common scaffold mediates signals of extracellular cues to localized cGMP increase to direct apical dendrite development during embryonic neuronal polarization (Figure 1).
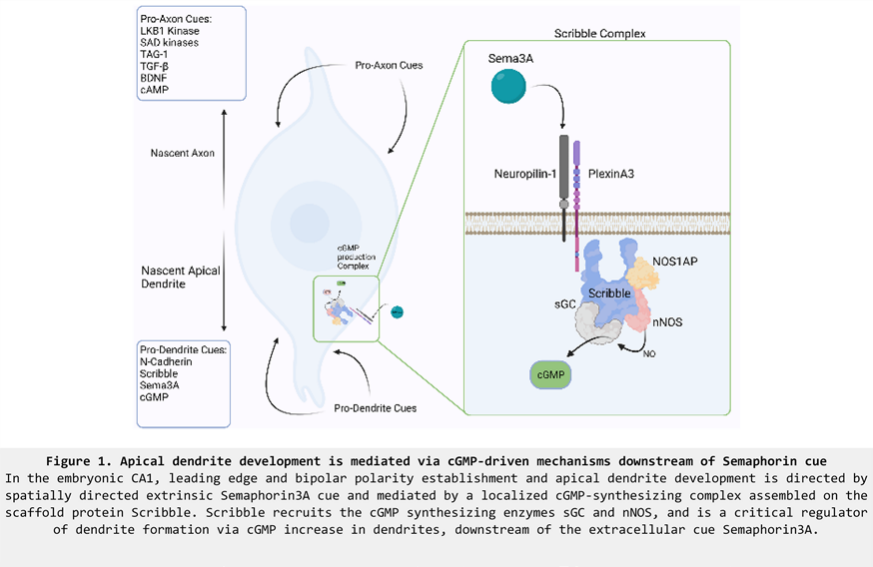


**Acknowledgements:** I thank the postdocs and students in my lab who have performed these studies: Joanna Szczurkowska, Seong Il Lee, Alan Guo, Jacqueline Martin, Chia Te Chien, Tamor Khan.

## O7 Structural basis of CNG channel function and channelopathy

### Jian Yang

#### Columbia University, Biological Sciences, New York New York, USA

##### **Correspondence:** Jian Yang (jy160@columbia.edu)

*J Transl Med* 2022, **21(1)**:O7

**Introduction:** Cyclic nucleotide-gated (CNG) channels convert light- and odorant-induced changes in intracellular cyclic nucleotides in into electrical signals and are essential for vision and smell. Numerous mutations in CNG channels cause visual disorders, including achromatopsia (ACHM) and retinitis pigmentosa. Native CNG channels are heterotetramers formed by CNGA and CNGB subunits.

**Methods:** To gain insights into the molecular mechanisms of CNG channel assembly, activation and permeation, we have solved the cryo-EM structures of a nematode CNG channel TAX-4 in apo and cGMP-bound states and the human cone photoreceptor CNG channel formed by CNGA3/CNGB3 in the apo state.

**Results:** These structures illustrate the conformational changes associated with cGMP gating and reveal a previously undescribed activation gate located in the central cavity (Fig. 1). The structure of the CNGA3/CNGB3 channel shows that the channel contains three CNGA3 and one CNGB3 subunits and highlights a critical role of CNGB3 in shaping cone CNG channel properties and responses. More than 150 and 125 mutations in *CNGA3* and *CNGB3*, respectively, are linked to ACHM. Using cryo-EM, liposome reconstitution, calcium imaging and a cell viability assay, we have investigated the structural, molecular and cellular effects of R410W, an ACHM-associated mutation in CNGA3. The R410W mutation produces complete color blindness and is reportedly a loss-of-function (LOF) mutation. Cryo-EM structures of TAX-4 carrying the analogous mutation R421W show that, paradoxically, R421W does not change the structure of cGMP-bound channel but opens the channel in the absence of cGMP. We also find that R421W/R410W increases the spontaneous activity of TAX-4 and CNGA3/CNGB3 channels reconstituted in liposomes and expressed in HEK293 cells, and that overexpression of R410W channels causes death of HEK293 and photoreceptor cells.

**Conclusions:** Our study sheds light on CNG channel allosteric gating, provides new frameworks for further mechanistic investigation of cone CNG channel physiology and channelopathy, and has implications for mutation-specific treatment of retinopathy.
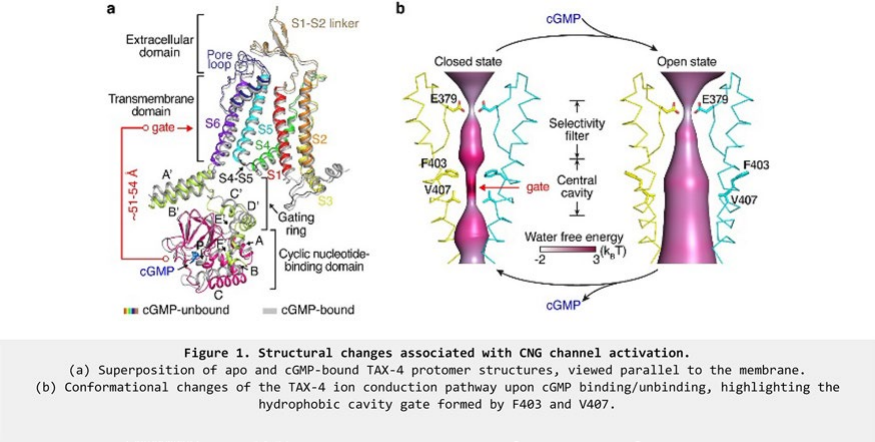


**Funding:** This work was supported by the National Institutes of Health grants RO1EY027800 and RO1GM085234 (J.Y.). Some of this work was performed at the Columbia University Cryo-Electron Microscopy Center (CUCEC) and at the Simons Electron Microscopy Center and National Resource for Automated Molecular Microscopy located at NYSBC. We thank members of CUCEC and NYSBC for support and assistance in cryo-EM grid screening and data acquisition.

## O8 Stress affects central compensation of neural responses to cochlear synaptopathy in a cGMP-dependent way

### Daria Savitska^1^, Morgan Hess^1^, Dila Calis^1^, Philine Marchetta^1^, Csaba Harasztosi^1^, Stefan Fink^1^, Philipp Eckert^1^, Peter Ruth^2^, Lukas Rüttiger^1^, Marlies Knipper^1^, Wibke Singer^1^

#### ^1^University of Tübingen, University ENT Clinic Tübingen, Molecular Physiology of Hearing, Tübingen Hearing Research Center, Tübingen, Germany; ^2^University of Tübingen, Department of Pharmacology, Toxicology and Clinical Pharmacy, Institute of Pharmacy, Tübingen, Germany

##### **Correspondence:** Daria Savitska (daria.savitska@uni-tuebingen.de)

*J Transl Med* 2022, **21(1)**:O8

**Introduction:** Increasing evidence supports a link between hearing loss and dementia. We previously demonstrated in a mouse model that an age-related cochlear synaptopathy (decoupling of inner hair cell synapses from auditory nerve fibers) leads to poorer temporal auditory and memory-related processing. We could show that cochlear synaptopathy can, in some individual cases, be centrally compensated through enhanced input/output function of auditory brainstem responses (neural gain), preventing an age-dependent temporal discrimination loss [1].

**Methods:** Therefore, mice can be subdivided by their compensation capacity into a group of *low compensators* and another group of *high compensators*.

**Results:** *Low compensators* also displayed an associated decrease in memory-linked processes and recruitment of activity-dependent brain-derived neurotrophic factor (BDNF) in hippocampal regions in comparison to *high compensators*. We aimed to identify factors capable of modifying this compensation mechanism. Animals were injected with either a cGMP-stimulating drug—the “memory-enhancing” phosphodiesterase 9A inhibitor—or a placebo.

**Conclusions:** We surprisingly found that the successful central auditory- and memory-dependent adjustment to cochlear synaptopathy is a cGMP- and glucocorticoid-dependent process.

**Funding:** This work was funded by the Deutsche Forschungsgemeinschaft (DFG, German Research Foundation) Research Training Group GRK 2381 Projektnummer 335549539; FOR 2060 project RU 713/3-2, SPP 1608 RU 316/12-1, KN 316/12-1 and the Sigmund Kiener Stiftung.

**Reference**

1. Marchetta, P., Savitska, D., Kübler, A., Asola, G., Manthey, M., Möhrle, D., Schimmang, T., Rüttiger, L., Knipper, M., & Singer, W. 2020, ‘Age-Dependent Auditory Processing Deficits after Cochlear Synaptopathy Depend on Auditory Nerve Latency and the Ability of the Brain to Recruit LTP/BDNF’, *Brain sciences*, Vol. 10,10 710

## Sesssion 3 | Clinical Translation/Cardiovascular

## O9 Lumpers and splitters: the legacy and future of heart failure management

### Thomas F. Lüscher

#### Royal Bormpton Hospital, Department of Cardiology, Cardiology I, London, UK

##### **Correspondence:** Thomas F. Lüscher (cardio@tomluescher.ch)

*J Transl Med* 2022, **21(1)**:O9

*Published in: Eur Heart J, 2019;40:3292–3296.* https://doi.org/10.1093/eurheartj/ehz644.

## O10 Travels with vericiguat: insights from a clinical scientist

### Paul Armstrong

#### Department of Medicine (Cardiology), University of Alberta, Edmonton, Alberta, Canada

##### **Correspondence:** Paul Armstrong (parmstro@ualberta.ca)

*J Transl Med* 2022, **21(1)**:O10

The phase 3 VICTORIA trial, in 5050 patients with heart failure and reduced ejection fraction (HFrEF) who had a recent worsening HF event, demonstrated a clinically relevant, statistically significant reduction in the primary composite endpoint of cardiovascular death and heart failure hospitalization. Given the unusually high clinical event rate -since learning of the principal results—our priority over the past 2 years has been to establish a context for the trial with other recent heart failure studies and to understand potential heterogeneities in the response to vericiguat.

This presentation will provide further insights into the following:

- The three clinical trial subsets—characterized by the relationship between their recency and nature of their heart failure event- and how this related to clinical outcomes and vericiguat’s efficacy
- The prognostic significance of the NT-proBNP, troponin T levels and other biomarkers at randomization and what insights they provide to patient subgroups and treatment effect
- The impact of vericiguat on sequential NT-proBNP levels and what relationship these changes have on the treatment effect
- The safety of vericiguat as measured by renal function, potassium and hemoglobin

These findings are met to broaden the understanding of vericiguat’s role in addressing the unmet needs of patients with heart failure and inform the path for future investigation of this novel pathway.

## O11 Genetic variation affecting cGMP signaling in atherosclerosis and myocardial infarction

### Thorsten Kessler

#### Technical University of Munich, German Heart Centre Munich, Munich, Germany

##### **Correspondence:** Thorsten Kessler (thorsten.kessler@tum.de)

*J Transl Med* 2022, **21(1)**:O11

**Introduction:** Coronary atherosclerosis and myocardial infarction are leading causes of morbidity and mortality worldwide. In addition to so-called traditional risk factors, e.g. diabetes, hypertension, or smoking, a positive family history has been known to be associated with increased risk for decades. Large-scale genetic studies identified genetic variants in more than 200 genes to underlie this observation. Importantly, several variants are located in genes that are involved in cGMP signaling.

**Methods:** Our group used genetic analyses, in vitro and in vivo experimentation to investigate the functional role of genetic variants in genes that are involved in cGMP signaling in atherosclerosis.

**Results:** We identified rare coding variants in the genes encoding the soluble guanylyl cyclase (sGC) and phosphodiesterase 5A (PDE5A) to be associated with coronary artery disease. Importantly, these genes furthermore habour common, non-coding variants which influence expression of the genes and thereby modulate cGMP signaling in atherosclerosis. Our findings particularly point to a functional role of genetic variation in sGC and PDE5A in platelets and vascular smooth muscle cells, respectively.

**Conclusions:** Clustering of genetic variants which are associated with coronary artery disease and myocardial infarction in genes that are crucially involved in cGMP signaling highlights the importance of this pathway in atherosclerosis and opens up new vistas for prevention and therapy of the disease.

## O12 Mutant ANP induces mitochondrial and ion channel remodeling in a human iPSC-derived atrial fibrillation model

### Dawood Darbar

#### University of Illinois at Chicago, Chicago, USA, Medicine/Cardiology, Chicago Illinois, USA

##### **Correspondence:** Dawood Darbar (darbar@uic.edu)

*J Transl Med* 2022, **21(1)**:O12

*Published in: JCI Insight, 2022; 7:e155640.* https://doi.org/10.1172/jci.insight.155640.

**Funding:** This work was in part supported by R01 HL150586 (D.D. and S.R.K), R01 HL148444 (D.D.), T32 HL139439 (D.D.), and R01-HL126516 (J.R).

## O13 Elderly, postmenopausal females with heart failure with preserved ejection fraction (HFpEF) are responsive to soluble guanylate cyclase (sGC) stimulation: a post hoc analysis of the CAPACITY trial

### Andreas Busch, Phebe Wilson, G. Todd Milne, Albert T. Profi

#### Cyclerion Therapeutics, Cambridge Massachusetts, USA

##### **Correspondence:** Andreas Busch (abusch@cyclerion.com)

*J Transl Med* 2022, **21(1)**:O13

**Introduction:** Impaired nitric oxide (NO)-sGC-cGMP signaling has been implicated in the pathogenesis of HFpEF. In females, the prevalence of HFpEF increases post menopause, and > 65% of HFpEF patients older than 75y are female. Compared to males, females with HFpEF have more diastolic dysfunction, are more symptomatic, and have a poorer quality of life. Previous clinical studies have shown that females with HFpEF are more responsive than males to spironolactone and sacubitril/valsartan, treatments which can increase cGMP levels. Aging and reduced estrogen levels may act together to compromise NO-sGC-cGMP signaling in elderly, postmenopausal females. The CAPACITY HFpEF study (Udelson et al., JAMA, 2020) evaluated the effect of the sGC stimulator praliciguat on exercise capacity in male and female HFpEF patients with left ventricular ejection fraction ≥ 40%, reduced exercise tolerance, and 2 or more concomitant conditions associated with decreased NO-cGMP signaling (hypertension, diabetes/pre-diabetes, obesity, or advanced age). In the full population (N = 143), there was no significant effect on the primary efficacy endpoint, change from baseline in peak oxygen consumption (peak VO_2_) to week 12, measured using cardiopulmonary exercise testing.

**Methods:** We performed a post hoc analysis of the CAPACITY HFpEF data for the subgroup of females aged > 70 years (N = 34).

**Results:** In the subgroup of female participants > 70 years, praliciguat treatment led to a clinically meaningful improvement in peak VO_2_ compared to placebo (least-squares mean difference 1.6 mL O_2_/kg/min, nominal p = 0.007). Furthermore, in this elderly female subgroup, a higher proportion of praliciguat than placebo recipients (50% vs. 11%) showed a clinically meaningful (> 1.0 mL O_2_/kg/min) improvement in peak VO_2_, independent of baseline exercise capacity. Directional improvements in other measures of functional capacity, including ventilatory efficiency, peak workload, and 6-min walk test distance were also observed in the elderly female subgroup. Consistent with a greater pharmacological response, praliciguat treatment was associated with larger reduction in blood pressure in elderly females than in the full population. Praliciguat treatment was well tolerated in the elderly female subgroup and overall.

**Conclusions:** Elderly, postmenopausal females with HFpEF represent a HFpEF phenotype that may benefit from sGC stimulation. Prospective clinical trials in this population are warranted.

## Session 4 | Cardiovascular Diseases and cGMP

## O14 Nitroxyl induces blood pressure lowering in part by oxidation of cGMP-dependent protein kinase I

### Friederike Cuello

#### UKE, Experimental Pharmacology and Toxicology, Hamburg, Germany

##### **Correspondence:** Friederike Cuello (f.cuello@uke.de)

*J Transl Med* 2022, **21(1)**:O14

Hypertension is an important risk factor for cardiovascular complications. Effective pharmacological blood pressure lowering reduces end-organ damage and the risk of morbidity and mortality in patients. Nitroxyl released from donor molecules is in clinical development for the treatment of cardiovascular disease. Defining molecular mechanisms that contribute to their beneficial effect, may help to optimise their use. We have previously demonstrated that nitroxyl-mediated vasodilation is at least in part caused by oxidation of cysteine 117 and 195 in cGMP-dependent protein kinase I (PKGI). We aimed to determine whether nitroxyl donors lower blood pressure via this novel PKGI activation mechanism in vivo.

## O15 cGMP-dependent protein kinase I alpha substrates in the regulation of left ventricular function

### Robert M. Blanton

#### Tufts Medical Center, Molecular Cardiology Research Institute, Boston, USA

##### **Correspondence:** Robert M. Blanton (rblanton@tuftsmedicalcenter.org)

*J Transl Med* 2022, **21(1)**:O15

**Introduction:** The cGMP-dependent protein kinase 1 alpha (cGK1a) is required for normal left ventricular functional compensation to pressure overload^1^. These effects require an intact cGK1a leucine zipper domain^1^. We recently identified the Mixed Lineage Kinase 3 (MLK3) as co-interacting with cGK1a and as required for the therapeutic effects of cGMP augmentation in LV pressure overload^2^. MLK3 whole-body gene deletion also induces hypertension^2^. The cardiac myocyte (CM)-specific, blood pressure independent contribution of MLK3 to LV function remains unknown.

**Methods:** We generated and examined the cardiac phenotype of mice with CM-specific MLK3 deletion. LoxP sites were inserted by homologous recombination around exons 4–10 of the *map3k11* gene, resulting in MLK3 floxed (MLK3^fl/fl^) mice. We crossed the MLK3^fl/fl^ mice with the aMHC-Cre transgenic strain to enable CM-restricted MLK3 deletion. We studied MLK3^fl/fl^ x aMHC Cre+ and MLK3^fl/fl^ x aMHC Cre− littermates (termed MLK3^CM-KO^ and control, respectively).

**Results:** In isolated CMs, PCR confirmed MLK3 excision in MLK3^CM-KO^ but not control mice. We observed no MLK3 gene excision in spleen tissue of the MLK3^CM-KO^, supporting specificity of deletion. Blood pressure did not differ between the MLK3^CM-KO^ mice compared with control littermates. MLK3^CM-KO^ mice of both sexes displayed basal reduction in LV function as assessed by invasive hemodynamics. Unexpectedly, only female MLK3^CM-KO^ mice developed age-dependent LV dilation and overt dysfunction detected by echocardiography.

**Conclusions:** We interpret these findings to reveal a CM-specific role of the cGK1a effector MLK3 as a tonic regulator of LV structure and function in vivo, which is independent of the MLK3 effect on blood pressure. Our findings also suggest sex-specific cardiovascular effects of MLK3. These findings support that investigating cGK1a substrates in the LV can reveal new mechanisms of relevance to heart failure.

**Funding:** This study was funded by the NIH R01HL131831.

**References**

1. Blanton RM, Takimoto E, Lane AM, Aronovitz M, Piotrowski R, Karas RH, Kass DA, and Mendelsohn ME. Protein kinase g iα inhibits pressure overload-induced cardiac remodeling and is required for the cardioprotective effect of sildenafil in vivo. *J Am Heart Assoc*. 2012;1:e003731.
2. Calamaras TD, Pande S, Baumgartner RA, Kim SK, McCarthy JC, Martin GL, Tam K, McLaughlin AL, Wang GR, Aronovitz MJ, Lin W, Aguirre JI, Baca P, Liu P, Richards DA, Davis RJ, Karas RH, Jaffe IZ, Blanton RM. MLK3 mediates impact of PKG1a on cardiac function and controls blood pressure through separate mechanisms. *JCI Insight*. 2021 Sep 22;6(18):e149075.

## O16 PDE9 inhibition for the treatment of obesity and cardiometabolic syndrome

### David A. Kass, Sumita Mishra

#### Johns Hopkins, Medicine/Cardiology, Baltimore Maryland, USA

##### **Correspondence:** Sumita Mishra (smirshra1@jhmi.edu)

*J Transl Med* 2022, **21(1)**:O16

**Introduction:** Central obesity with cardiometabolic syndrome (CMS) is a global pandemic and major contributor to human disease. While exercise and diet are important, they are often insufficient to counter severe obesity, and new and effective therapies are sorely needed.

**Methods:** We studied the role of phosphodiesterase type 9—a highly selective regulator of cGMP—on severe obesity and cardiometabolic syndrome in mice. The model includes 4 months of high fat diet (or control diet) feeding in C57BL/6N males and females (latter ± ovariectomy); superimposition of mild pressure overload by aortic constriction at that time, and then randomization to placebo of the PDE9 inhibitor PF-04447943. Cell and tissue analysis of fat metabolism, signaling, and regulation by PDE9 are explored.

**Results:** We found that inhibiting the highly cyclic-GMP selective phosphodiesterase-type 9A (PDE9-I) in male and ovariectomized female mice suppresses pre-established severe diet-induced obesity/CMS. This treatment was effective in mice with or without superimposed mild cardiac pressure-load. PDE9-I reduced total body, inguinal, hepatic, and myocardial fat, increased whole body metabolism, and improved CMS, all without significantly altering activity or food intake. PDE9 localizes at mitochondria, and its inhibition in vitro stimulates lipolysis and mitochondrial respiration in adipocytes and myocytes. This requires upregulation of multiple fatty acid uptake and metabolism genes linked to PPARa, and PPARa upregulation was required in vitro and in vivo to achieve lipolytic, anti-obesity, and metabolic effects from PDE9-I. There was a striking sex-dimorphism, as these PDE9-I induced changes did not occur in obese/CMS but non-ovariectomized females. This may relate to altered PPARa chromatin binding in the presence of co-activated estrogen receptor-a.

**Conclusions:** Clinical PDE9-I have been tested in humans already for neurocognitive and sickle cell disease and is in ongoing trials for heart failure. If effective against obesity/CMS, this would greatly expand its therapeutic utility.

## O17 Illuminating local cGMP signaling for better cardioprotection

### Viacheslav Nikolaev

#### University Medical Center Hamburg-Eppendorf, Institute of Experimental Cardiovascular Research, Hamburg, Germany

##### **Correspondence:** Viacheslav Nikolaev (v.nikolaev@uke.de)

*J Transl Med* 2022, **21(1)**:O17

**Introduction:** cGMP is a key second messenger with well-accepted cardioprotective functions. However, we are still far from understanding the underlying mechanisms to tailor more specific therapeutics for severe cardiovascular diseases such as heart failure. In particular, comprehensive information on spatial and temporal patterns of cGMP signaling in functional subcellular nanodomains of cardiomyocytes and differences in compartmentation of signals originating from various guanylyl cyclases such as different natriuretic peptide receptors and NO-sensitive cyclase (NO-GC) have been lacking.

**Methods:** We have developed and applied state-of-the-art live cell imaging techniques including new Förster resonance energy transfer (FRET) based biosensors and non-optical nanoscale imaging using scanning ion conductance microscopy (SICM) to better understand real time cGMP dynamics in heathy and diseased mouse, rat and human cardiomyocytes.

**Results:** Using a transgenic mouse model expressing a highly sensitive cGMP biosensor red cGES-DE5 in the heart and real time SICM/FRET microscopy we could show that natriuretic peptide receptors GC-A (also known as NPR1, receptor for atrial natriuretic peptide) and GC-B (also known as NPR2, receptor for the C-type natriuretic peptide) are differently localized on cardiomyocyte membrane. The former is exclusively found in T-tubules and the latter in both T-tubules and outer cell membrane domains called crests. These distinct localization patterns lead to different compartmentation of the respective cGMP signals which are either locally confined to T-tubular membranes not affecting cardiac contractility (GC-A) or are diffusible along larger distances leading to phophorylation of phospholamban and troponin I to enhance cardiac relaxation. Also, we could show that beta3-adrenergic receptors coupled to NO-GC signalosome can be found only in T-tubules of healthy myocytes, but they undergo redistribution to cell crests in failing cells. Finally, we studied cGMP signals under hypoxic and ischemic conditions in mouse und human myocytes and found that these conditions lead to an increase of cardiomyocyte cGMP levels due to partial autophagosomal degradation of the phosphodiesterase 3A. Interestingly, this mechanism leads to increased cardioprotection, diminished apoptosis and enhanced responses to clinically relevant NO-GC activators.

**Conclusions:** Our live cell imaging approach could uncover new mechanisms of cardiomyocyte cGMP compartmentation which can be potentially targeted for heart failure therapy.

## O18 Long term delivery of CNP in a mouse model of heart failure with preserved ejection fraction

### Dulasi Arunthavarajah^1^, Bernadin Dongmo Ndongson^1^, Gaia Calamera^1^, Jan M. Aronsen^2^, Finn Olav Levy^1^, Kjetil W. Andressen^1^

#### ^1^University of Oslo and Oslo University Hospital, Department of Pharmacology, Institute of Clinical Medicine, Oslo, Norway; ^2^University of Oslo and Oslo University Hospital, Department of Molecular Medicine, Institute of Basic Medical Sciences, Oslo, Norway

##### **Correspondence:** Dulasi Arunthavarajah (dulasi.arunthavarajah@medisin.uio.no)

*J Transl Med* 2022, **21(1)**:O18

**Introduction:** Heart failure (HF) is a major health burden Worldwide. Approximately 50% of patients suffers from HF with preserved ejection fraction (HFpEF), where often hearts are stiff and not readily filled with blood. Although several advances have been made in the understanding and treatment of HF with reduced ejection fraction (HFrEF), there are few effective treatment options for HFpEF patients. We have previously shown that activation of the Guanylyl Cyclase B (GC-B) with C-type natriuretic peptide (CNP) increases cGMP in cardiomyocytes that lead to increased removal of Ca2+ into the sarcoplasmic reticulum, faster relaxation and phosphorylation of titin that leads to more compliant cardiomyocytes and increased diastolic filling.

**Methods:** To induce HFpEF, C57BL/6N mice were fed a combination of high fat (HFD) provoking metabolic stress and administered an inhibitor of nitric oxide synthase (L-NAME) in the drinking water to induce hypertensive stress. Mice receiving maintenance diet (MD) were included as control. For treatment, osmotic pumps containing either CNP or vehicle were subcutaneously implanted in mice prior to dietary manipulation and mice were treated for 6 weeks. We measured blood pressure, performed echocardiography and pressure–volume catheterization and excised hearts and lungs after euthanasia.

**Results:** Osmotic pumps delivering CNP resulted in over fivefold increase in circulating plasma concentrations of CNP compared to vehicle-treated mice. In HFD+L-NAME mice, CNP reduced cardiomyocyte area, indicative of reduced hypertrophy. CNP-treated mice had lower systolic blood pressure. A non-significant trend towards decreased lung weight was also noted in the group of mice receiving CNP-treatment, indicative of reduced lung congestion.

**Conclusions:** These preliminary results could indicate beneficial effects of CNP treatment in HFpEF mice.

## Session 5 | Other Indications & Non-Canonical Signaling

## O19 New aspects of NP/cGMP signaling in adipocytes and energy expenditure

### Sheila Collins

#### Cardiovascular Medicine & Molecular Physiology and Biophysics, Vanderbilt University Medical Center, Nashville, TN, USA

##### **Correspondence:** Sheila Collins (sheila.collins@vumc.org)

*J Transl Med* 2022, **21(1)**:O19

The role of the cardiac natriuretic peptides (NPs) and cGMP in adipocyte metabolism serves as a mechanism to increase mobilization of stored energy by stimulating lipolysis as well as activating and expanding the amount of energy-expending thermogenic brown adipocytes. Our earlier work showed that this pathway can be beneficial for lowering blood glucose and fatty acid levels to mitigate type II diabetes and reduce adipose mass. In addition to raising cGMP by NPs, cGMP levels can be maintained by preventing their degradation by phosphodiesterases (PDEs) such as PDE9. This presentation will discuss signaling mechanisms by NPs and PDE9, their regulation, and consequences on adipose tissue and metabolic health.

## O20 Retinopathy caused by dysfunction of cGMP effectors: pathobiology and treatment options

### Stylianos Michalakis

#### LMU Munich, University Hospital, Department of Ophthalmology, Munich, Germany

##### **Correspondence:** Stylianos Michalakis (stylianos.michalakis@cup.uni-muenchen.de)

*J Transl Med* 2022, **21(1)**:O20

Inherited retinal degenerations are eye diseases that lead to severe visual impairment and even blindness. They are caused by defects in genes encoding key proteins of the visual process, whose central second messenger is cyclic guanosine monophosphate (cGMP). Here, I will focus on two specific retinopathies caused by mutations in two cGMP effectors specifically expressed in rod photoreceptors: the cyclic nucleotide-gated channel beta 1 subunit (CNGB1) and the photoreceptor phosphodiesterase 6 alpha (PDE6A) subunit. Mutations in the *CNGB1* and the *PDE6A* genes cause retinitis pigmentosa type 45 and 43, respectively. The evolution of powerful gene therapy vectors based on non-pathogenic recombinant adeno-associated viruses (AAV) has enabled the development of potentially curative therapeutic approaches for these previously incurable ocular diseases.

In my presentation, I will provide an overview of the underlying pathobiology, outline the preclinical and clinical development of current gene therapy approaches, and finally discuss remaining challenges and options for future improvements.

## O21 Protein kinase G2 is required for androgen receptor signaling in bone

### Hema Kalyanaraman, Shyamsundar Pal China, Renate Pilz

#### University of California, San Diego, Department of Medicine, La Jolla California, USA

##### **Correspondence:** Hema Kalyanaraman (hkalyanaraman@ucsd.edu)

*J Transl Med* 2022, **21(1)**:O21

**Introduction:** Androgens and estrogens are key regulators of sex differences in bone, but androgen signaling in osteoblasts has received little attention. We previously showed that NO/cGMP/protein kinase G (PKG) signaling mediates anabolic effects in bone, in part by regulating the Wnt/β-catenin pathway, which plays a key role in skeletal homeostasis (1).

**Methods:** We generated mice with osteoblast-specific (OB) knockout (KO) of type 2 protein kinase G (OB *Prkg2-*KO mice) by crossing mice carrying floxed *Prkg2* alleles with transgenic mice expressing CRE recombinase under control of the osteoblast-specific 2.3 kb Col1a1 promoter, which is active in mesenchymal stem cells committed to the osteoblast lineage, in osteoblasts, and mature osteocytes. The skeletal phenotype of these mice was characterized by micro-CT, static and dynamic histomorphometry, and gene expression profiling by qRT-PCR, and was compared to control litter mates (genotype *Prkg2 f/f*) (2). Primary osteoblasts were isolated from the long bones of 12 week-old mice, and their growth, survival, and differentiation was examined as described (3). Cells were treated with dihydrotestosterone (DHT, which cannot be converted to estrogens) or 17β-estradiol in phenol red-free medium containing 0.1–1% charcoal-stripped fetal bovine medium.

**Results:** The skeletal phenotype of OB *Prkg2-*KO mice demonstrated a striking sexual dimorphism: male OB *Prkg2-*KO mice had fewer osteoblasts, reduced bone formation rates, and lower trabecular and cortical bone volumes compared to control littermates, whereas female OB *Prkg2-*KO mice showed no bone abnormalities. Osteoclast parameters were unaffected by genotype in both sexes. Expression of differentiation-related and Wnt/β-catenin-related genes was lower in osteoblasts and bones of male KO but not female KO mice compared to control mice. Dihydrotestosterone (DHT) increased expression of β-catenin and its target genes via PKG2 in male osteoblasts, whereas 17β-estradiol increased expression of these genes independently of PKG2 in osteoblasts of both sexes, with larger effects in female cells. DHT stimulation of gene expression was initiated at the plasma membrane where the androgen receptor, PKG2, and Src co-localized in caveolin-1-enriched membrane fractions. Maximal DHT induction of target genes required PKG2 activation of Src, ERK1/2, and Akt kinases and cooperation between membrane- and DNA-bound androgen receptors.

**Conclusions:** These data reveal a novel cross-talk between PKG2 and membrane-bound androgen receptor, and explain the skeletal sexual dimorphism of OB *Prkg2-*KO mice.

**Funding:** This work was supported by the National Institutes of Health grants R01-AR-068601 and R01-AG070778 (to RBP) and P30-NS047101 (UCSD Microscopy Shared Facility).

**References**

1. Kalyanaraman, H., Schall, N., and Pilz, R.B. (2018a). Nitric oxide and cyclic GMP functions in bone. Nitric Oxide *76*, 62–70.
2. Kalyanaraman, H., Ramdani, G., Joshua, J., Schall, N., Boss, G.R., Cory, E., Sah, R.L., Casteel, D.E., and Pilz, R.B. (2017). A Novel, Direct NO Donor Regulates Osteoblast and Osteoclast Functions and Increases Bone Mass in Ovariectomized Mice. J. Bone Miner. Res *32*, 46–59.
3. Kalyanaraman, H., Schwaerzer, G., Ramdani, G., Castillo, F., Scott, B.T., Dillmann, W., Sah, R.L., Casteel, D.E., and Pilz, R.B. (2018b). Protein Kinase G Activation Reverses Oxidative Stress and Restores Osteoblast Function and Bone Formation in Male Mice With Type 1 Diabetes. Diabetes *67*, 607–623.

## O22 Cyclic CMP and cyclic UMP in bacterial immunity

### Benjamin R. Morehouse^1,2^, Nitzan Tal^3^, Nora K. McNamara-Bordewick2^2^, Alexander F.A. Keszei^4^, Sichen Shao^4^, Philip J. Kranzusch^1,2,5^, Rotem Sorek^3^

#### ^1^Harvard Medical School, Department of Microbiology, Boston Massachusetts, USA; ^2^Dana-Farber Cancer Institute, Department of Cancer Immunology & Virology, Boston Massachusetts, USA; ^3^Weizmann Institute of Science, Department of Molecular Genetics, Rehovot, Israel; ^4^Harvard Medical School, Department of Cellular Biology, Boston Massachusetts, USA; ^5^Parker Institute for Cancer Immunotherapy, Dana-Farber Cancer Institute, Boston Massachusetts, USA

##### **Correspondence:** Benjamin R. Morehouse (benjamin_morehouse@dfci.harvard.edu)

*J Transl Med* 2022, **21(1)**:O22

**Introduction:** The cyclic purine mononucleotides cGMP and cAMP have varied and critical functions as second messengers in many biological systems. By contrast, the cyclic pyrimidine mononucleotides cCMP and cUMP have no known defined physiological roles. Bacteria are in a constant battle with bacteriophage, the viruses that infect them, and have thus evolved numerous mechanisms by which to defend themselves. We made the unexpected discovery that bacteria use cCMP and cUMP as signaling molecules mediating immunity against viruses and have provided the first biochemical and structural description of the pathways in which these compounds are involved.

**Methods:** We used a combination of approaches to characterize the function of these bacterial immune systems including: recombinant protein expression and purification, enzymatic assays, X-ray crystallography, phage infectivity assays, and high-resolution mass spectrometry.

**Results:** We found that many bacteria harbor enzymes that share structural homology with class III adenylate/guanylate cyclases and yet produce cCMP or cUMP in response to phage infection. Both cCMP and cUMP bind to specialized effector proteins encoded adjacent to the cyclase within immune defense operons we name Pycsar (Pyrimidine cyclase system for antiphage resistance). One subset of Pycsar effectors rapidly form ordered filamentous structures upon selective recognition of the cyclic pyrimidine signal. Filament formation leads to enzymatic degradation of the metabolite NAD^+^ and this ultimately causes defects in cell growth and prevents phage replication.

**Conclusions:** Our results provide biological relevance for cCMP and cUMP as second messengers in bacterial immunity. Pycsar is just one of several recently identified immune pathways in prokaryotic genomes that use nucleotide and NAD^+^-derived signaling molecules to confer protection against viruses. It remains an open question if these particular signals mediate antiviral defense in other organisms or play roles outside of immunity.

**Acknowledgements:** We thank the members of the Kranzusch and Sorek labs for helpful discussions and the Molecular Electron Microscopy Suite at Harvard Medical School. R.S. was supported in part by the European Research Council (ERC-CoG 681203), the Ernest and Bonnie Beutler Research Program of Excellence in Genomic Medicine, German Research Council (DFG) Priority Program SPP 2330 (SO 1611/2), the Minerva Foundation with funding from the Federal German Ministry for Education and Research, the Knell Family Center for Microbiology, the Yotam Project and the Weizmann Institute Sustainability and Energy Research (SAERI) initiative, and the Dr. Barry Sherman Institute for Medicinal Chemistry. P.J.K. was supported by grants from the Pew Biomedical Scholars Program, the Mark Foundation for Cancer Research, The Mathers Foundation, the Parker Institute for Cancer Immunotherapy, and a Burroughs Wellcome Fund PATH award. B.R.M. was supported as a Ruth L. Kirschstein NRSA Postdoctoral Fellow (NIH F32GM133063). S.S. was supported by grants from the Vallee Scholars Program and a Packard fellowship.

## O23 Understanding the regulation and function of the cGMP pathway for cell movement and chemotaxis in Dictyostelium

### Peter van Haastert

#### Groningen, Cell Biochemistry, Groningen, Netherlands

##### **Correspondence:** Peter van Haastert (p.j.m.van.haastert@rug.nl)

*J Transl Med* 2022, **21(1)**:O23

Dictyostelium is a eukaryotic unicellular organism that moves like neutrophils with pseudopodia. Cells of both organisms are guided to bacteria by chemoattractants. Despite their long evolutionary separation about one billion years ago, neutrophils and Dictyostelium move in a similar fashion with branched F-actin filled protrusions in the front; parallel F-actin and myosin II filaments in the cell body inhibit protrusions in the back of the cell. F-actin and myosin are regulated in Dictyostelium by a unique cGMP pathway that evolved independently from the cGMP pathway of mammals. It consists of two G-protein-regulated guanylyl cyclases that are homologous to twelve-transmembrane adenylyl cyclase and soluble adenylyl cyclase, respectively. There is only one cGMP-target: a very large protein consisting of many domains with a complex intramolecular activation mechanism: cGMP binding activates a GEF domain that induces the GDP/GTP exchange of a Ras domain which activates its adjacent kinase domain. cGMP is degraded by a cGMP-stimulated phosphodiesterase with a unique catalytic domain. The soluble guanylyl cyclase, the cGMP-binding protein and the cGMP-phosphodiesterase are all found in a protein complex with the G-protein subunit Gbeta in the front of the cell. The cGMP pathway has two functions: the protein complex in the front of the cell promotes the formation of branched F-actin, while the produced cGMP rapidly diffuses into the cell body where it promotes myosin filament formation and pseudopod inhibition in the rear of the cell. Together these two functions lead to persistent cell movement and efficient chemotaxis.

## Session 6 | NO-GC Structure, Modulators, Function

## O24 sGC maturation to function: its mechanism, regulation, dynamics, and biological relevance

### Dennis Stuehr

#### The Cleveland Clinic, Inflammation and Immunity, Cleveland Ohio, USA

##### **Correspondence:** Dennis Stuehr (stuehrd@ccf.org)

*J Transl Med* 2022, **21(1)**:O24

**Introduction:** Nitric oxide (NO)-cGMP signaling relies on NO to activate the NO receptor protein soluble guanylyl cyclase (sGC). To mature to the functional state, the sGCβ subunit must first obtain cellular heme, which allows it to bind with a sGCα subunit to form the functional heterodimer. Our studies shed light on how cellular heme is allocated and inserted into apo-sGCβ, how this drives the formation of the functional enzyme, the dynamics of these processes, how they are regulated by biological molecules and pharmacologic agents, their relation to the general cell heme allocation, and their relevance in determining the level of functional sGC in biology.

**Methods:** Our experiments typically involve cell cultures, purified recombinant proteins, and tissue samples. We employ a variety of biochemical and biophysical approaches including UV–vis, fluorescence, and mass spectroscopies. We employ typical molecular and cell biology methods and focus on making time-resolved measures whenever possible.

**Results:** Our studies with a variety of cells and tissues revealed that a major portion of their sGC (≥ 50%) naturally exists in its immature heme-free form, where the apo-sGCβ subunit is complexed with chaperone hsp90 and is separate from the sGCα subunit. This goes against the notion that sGC predominantly exists in its mature heterodimer form. In cells, heme delivery to apo-sGCβ depends on a GAPDH-heme complex, and heme insertion depends on the bound hsp90. After heme insertion takes place the hsp90 falls off and this exposes regions on sGCβ that can interact with sGCα to form the heterodimer. The heme delivery and insertion processes can be blocked by pharmacologic hsp90 inhibitors and by relatively high levels of NO, but are actually enhanced by lower levels of NO. The NO effect on heme allocation to apo-sGCβ is rapid, occurs in response to signaling levels of NO, and results in the formation of functional sGC heterodimer. The NO-driven heme allocation also depends on GAPDH and hsp90, the same machinery that cells utilize for their heme delivery during normal sGC maturation.

**Conclusions:** The natural existence of heme-deficient sGC provides an additional way that cells or tissues can control their level of functional sGC by controlling heme allocation. Heme allocation to apo-sGCβ involves GAPDH and hsp90 and causes a change in sGCβ protein partners that leads to the formation of the functional heterodimer. The ability of low biological levels of NO to quickly trigger heme allocation to apo-sGCβ identifies a new way that NO can regulate the sGC-cGMP signaling pathway, namely by triggering the assembly of its own receptor.

**Acknowledgements:** I am grateful for the excellent work by our lab members and collaborators and for funding support from the NIH.

## O25 A potential role of GUCY1A2 the human gene encoding the α_2_-subunit of nitric oxide sensitive guanylyl cyclase in cancer

### Sönke Behrends

#### Technical University Braunschweig, Department of Pharmacology, Braunschweig Lower Saxony, Germany

##### **Correspondence:** Sönke Behrends (s.behrends@tu-bs.de)

*J Transl Med* 2022, **21(1)**:O25

**Introduction:** GUCY1A2 has been suggested as a candidate cancer gene as early as in 2006 [1]. The statistical methods used in this large scale study have been criticized and the role of genes like GUCY1A2 with no prior established role in cancer pathogenesis have been met with skepticism [2]. We have established that NO-GC2, the complex of the α_2_-subunit and the β_1_-subunit of nitric oxide sensitive guanylyl cyclase is directed to cell–cell contacts in HEK293 cells [3] and other cell lines [unpublished data]. In addition, we have shown that the complex of the α_2_-subunit and the β_1_-subunit interacts with the tumor-suppressor protein scribble [3]. While we are also highly interested in the role of the scribble NO-GC2 complex for apical dendrite development [4] and also tend to think of NO-GC2 as an enzyme complex with a primary functional role in the central nervous system, a more general role of the NO-GC2 complex in the regulation of cell cell contact mediated growth inhibition seems an exciting possibility. In this context, we have been impressed by the tragic case of an only ten year old girl who died from the brain metastasis of a primary pulmonary adenocarcinoma [5]. Because of the unusual age of the patient and in an attempt to identify a targetable mutation, next generation sequencing was performed using a comprehensive cancer panel [5]. Due to the aforementioned work [1], this included an analysis of GUCY1A2 and in fact this gene was shown to be mutated along with only four other genes. In the current study, we asked the question whether the specific mutation present in the tumor of the ten year old girl interferes with NO-GC2 enzyme activity, and the ability of NO-GC2 to be targeted to cell–cell contacts after overexpression in immortalized cell lines.

**Methods:** Methods are described in detail in reference [3].

**Results:** Overexpression of the mutated enzyme complex led to a loss of cell–cell contact localization after overexpression in HEK293 cells (Figure 1). While we have clearly found evidence for an interaction of the NO-GC2 complex with the third PDZ domain of scribble, we still have have to check whether the mutation abrogates the interaction with this tumor suppressor. These results and other current results from our laboratory will be shown.

**Conclusions:** While it cannot be ruled out that somatic mutations in GUCY1A2 are a chance byproduct of genomic instability in tumors leading to passenger mutations rather than driver mutations in GUCY1A2, we suggest that GUCY1A2 deserves further attention as a potential relevant disease gene in tumors given the loss of the ability of the NO-GC2 complex to be targeted to cell–cell contacts in the presented unusual case.
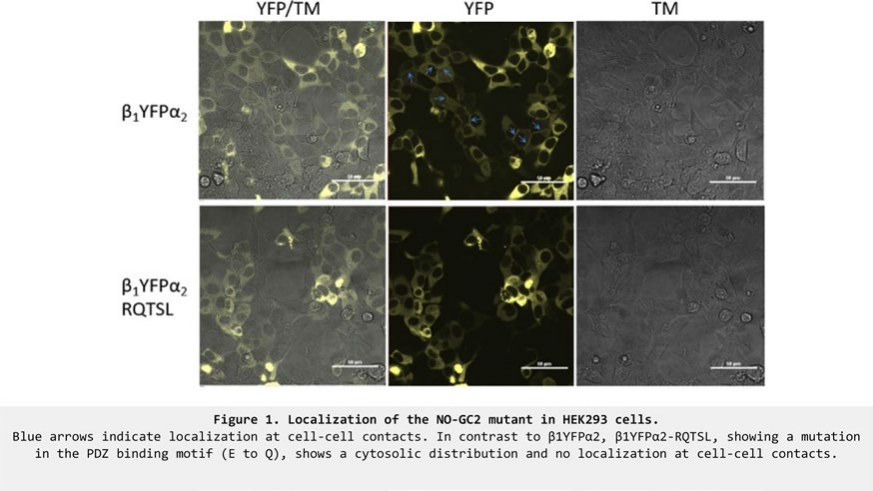


**References**

1. Sjöblom, T, Jones, S, Wood, LD, Parsons, DW, Lin, J, Barber, TD, Mandelker, D, Leary, RJ, Ptak, J, Silliman, N, Szabo, S, Buckhaults, P, Farrell, C, Meeh, P, Markowitz, SD, Willis, J, Dawson, D, Willson, JK, Gazdar, AF, Hartigan, J, Wu, L, Liu, C, Parmigiani, G, Park, BH, Bachman, KE, Papadopoulos, N, Vogelstein, B, Kinzler, KW, Velculescu, VE. 2006, ‘The consensus coding sequences of human breast and colorectal cancers’, *Science*, 314, 268–274
2. Rubin, AF, Green, P 2007, ‘Comment on “The consensus coding sequences of human breast and colorectal cancers”’, *Science*, 317, 500
3. Hochheiser, J, Haase, T, Busker, M, Sömmer, A, Kreienkamp, HJ, Behrends, S 2016, ‘Heterodimerization with the β_1_ subunit directs the α_2_ subunit of nitric oxide-sensitive guanylyl cyclase to calcium-insensitive cell–cell contacts in HEK293 cells: Interaction with Lin7a’, *Biochemical Pharmacology*, 122, 23–32
4. Szczurkowska, J, Guo, A, Martin, J, Lee, SI, Martinez, E, Chien, CT, Khan, TA, Singh, R, Dadson, D, Tran, TS, Pautot, S, Shelly, M 2022, ‘Semaphorin3A/PlexinA3 association with the scribble scaffold for cGMP increase is required for apical dendrite development’, *Cell Reports*, 38, 110483
5. De Martino, L, Errico, ME, Ruotolo, S, Cascone, D, Chiaravalli, S, Collini, P, Ferrari, A, Muto, P, Cinalli, G, Quaglietta, L 2018, ‘Pediatric lung adenocarcinoma presenting with brain metastasis: a case report’, *J Med Case Rep*, 12, 243

## O26 NO–GC–PRKG signaling in Marfan syndrome

### Andrea de la Fuente-Alonso^1,2^, Marta Toral^1,2^, Miguel R. Campanero^2,3^, Juan M. Redondo^1,2^

#### ^1^Centro Nacional de Investigaciones Cardiovasculares (CNIC), Gene regulation in cardiovascular remodeling and inflammation group, Madrid, Spain; ^2^Centro de Investigación Biomédica en Red de Enfermedades Cardiovasculares (CIBERCV), Madrid, Spain; ^3^Centro de Biología Molecular Severo Ochoa (CBMSO), Universidad Autónoma de Madrid, Consejo Superior de Investigaciones Científicas, Madrid, Spain

##### **Correspondence:** Andrea de la Fuente-Alonso (afuente@cnic.es), Juan M. Redondo (jmredondo@cnic.es) and Miguel R. Campanero (mcampanero@cbm.csic.es)

*J Transl Med* 2022, **21(1)**:O26

*Andrea de la Fuente-Alondo and Marta Toral contributed equally to this work.*

**Introduction:** Despite advances in the genetics of thoracic aortic aneurysm and dissection (TAAD), the molecular mechanisms underlying these diseases remain elusive. Unfortunately, current pharmacological treatments do not effectively retard aortic expansion or prevent catastrophic failure in these diseases, including Marfan syndrome (MFS).

**Methods:** MFS established disease was studied in a genetic MFS mouse model and NO-donors were administered to assess their capacity to induce aortopathy in wild-type mice. Primary cultures of vascular smooth muscle cells (VSMCs) were isolated from mouse aortas for in vitro studies. Additionally, blood and aortic tissue samples from healthy donors, Marfan patients, and mouse models were examined by high throughput proteomics analysis.

**Results:** We have reported that Nos2 is a critical mediator in the development of aneurysms whose expression is induced in MFS and have now further investigated how NOS2 contributes to TAAD. We now show that increased NOS2-derived NO levels stimulate the guanylate cyclase (sGC)–protein kinase G (PRKG) pathway in Marfan patients and mice, as evidenced by increased plasma cGMP and aortic staining of pVASP-S239. Our data also show that inhibitors of either sGC or PRKG, or lentiviral-mediated silencing of *Prkg1* in the aorta, regress the aortopathy of MFS mice. The levels of nitrated proteins are higher in plasma from MFS patients and mice, and in aortic tissue from Marfan mice than in control samples, indicating elevated circulating and tissue NO and suggesting that NO also mediates pathophysiological processes in a cGMP-independent fashion through mechanisms involving protein nitration.

**Conclusions:** These results show that the NO-sGC-PRKG signaling pathway mediates aortopathy in a mouse model of MFS and is activated in MFS mice and MFS patients. Our findings also identify potential therapeutic targets for intervention in human MFS as well as circulating biomarkers for monitoring and clinical follow up of MFS disease.

**Funding:** The project leading to these results has received funding from “La Caixa” Banking Foundation HR18-00068 and HR17-00247; Spanish Ministerio de Ciencia e Innovación grants RTI2018-099246-B-I00 (MICIU/AEI/FEDER, UE), SAF2015-636333R (MINECO/FEDER, UE), SAF2017- 88881R (MINECO/AEI/FEDER, UE), BIO2015-67580-P and PGC2018-097019-B-I00; Comunidad de Madrid through the European Social Fund (ESF)-financed program AORTASANA-CM (B2017/BMD-3676); Instituto de Salud Carlos III (CIBER-CV CB16/11/00264 and CB16/11/00277; PRB3- IPT17/0019-ProteoRed; and PI17/00381, with cofinancing from the European Regional Development Fund); Fundacio La Marato TV3 (20151330, and 122/C/2015); The Marfan Foundation USA Faculty grant 2017 MRF/1701.

## O27 Soluble guanylyl cyclase, in addition to cGMP production, mediates NO signaling by using thioredoxin as a nitrosothiol relay under oxidative stress

### Chuanlong Cui^1,2^, Changgong Wu^3^, Ping Shu^2^, Tong Liu^4^, Hong Li^4^, Annie Beuve^2^

#### ^1^Rutgers School of Graduate Studies, Newark Health Science, Newark, NJ, USA; ^2^Dept of Pharmacology, Physiology and Neuroscience, Rutgers, NJMS, Newark, NJ, USA; ^3^Thermo Fisher Scientific, Somerset, NJ, USA; ^4^Center for Advanced Proteomics Research, Rutgers, Newark, NJ, USA

##### **Correspondence:** Annie Beuve (beuveav@njms.rutgers.edu)

*J Transl Med* 2022, **21(1)**:O27

Soluble guanylyl cyclase (GC1) is an α/β heterodimer producing cGMP when stimulated by nitric oxide (NO). The NO-GC1-cGMP pathway is essential for cardiovascular homeostasis. Oxidative stress is known to disrupt the NO-GC1-cGMP pathway not only through endothelial dysfunction via reduction of NO availability, but also via oxidative reactions involving the thiols and heme of GC1, which make it unresponsive to NO stimulation. We discovered that under these conditions, GC1-α subunit increases cellular S-nitrosation (addition of a NO moiety to a Cysteine) via specific transfer of some of its nitrosothiols (SNO) to other proteins (transnitrosation). One of the SNO-targets identified by Mass Spectrometry (MS) was the oxidized form of Thioredoxin1 (oTrx1), which is unidirectionally transnitrosated by GC1 with αC610 as a SNO-donor and C73 of Trx1 as the SNO-recipient. Because oTrx1 itself drives transnitrosation, we sought and MS-identified SNO-proteins targeted by both GC1 and Trx1. Among the targets was the small GTPase RhoA. We found that transnitrosation of RhoA by SNO-GC1 requires oTrx1 as a nitrosothiol relay, suggesting a SNO-GC1 → oTrx1 → RhoA cascade. The RhoA signaling pathway is antagonized by the canonical NO-cGMP pathway and RhoA activity is also directly inhibited by S-nitrosation. In oxidized cells depleted for the GC1-α subunit, RhoA activity was significantly increased and SNO-RhoA levels decreased, suggesting that transnitrosation of RhoA by GC1 is an alternative way to inhibit RhoA signaling. We propose that as an adaptive response to oxidative stress, which desensitizes and S-nitrosates GC1, SNO-GC1 transnitrosation’s role is to rescue the disrupted function of the oxidized NO-GC1-cGMP pathway.

## Session 7 | Emerging Topics

## O28 Compartmentation of cGMP in Adipocytes

### Daniel Rowland^1,2^, Dennis de Coninck^1^, Alexander Pfeifer^1,2^

#### ^1^University Hospital Bonn, Institute of Experimental Pharmacology and Toxicology Bonn, Bonn North Rhine-Westphalia, Germany; ^2^Deutsche Forschungsgemeinschaft (DFG, German Research Foundation) - 214362475/GRK1873/2, Bonn, Germany

##### **Correspondence:** Daniel Rowland (drowland@uni-bonn.de)

*J Transl Med* 2022, **21(1)**:O28

**Introduction:** Obesity is characterized by an abnormal expansion in white adipose tissue (WAT) mass, the main energy storing organ of the body, which results in comorbidities such as metabolic syndrome, type 2 diabetes mellitus, cardiovascular disease, and certain types of cancer. Brown adipose tissue (BAT), on the other hand, is distinguished by its ability to dissipate energy in the form of heat through a process called non-shivering thermogenesis (NST), making it a promising target to combat obesity. Previous research has shown that cyclic nucleotides, such as cyclic adenosine monophosphate (cAMP) and cyclic guanosine monophosphate (cGMP) play a major role in BAT function. They enhance brown adipocyte (BA) differentiation, lipolysis, thermogenesis, mitochondrial biogenesis, energy expenditure and are able to induce browning of WAT. While the downstream effects of bulk cGMP on the differentiation of BA have been studied previously, differences between the function and characteristics of loco-regional pools of cGMP, derived from either the soluble guanylate cyclase (sGC) or the particulate GCs (natriuretic-peptide-receptor A and B; NPRA and NPRB), remain poorly understood.

**Methods:** Here, we utilize BA isolated from transgenic mice expressing the cytosolic cGMP Förster Resonance Energy Transfer biosensor cGi-500 [1, 2] to probe the dynamics of cGMP production and degradation by the GCs and phosphodiesterases (PDEs), respectively, in real-time.

**Results:** Our results demonstrate that, depending on which GC produces cGMP, different subsets of PDEs are involved in its degradation, thereby locally confining the distinct cGMP pools. Furthermore, we show that upon BA maturation, both the primary GC responsible for cGMP production as well as the distinct subsets of PDEs involved in its degradation change. Interestingly, we find that PDE1 not only exclusively degrades NPRB-cGMP, but also facilitates crosstalk between calcium and cGMP, thus providing a link for the modulatory effects of calcium on BA differentiation.

**Conclusions:** Loco-regional pools of cGMP associated with specific guanylate cyclases exist in BA and are confined by distinct subsets of PDEs. Upon BA maturation the characteristics of these cGMP pools are altered.

PDE1 is exclusively involved in degrading NPRB-cGMP and facilitates crosstalk between calcium and cGMP providing a possible explanation for the role of calcium in BA differentiation.

**Funding:** Funded by the Deutsche Forschungsgemeinschaft (DFG, German Research Foundation)—214362475/GRK 1873/2. We thank Prof. Robert Feil for generously providing us with the cGi-500 transgenic mice.

## O29 Uroguanylin activation of brown adipose tissue is age, gender, and phase of the estrous cycle dependent

### Nikola Habek^1,2^, Martina Ratko^1^, Milan Kordić^3^, Aleksandra Dugandžić^1,2^

#### ^1^School of Medicine, University of Zagreb, CIBR, Centre of Excellence for Basic, Clinical and Translational Neuroscience, Zagreb, Croatia; ^2^School of Medicine, University of Zagreb, Department of Physiology, Zagreb, Croatia; ^3^MKP Ltd., Zagreb, Croatia

##### **Correspondence:** Nikola Habek (nhabek@hiim.hr)

*J Transl Med* 2022, **21(1)**:O29

**Introduction:** Uroguanylin (UGN) leads to browning after prolonged i.c.v. application and it is released from the gut after a meal when activation of brown adipose tissue (BAT) occurs in a gender- and age-dependent manner^1,2^. In this study, we determined the acute activation of BAT by intranasal (i.n.) UGN application and possible mechanism of postprandial BAT activation via UGN.

**Methods:** In this study, male animals and female mice in estrus I and diestrus (DE) C57Bl/6NCrl were used. The activity of BAT was determined by infrared thermography (FLIR T-1020). The expression of UGN in the hypothalamus upon i.n. insulin or GLP-1 stimulation was determined by ELISA. The expression of GC-C was determined by immunohistochemistry. Membrane potential was measured by whole-cell patch-clamp and changes in intracellular ion concentrations by specific fluorescent dyes.

**Results:** In older animals, BAT activity is activated far more by a five-time smaller amount of UGN applied i.n. compared to i.p. This activation was smaller in female animals in DE and not present during E. Even though the depolarisation of POMC neurons by UGN was similar in all tested animal groups, GC-C expression in POMC and dopaminergic neurons in the Arcuate nucleus of the hypothalamus was different in male animals and female mice in DE and E which could explain differences in BAT activation due to estrous cycle. Also, UGN increased intracellular concentration of K^+^ and Ca^2+^ but not Cl^−^ in hypothalamic cells which is consistent with cell depolarization. GLP-1, applied i.n., changed BAT activity differently depending on age, gender, and estrous cycle, as shown for BAT application after a meal. Furthermore, i.n. application of GLP-1 changed pro-UGN expression in male but not female hypothalamus which was not accompanied by similar changes in blood, suggesting the existence of brain UGN and involvement of UGN in BAT activation via GLP-1.

**Conclusions:** Metabolic studies, especially ones investigating the regulation of BAT activity, should pay special attention to age, gender and phase of estrous cycle. The interplay between UGN, GLP-1, and insulin is especially important when the activity of BAT is measured in diabetic patients. Since BAT activation leads to glucose expenditure^2^, this and similar studies could lead to the development of new approaches involving the activation of BAT for the treatment of hyperglycaemia in diabetic patients, which will improve glucose metabolism and postpone insulin application.

**Funding:** This study is financed by the Croatian science foundation research grant (IP-2018-01-7416) and co-financed by the European Union through the European Regional Development Fund, Operational Programme Competitiveness and Cohesion, Grant Agreement No. KK.01.1.1.01.0007, CoRE-Neuro.

**References**

1. Habek, N, Dobrivojević Radmilović, M, Kordić, M, Ilić, K, Grgić, S, Farkaš, V, Bagarić, R, Škokić, S, Švarc, A, Dugandžić, A 2020, ‘Activation of brown adipose tissue in diet-induced thermogenesis is GC-C dependent’, *Pflugers Arch,* 472, 405–417.
2. Habek, N, Kordić, M, Jurenec, F, Dugandžić, A 2018, ‘Infrared thermography, a new method for detection brown adipose tissue activity after a meal in humans’, *Infrared Phys Technol,* 89, 271–276.

## O30 Therapeutic implications of nitroxyl (HNO) donors as a means of enhancing cGMP-mediated cardioprotection in the insulin-resistant diabetic myocardium

### Anida Velagic^1^, Jasmin C. Li^1^, Cheng Xue Qin^1^, Mandy Li^3,1^, Minh Deo^1^, Sarah Marshall^4^, Dovile Anderson^1^, Owen Woodman^1^, John Horowitz^2^, Barbara Kemp-Harper^3^, Rebecca H. Ritchie^1,3^

#### ^1^Monash University, Monash Institute of Pharmaceutical Sciences, Parkville, Australia; ^2^Adelaide University, Basil Hetzel Institute, Woodville, Australia; ^3^Monash University, Biomedicine Discovery Institute, Clayton, Australia; ^4^Monash University, Hudson Institute of Medical Research, Clayton, Australia

##### **Correspondence:** Rebecca H. Ritchie (rebecca.ritchie@monash.edu)

*J Transl Med* 2022, **21(1)**:O30

**Introduction:** Nitric oxide (NO) promotes vasodilation and cardiac relaxation, which are cGMP-dependent. Impaired responsiveness to NO in heart failure occurs largely due to dysregulated reactive oxygen species (ROS), which scavenge NO and oxidise its intracellular receptor soluble guanylate cyclase (sGC). HNO also promotes cGMP-dependent vasodilation and cardiac relaxation but lacks reactivity with ROS, remaining effective during oxidative stress. As diabetes is associated with oxidative stress, we determined whether the cGMP-mediated cardioprotective actions of HNO are preserved in the insulin-resistant diabetic rat myocardium ex vivo, and contrast these to an NO donor.

**Methods:** Male Sprague–Dawley rats commenced a high-fat diet (HFD) for 14wks, with low-dose streptozotocin (STZ, 2 × 35 mg/kg i.p). Dose-dependent responsiveness to the HNO donor Angeli’s salt and the NO donor diethylamine NONOate (DEA/NO, both 10^–10^ to 10^–5^ mol) were determined in isolated perfused hearts. Vascular responsiveness to the HNO donor Angeli’s salt as well as the NO donors sodium nitroprusside (SNP) and DEA/NO were determined in isolated mesenteric arteries (all 0.1 nM–50 µM). The role of sGC/cGMP was elucidated using 1*H*-[1,2,4]oxadiazolo[4,3-a]quinoxaline-1-one (ODQ; 10 µM).

**Results:** HFD + STZ induced type 2 diabetes in rats, confirmed on endpoint blood glucose (26.8 ± 1.0 vs 6.6 ± 0.2 mM*), HbA1c (5.3 ± 0.2% vs not detected*), impaired glucose tolerance, and plasma levels of insulin (1.2 ± 0.3 vs 0.5 ± 0.0 ng/ml*) and triglycerides (3.0 ± 0.7 vs 0.9 ± 0.2 mM*). Myocardial insulin responsiveness was blunted in diabetic rats, with blunted responsiveness in LV-dP/dt, LVEDP, coronary flow and hosphor-Akt. Oxidative stress was confirmed on cardiac oxidized glutathione (GSSG) levels. Both Angeli’s salt and DEA/NO elicited dose-dependent lusitropic (LV-dP/dt) and coronary vasodilator responses to DEA/NO. Whilst vasodilator responses to Angeli’s salt were preserved and the lusitropic responses were enhanced in diabetic rat hearts, responses to DEA/NO were impaired. Similarly, vasorelaxation to Angeli’s salt was augmented in diabetic mesenteric arteries, which were hyporesponsive to the relaxant effects of SNP and DEA/NO. Cardioprotective actions were mediated by sGC/cGMP, as demonstrated by clear shifts in the dose/response curve to the right with ODQ.

**Conclusions:** This is the first evidence that the cGMP-mediated lusitropic responses are preserved by the HNO donor Angeli’s salt in diabetes, and also circumvents NO resistance in the vasculature, highlighting the cardiovascular therapeutic potential of HNO donors as a means of enhancing cGMP-mediated cardioprotection, especially in acute heart failure.

## O31 Origin of NO-GC-positive activated fibroblasts and the role of NO-GC in angiotensin II-induced heart failure

### Lennart Kreutz^1^, Dieter Groneberg^1^, Alexander Pinto^2^, Michaela Kuhn^1^, Kai Schuh^1^, Andreas Friebe^1^

#### ^1^University of Würzburg, Institute of Physiology, Würzburg Bavaria, Germany; ^2^Baker Heart and Diabetes Institute, Cardiac Cellular Systems, Melbourne, Victoria, Australia

##### **Correspondence:** Lennart Kreutz (lennart.kreutz@stud-mail.uni-wuerzburg.de)

*J Transl Med* 2022, **21(1)**:O31

**Introduction:** NO-sensitive guanylyl cyclase (NO-GC) regulates many different physiological functions through generation of cGMP and is also involved in the pathophysiology of cardiac diseases. Vericiguat, a NO-GC stimulator, was shown to exert positive effects in heart failure patients by reducing hospitalizations and overall mortality (VICTORIA trial). However, little is known on the individual cardiac cell types that express NO-GC and the role of the enzyme in cardiac fibrosis and heart failure.

**Methods:** Using immunohistochemistry and single-cell RNA sequencing, we investigated the expression of NO-GC in the murine heart. We also performed tdTomato-based lineage tracing using Cre recombinase under the control of different promoters. With this system, we also generated a pericyte-specific NO-GC knockout (PDGFRß-GCKO). We used angiotensin II-releasing mini-pumps to induce cardiac hypertrophy. To determine the cardiac pump function, we carried out hemodynamic measurements including a dobutamine stress test.

**Results:** In the healthy myocardium, NO-GC is strongly expressed in pericytes and smooth muscle cells, but neither in endothelial cells nor fibroblasts. Angiotensin II-induced cardiac hypertrophy was paralleled by the development of fibrotic lesions, which were positive for NO-GC and PDGFRß immunosignals as well as PDGFRß-tdTomato-expressing cells. PDGFRß is a marker for pericytes, smooth muscle cells and some subtypes of fibroblasts. NO-GC-positive cells in the fibrotic areas were also positive for THBS4, a marker for activated fibroblasts. As neither pericytes nor smooth muscle cells migrate into the fibrotic sites we conclude that the cells in the fibrotic areas originate from PDGFRß-positive fibroblasts which acquire NO-GC under fibrotic conditions. Pericyte-specific deletion of NO-GC (PDGFRß-GCKO) led to elevated blood pressure determined with hemodynamic and tail-cuff measurements. After angiotensin II treatment, cardiac contractility and cardiac output were reduced in PDGFRß-GCKO mice, despite having the same degree of fibrosis as WT animals.

**Conclusions:** Taken together, our preliminary experiments indicate that NO-GC in cardiac pericytes plays a role in the regulation of contractility and fibrosis under pathophysiological conditions.

## O32 Angiotensin II-induced cardiac fibrosis is attenuated by myofibroblast-specific cGKI

### Melanie Cruz Santos^1^, Lena Birkenfeld^1^, Natalie Längst^1^, Anna Roslan^1^, Meinrad Gawaz^2^, Stefan Offermanns^3^, Fumito Ichinose^4,5^, Robert Lukowski^1^

#### ^1^University of Tübingen, Department of Pharmacology, Toxicology and Clinical Pharmacy, Institute of Pharmacy, Tübingen, Germany; ^2^University of Tübingen, Department of Cardiology and Angiology, University Hospital Tübingen, Tübingen, Germany; ^3^Max-Planck-Institute for Heart and Lung Research, Department of Pharmacology, Bad Nauheim, Germany; ^4^Massachusetts General Hospital, Department of Anesthesia, Critical Care and Pain Medicine, Boston, USA; ^5^Harvard Medical School, Boston, USA

##### **Correspondence:** Melanie Cruz Santos (melanie.cruz-santos@uni-tuebingen.de)

*J Transl Med* 2022, **21(1)**:O32

**Introduction:** The phenotype change of cardiac fibroblasts (CF) into cardiac myofibroblasts (CMF) is a critical event that accelerates the maladaptive remodeling response of the heart in response to Angiotensin II (Ang II). CMFs are characterized by enhanced production of extracellular matrix and matricellular proteins like periostin (*Postn*), thereby contributing to ventricular stiffness, pump dysfunction, and eventually to heart failure [1, 2]. Preclinical studies have attributed antifibrotic effects to cardiac NO/NP-cGMP-cGKI pathway activation, although the role of this cascade in CMF in vivo remains elusive so far [3–5].

**Methods:** The putative role of cGKI in CMF on Ang II-mediated cardiac remodeling was investigated using tamoxifen-induced CMF-specific conditional cGKI knockout (*Postn*Cre-cGKI^fl/fl^ *aka* cKO) and control (*Postn*Cre-cGKI^+/+^ *aka* CTR) mice. Cell-specific Cre-recombination was examined by employing a ROSA^Tg/+^-reporter mouse strain. For the induction of myocardial fibrosis (MF), osmotic minipumps releasing Ang II over 28 days were implanted. Cardiac hypertrophy, fibrosis, and cardiomyocyte (CM) cross sectional areas were evaluated in cardiac sections obtained from cKO and CTR mice. Cardiac function analysis was performed by echocardiography. Proliferative behaviors of CMF/CF were assessed by ki67 staining in remodeled hearts and in a grid-based in vitro assay with primary CMF/CF isolated from Ang II treated cKO and CTR.

**Results:** *Postn*Cre-based recombination system enabled specific detection of CMF in the ROSA^Tg/+^ reporter model and provided evidence for cGKI depletion in the fibrotic area of cKO hearts following Ang II treatment. Although CTR and cKO mice exhibited comparable levels of cardiac hypertrophy in response to prolonged Ang II infusion, MF and CM cross-sectional area were significantly increased in cKO. Consistent with this adverse remodeling phenotype, cKO mice revealed a significant structure-related distortion of global cardiac function and local muscle deformation in comparison to CTR mice. Accordingly, we observed a worsening of the ejection fraction, fractional shortening as well as reduced longitudinal peak strain and strain rate. In line with the in vivo results, CMF/CF derived from Ang II treated cKO mice displayed a significantly increased proliferation rate in comparison to cells isolated from CTR hearts.

**Conclusions:** Our data indicate that the cGMP/cGKI pathway in CMF attenuates Ang II-induced cardiac pro-fibrotic responses in vivo by reducing CMF proliferation rate. Our future aim is to evaluate whether the therapeutic potential of cGMP pathway activation would potentially ameliorate, or even reverse organ fibrosis induced by Ang II through cGMP/cGKI in CMF in vivo.

**Funding:** This project is funded by the Deutsche Forschungsgemeinschaft (DFG, German Research Foundation – Projektnummer 335549539/GRK2381). The authors thank F. Hofmann for providing cGKI^**fl/fl**^ mice.

**References**

1. Kurose, H. (2021). Cardiac Fibrosis and Fibroblasts. *Cells*, *10*(7), 1716. https://www.mdpi.com/2073-4409/10/7/1716
2. Sweeney, M., Corden, B., & Cook, S. A. (2020). Targeting cardiac fibrosis in heart failure with preserved ejection fraction: mirage or miracle? *EMBO Molecular Medicine*, *12*(10), e10865. https://doi.org/10.15252/emmm.201910865
3. Klaiber, M., Kruse, M., Völker, K., Schröter, J., Feil, R., Freichel, M., Gerling, A., Feil, S., Dietrich, A., Londoño, J. E. C., Baba, H. A., Abramowitz, J., Birnbaumer, L., Penninger, J. M., Pongs, O., & Kuhn, M. (2010). Novel insights into the mechanisms mediating the local antihypertrophic effects of cardiac atrial natriuretic peptide: role of cGMP-dependent protein kinase and RGS2. *Basic Research in Cardiology*, *105*(5), 583–595. https://doi.org/10.1007/s00395-010-0098-z
4. Frantz, S., Klaiber, M., Baba, H. A., Oberwinkler, H., Völker, K., Gaβner, B., Bayer, B., Abeβer, M., Schuh, K., Feil, R., Hofmann, F., & Kuhn, M. (2011). Stress-dependent dilated cardiomyopathy in mice with cardiomyocyte-restricted inactivation of cyclic GMP-dependent protein kinase I. *European Heart Journal*, *34*(16), 1233–1244. https://doi.org/10.1093/eurheartj/ehr445
5. Patrucco, E., Domes, K., Sbroggió, M., Blaich, A., Schlossmann, J., Desch, M., Rybalkin, S. D., Beavo, J. A., Lukowski, R., & Hofmann, F. (2014). Roles of cGMP-dependent protein kinase I (cGKI) and PDE5 in the regulation of Ang II-induced cardiac hypertrophy and fibrosis. *Proceedings of the National Academy of Sciences*, *111*(35), 12925–12929. https://doi.org/10.1073/pnas.1414364111

## O33 Natriuretic peptides increase cGMP around cardiomyocyte mitochondria and protect against apoptosis

### Gaia Calamera^1^, Bernadin D. Ndongson^1^, Dulasi Arunthavarajah^1^, Mette Ovesen^1^, Jeong Joo Kim^2^, Choel Kim^2,3^, Finn Olav Levy^1^, Kjetil W. Andressen^1^, Lise Román Moltzau^1^

#### ^1^University of Oslo and Oslo University Hospital, Department of Pharmacology, Institute of Clinical Medicine, Oslo, Norway; ^2^Baylor college of Medicine, Department of Pharmacology and Chemical Biology, Houston Texas, USA; ^3^Baylor college of Medicine, Verna and Marrs McLean Department of Biochemistry and Molecular Biology, Houston Texas, USA

##### **Correspondence:** Gaia Calamera (gaia.calamera@medisin.uio.no)

*J Transl Med* 2022, **21(1)**:O33

**Introduction:** The Natriuretic peptides ANP, BNP and CNP activate transmembrane guanylyl cyclases (GC) that produce cGMP. Natriuretic peptides have beneficial effects in the cardiovascular system and have previously been shown to regulate energy metabolism. However, little is known about the direct effect of natriuretic peptides in cardiac mitochondria and possible effects on cardiomyocyte apoptosis. Our aim is to determine whether natriuretic peptides increase cGMP in cardiomyocytes around mitochondria and whether this alters apoptosis.

**Methods:** Primary rat adult cardiomyocytes were cultured and apoptosis was evaluated by TUNEL staining and PARP cleavage. The involvement of the intrinsic pathway of apoptosis was determined by cytochrome c release and caspase 9 activation. To measure cGMP, we constructed a novel FRET-based biosensor and targeted this to the outer mitochondrial membrane (OMM). We measured phosphorylation of Drp1, and used Mitotracker and confocal microscopy to evaluate mitochondria elongation.

**Results:** Stimulating GC-A with ANP or GC-B with CNP reduced apoptosis and PARP cleavage, together with reduced caspase 9 activation and reduced cytochrome c release. This suggests that NPs decrease apoptosis through the intrinsic pathway that involves mitochondria. Moreover, we found that ANP and CNP could increase phosphorylation of the pro-apoptotic protein Drp1 and could induce mitochondria elongation, suggesting a protective effect. We have previously shown that GC-A-stimulation only produced modest cGMP increase using an untargeted cGMP biosensor. Here, we constructed a novel FRET-based biosensor selective for cGMP and targeted this biosensor to the OMM. Stimulation with either CNP or ANP increased cGMP locally around the mitochondria.

**Conclusions:** The natriuretic peptides ANP and CNP are protective against apoptosis. Our results suggests that cGMP targeting the mitochondrial outer membrane microdomain inhibits the pro-apoptotic protein Drp1, leading to mitochondrial elongation that inhibits apoptosis.

## Session 8 | Natriuretic Peptides and Receptors

## O34 Quantification of natriuretic peptides beyond post-translational modifications

### Jens P. Gøtze

#### Copenhagen University, Clinical Biochemistry, Copenhagen, Denmark

##### **Correspondence:** Jens P. Gøtze (jens.peter.goetze@regionh.dk)

*J Transl Med* 2022, **21(1)**:O34

Measurement of natriuretic peptides in plasma has become a key element in heart failure diagnostics. While measurement of the bioactive hormones was initially the biomarkers of choice, quantification of N-terminal fragments from their molecular precursors have gained clinical use, as the fragments are less prone to degradation and preanalytical pitfalls. The post-translational processing of the biosynthetic precursors, however, have proven elaborate with variable O-glycosylation and endoproteolytic cleavage, which introduces analytical bias. We have developed so-called processing-independent immunoassays (PIAs) for quantification of proANP, proBNP, and proCNP in plasma. This methodology allows for quantification of the total amount of secreted proforms irrespective of endoproteolysis and peptide modifications. In essence, a defined fragment is generated by trypsin cleavage prior to measurement, and the amount of measured fragment thus represents both the intact precursor and the endogenous fragments thereof. This methodology also allows for assessment of the degree of maturation, where the concentration of bioactive hormone can be related to the total amount of propeptide released.

## O35 C-type natriuretic peptide—cGMP signalling attenuates hyperproliferation of lung pericytes from patients with Pulmonary Hypertension

### Swati Dabral^1^, Minhee Noh^1^, Lisa Krebes^1^, Marco Abeßer^1^, Katharina Völker^1^, Tatiana Novoyatleva^2^, Ralph Schermuly^2^, Vinicio de Jesus Perez^3^, Michaela Kuhn^1^

#### ^1^University Würzburg, Institute of Physiology I, Würzburg Bavaria, Germany; ^2^Justus Liebig University of Giessen, German Center for Lung Research (DZL), Gießen, Germany; ^3^Stanford University, Divisions of Pulmonary and Critical Care Medicine and Stanford Cardiovascular Institute, Stanford California, USA

##### **Correspondence:** Swati Dabral (swati.dabral@uni-wuerzburg.de)

*J Transl Med* 2022, **21(1)**:O35

**Introduction:** Pulmonary Hypertension (PH) is caused by excessive pulmonary vascular contraction and remodelling, and leads to fatal right heart failure. A switch to a hyperproliferative and hypercontractile phenotype of pulmonary arterial smooth muscle cells (PASMCs) and microvascular pericytes in response to growth factors such as Platelet derived growth factor-BB (PDGF-BB) contributes to PH (1, 2). Endogenous factors opposing such alterations are barely known. C-type natriuretic peptide (CNP) is expressed in lung endothelium, but its local paracrine functions are unclear (3). Here we studied whether CNP, via Guanylyl Cyclase-B (GC-B) signalling, attenuates the pericyte dysfunction observed in PH and dissected the participating molecular mechanisms.

**Methods:** Please see below.

**Results:** Mice with systemic knockdown of the GC-B receptor (GC-B KD) were exposed to chronic hypoxia (21 days, 10% O_2_) to study the effect of GC-B downregulation on PH development. GC-B KD mice developed extremely severe PH under chronic hypoxia as evident from significantly increased right ventricular systolic pressure and hypertrophy as compared to controls. To delineate the vascular cells responsible for this phenotype, cGMP responses to CNP were compared between cultured human pulmonary microvascular endothelial cells, PASMCs and pericytes. While CNP barely increased cGMP production in endothelial cells, pericytes showed the highest cGMP responses. Interestingly, CNP attenuated PDGF-BB induced proliferation and migration of cultured pericytes from human control and PH specimens. Mechanistic studies revealed that CNP counter-regulates PDGF-BB-driven pro-proliferative molecular pathways in lung pericytes, unravelling a novel pathway of cGMP signalling.

**Conclusions:** Taken together our observations reveal that CNP prevents PDGF-BB induced lung pericyte proliferation and migration. The impairment of this effect might contribute to the exacerbated response of GC-B KD mice to hypoxia driven PH.

**Funding:** Supported by the Deutsche Forschungsgemeinschaft (DFG KU 1037/12-1 and DFG DA 2462/1-1).

**References**

1. Yuan K, et al. Increased Pyruvate Dehydrogenase Kinase 4 Expression in Lung Pericytes Is Associated with Reduced Endothelial-Pericyte Interactions and Small Vessel Loss in Pulmonary Arterial Hypertension. Am J Pathol. 2016;186:2500–14.
2. Dabral S, et al. A RASSF1A-HIF1α loop drives Warburg effect in cancer and pulmonary hypertension. Nat Commun. 2019 May 13; 10(1): 2130.
3. Kuhn M. Molecular Physiology of Membrane Guanylyl Cyclase Receptors. Physiol Rev. 2016;96:751–804.

## O36 Phosphoryl regulation of natriuretic peptide-stimulated guanylyl cyclases: from chemical discovery to physiologic phenotype

### Lincoln R. Potter

#### University of Minnesota, Biochemistry, Minneapolis Minnesota, USA

##### **Correspondence:** Lincoln R. Potter (potter@umn.edu)

*J Transl Med* 2022, **21(1)**:O36

**Introduction:** In 1992, guanylyl cyclase-A (GC-A), the receptor for atrial and B-type natriuretic peptides, was shown to be inactivated by dephosphorylation (1). Six years later, phosphorylation was shown to be essential for hormone-dependent activation of GC-A, and GC-B, a homologous receptor for C-type natriuretic peptide, was shown be regulated similarly (2, 3).

**Methods:** The GC-B^7E/7E^ mice were made by sequential homologous recombination. The GC-A^8E/8E^ mice were made using CRISPR-Cas9 technology.

**Results:** Over the past thirty years, the phosphorylation sites of GC-A and GC-B were identified, and glutamate-substituted-phosphomimetic mutants were engineered that are activated like the phosphorylated wild type receptors but cannot be inactivated by dephosphorylation. More recently, glutamates were substituted for the corresponding phosphorylated serine and threonine residues for both murine alleles of GC-A and GC-B to produce the GC-A^8E/8E^ and GC-B^7E/7E^ mice that exhibit elevated natriuretic peptide-stimulated guanylyl cyclase activity. Data from these genetic models has revealed how dephosphorylation of these sites allows unidentified pathways to inactivate GC-A and increase cardiac hypertrophy (4), as well as how luteinizing hormone and fibroblast growth factor-2 promote the dephosphorylation of GC-B to facilitate the resumption of meiosis in the oocyte and reductions in long bone length that results in dwarfism, respectively (5).

**Conclusions:** Dephosphorylation is a mechanism that allows opposing signaling pathways to inactivate GC-A and GC-B and inhibit natriuretic peptide-stimulated physiologic responses.

**Acknowledgements:** I thank Brandon Wagner, Jerid Robinson, Deborah Dickey, Tim O'Connell, John Osborn, Laurence Legeai-Mallet, Leia Shuhaibar, Siu-Pok Yee, Jeremy Egbert and Laurinda Jaffe for invaluable contributions to our research on phosphorylation-dependent regulation of receptor guanylyl cyclases that was described in this report.

**References**

1. Potter, L. R., and Garbers, D. L. (1992) Dephosphorylation of the guanylyl cyclase-A receptor causes desensitization. *The Journal of biological chemistry* **267**, 14531–14534
2. Potter, L. R., and Hunter, T. (1998) Phosphorylation of the kinase homology domain is essential for activation of the A-type natriuretic peptide receptor. *Molecular and cellular biology* **18**, 2164–2172
3. Potter, L. R. (1998) Phosphorylation-dependent regulation of the guanylyl cyclase-linked natriuretic peptide receptor B: dephosphorylation is a mechanism of desensitization. *Biochemistry37*, 2422–2429
4. Wagner, B. M., Robinson, J. W., Healy, C. L., Gauthier, M., Dickey, D. M., Yee, S. P., Osborn, J. W., O'Connell, T. D., and Potter, L. R. (2022) Guanylyl cyclase-A phosphorylation decreases cardiac hypertrophy and improves systolic function in male, but not female, mice. *FASEB J* **36**, e22069
5. Wagner, B. M., Robinson, J. W., Lin, Y. W., Lee, Y. C., Kaci, N., Legeai-Mallet, L., and Potter, L. R. (2021) Prevention of guanylyl cyclase-B dephosphorylation rescues achondroplastic dwarfism. *JCI Insight* **6,** 1–14.

## O37 C-type natriuretic peptide regulates metabolic homeostasis

### Cristina Perez-Ternero^1^, Aisah Aubdool^1^, Raj Makwana^2^, Gareth Sanger^2^, Roland Stimson^3^, Li Chan^1^, Amie Moyes^1^, Adrian Hobbs^1^

#### ^1^Queen Mary University of London, William Harvey Research Institute, London, UK; ^2^Queen Mary University of London, Blizard Institute and the National Centre for Bowel Research, London, UK; ^3^University of Edinburgh, The Queen’s Medical Research Institute, Edinburgh, UK

##### **Correspondence:** Cristina Perez-Ternero (c.perez-ternero@qmul.ac.uk)

*J Transl Med* 2022, **21(1)**:O37

**Introduction:** The human and economic costs of obesity remain unacceptably high despite extensive efforts to decipher the molecular mechanisms underpinning energy metabolism. C-type natriuretic peptide (CNP) plays a key role in the cardiovascular system, regulating blood flow, angiogenesis, cardiac function, and immune cell reactivity, but a definitive role for CNP in the regulation of energy homeostasis remains to be established. Herein, we use a novel transgenic mouse model with global inducible CNP deletion and in vitro analyses in primary adipocytes to elucidate the role of CNP in energy metabolism.

**Methods:** In vivo analyses of body weight, fat composition, metabolic phenotyping at or below thermoneutrality, and glucose sensitivity were explored in wild type (WT), global inducible CNP knockout (gbCNP^−/−^) mice and global constitutive natriuretic peptide receptor (NPR)-C nullanimals (NPR-C^−/−^) fed standard or high-fat diet for 6 weeks. Administration of CNP or the NPR-C agonist cANF^4−23^ via subcutaneous mini-pumps was used to explore the effects of receptor-specific CNP signalling. Primary adipocytes were isolated from the stromal vascular fraction of WT, gbCNP^−/−^ and NPR-C^−/−^ and used to understand the pathways involved in CNP signalling affecting energy metabolism.

**Results:** Global CNPknockout mice and NPR-C^−/−^ animals were characterised by reduced body weight and lower white adipose tissue accumulation regardless of the fat content of the diet. gbCNP^−/−^ animals exhibited higher body temperature and presented increased browning of white adipose tissue and upregulation of thermogenic markers (UCP-1 and PGC-1α) in the white and brown adipose tissue*. *In vitro studies in adipocytes revealed that forskolin-induced cAMP formation was attenuated by CNP, an effect that was lost in NPR-C^−/−^ cells. The anti-thermogenic effect of CNP was confirmed to be NPR-C-dependent following a reduction in body temperature in mice that received cANF^4−23^.In vitro cultured adipocytes from gbCNP^−/−^ mice accumulated less triglycerides, while the addition of exogenous CNP to WT adipocytes potentiated the lipid accumulation and the expression of adipogenic markers (PPAR-γ and adiponectin). This adipogenic effect of CNP was further explored by administration of CNP via subcutaneous mini-pump to NPR-C^−/−^ mice, which gained more weight than control mice. NPR-B silencing in vitro in adipocytes resulted in reduced triglyceride accumulation, confirming the role of CNP in adipogenesis.

**Conclusions:** In sum, these data reveal a new mechanism for CNP in energy metabolism, whereby the peptide reduces thermogenesis via NPR-C and stimulates adipogenesis via NPR-B (Figure 1).
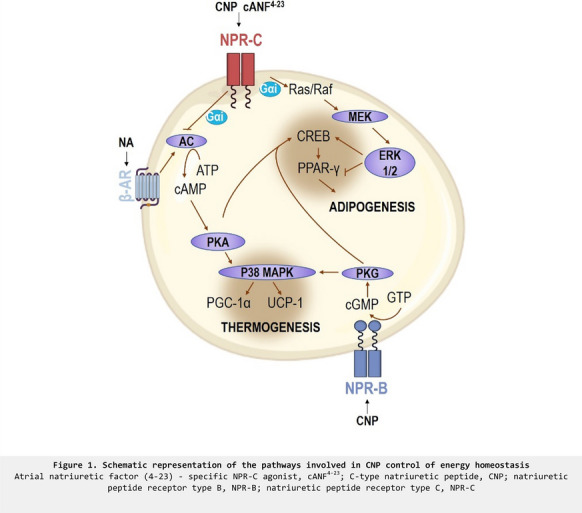


**Funding:** Funded by a British Heart Foundation Programme Grant.

## Session 9 | Technological Advances & New Applications

## O38 What happens in the cilia stays in the cilia, or localized cGMP fluxes in *C. elegans* sensory neurons

### Noelle L'Etoile

#### University of California, Department of Cell & Tissue Biology, San Fransico, USA

##### **Correspondence:** Noelle L'Etoile (noelle.letoile@ucsf.edu)

*J Transl Med* 2022, **21(1)**:O38

**Introduction:** cGMP is an important second massager in *C. elegans* sensory signal transduction. This is evidenced by the proliferation of receptor guanylyl cyclases: twenty seven are expressed in worms. Intriguingly, there is evidence that they can form both hetero and homo-dimers. The dimers would allow for a diversity of ligand binding. This may allow this animal that relies on chemosensory cues to better navigate its world. Navigation requires course correction over both short and long time scales. Rapid adaptation permits acute travel up a chemical gradient and longer adaptation can desensitize the response to an unprofitable cue for hours. We and others in the field of C. elegans sensory biology have found that cGMP is required across time scales.

**Methods:** In the sensory cilia of the olfactory neuron AWC, specific cGMP producing receptor GCs are localized sometimes in discreet domains. cGMP gated channels localized in these cilia along with the GCs allow transduction of odor signals by opening in response to changes in odor concentration. The cGMP-dependent protein kinase PKG-1/EGL-4 adapts the channels response at short time scales of tens of minutes. Odor signals that persist for an hour or more can direct the PKG to enter the nucleus to change chromatin and gene expression. Adaptation to one odor signal does not affect sensation of another and this is hard to understand if the kinase enters the nucleus where it may affect gene expression but the mechanism by which this is odor specific is unclear.

**Results:** We thought that we might get a clue as to how the odor specificity of adaptation is achieved by watching how the signal the signal is processed at each location in the cell. Thus, we adapted the FLINCG sensory from Bhargava et al. 2013 and we used this to look at cGMP levels in sensory neurons. We looked first at the salt sensing neurons as the reporter was most reliable in these. We found that when we stimulated the salt sensitive neurons (ASER/L) the cGMP flux in the cell body of was the opposite of the calcium flux in the same location. This is similar to what was reported in the olfactory AWC neuron. When we looked more closely at the cilia, we were intrigued to find that the sensory cilia where salt is perceived and adapted to rapidly showed that cGMP and calcium fluxes rose and fell in the same direction. The sign of the change in cGMP was inverted once it left the cilia. This too is similar to what was reported in the olfactory AWC neuron.

**Conclusions:** What happens in the cilia, stays in the cilia. That way the sensory compartment may be able to adapt on its own timescale with rapid on rapid of adaptation leaving the sensory unit responsive while the nucleus can act like its own independent unit and adapt more slowly and integrate over longer time scales.

## O39 Controlling cGMP downstream signaling with cellular and subcellular specificity using SponGee

### Oriol Ros^1^, Sarah Baudet^1^, Yvrick Zagar^1^, Fiona Roche^1^, Sandrine Couvet^1^, Alain Aghaie^3^, Yves Mechulam^2^, Xavier Nicol^1,4^

#### ^1^Sorbonne University, Vision Institute, Paris, France; ^2^Ecole Polytechnique, Laboratoire de Biochimie, Palaiseau, France; ^3^Institut Pasteur, UMR_S 1120, Paris, France; ^4^CNRS, UMR 7210, Paris, France

##### **Correspondence:** Xavier Nicol (xavier.nicol@inserm.fr)

*J Transl Med* 2022, **21(1)**:O39

**Introduction:** cGMP is a signaling molecule shared by many pathways. The highly controlled compartmentation of cGMP signals contributes to the specific activation of each downstream pathway and cellular process downstream of this cyclic nucleotide. However most cGMP manipulating reagents lack subcellular specificity. We developed SponGee (Sponge inhibiting cGMP signaling), a genetically-encoded cGMP scavenger that enables to prevent the modulation of cGMP downstream signaling and provides cellular and subcellular specificity to such a manipulation.

**Methods:** The affinity of SponGee for cGMP was evaluated using fluorescence anisotropy measurements of Fluo-cGMP followed by a competitive assay with unmodified cGMP. cGMP variations in living cells was monitored using the ^T^hPDE5^VV^ FRET biosensor, a variant of cGES-DE5 in which the donor and acceptor fluorescent proteins have been replaced by mTurquoise and a tandem of mVenus respectively.

**Results:** SponGee was engineered as a PKG1α and PKG1β chimera [1] that lacks the catalytic domain of these enzymes. The dissociation constant of SponGee for cGMP is 0.97 ± 0.26 µM. This cGMP buffer prevents pharmacologically-induced elevation in cGMP concentration detected by a FRET biosensor in living cells, highlighting the reduced availability of free cGMP for its downstream effectors when SponGee is expressed. This observation was confirmed by the reduction of activated cGMP downstream effectors in SponGee-expressing cells. SponGee expression was sufficient to prevent the increase in cGMP concentration, even in the presence of high cAMP concentration, highlighting its specificity for cGMP over cAMP. The electroporation of SponGee in developing cortical neurons alter their migration pattern confirming that SponGee is compatible with in vivo experimentation. To demonstrate the ability of SponGee to control cGMP concentration in chosen subcellular domains, SponGee was fused to targeting sequences restricting its expression either in the vicinity lipid rafts (Lyn-SponGee) or at the plasma membrane but outside lipid rafts (SponGee-Kras). Using this approaches we exemplify the ability of SponGee to buffer cGMP in subcellular compartments by investigating the cGMP signaling involved in the pathfinding of developing retinal axons.

**Conclusions:** SponGee is a genetically-encoded cGMP scavenger that enables to prevent the activation of downstream signaling with cellular and subcellular specificity [2].

**Acknowledgements:** We are grateful to the members of our labs and of J. Livet lab for thoughtful discussion and to K. Jalink for the generous gift of H147. This work was supported by grants from Agence Nationale de la Recherche (ANR-15-CE16-0007-01), Retina France, and Sorbonne Université (FCS-SU IDEX SUPER SU-15-R-PERSU-17) to X.N. This work was performed in the frame of the LABEX LIFESENSES (ANR-10-LABX-65) and IHU FOReSIGHT (ANR-18-IAHU-0001), supported by French state funds managed by the Agence Nationale de la Recherche within the Investissements d’Avenir program. S.B. was supported by a fellowship from the ED3C doctoral program (Sorbonne Université).

**Disclosure:** A patent describing SponGee and its applications is pending.

**References**

1. Ruth P, Pfeifer A, Kamm S, Klatt P, Dostmann WR, Hofmann F. Identification of the amino acid sequences responsible for high affinity activation of cGMP kinase Ialpha. J Biol Chem. 1997;272:10522–8.
2. Ros O, Zagar Y, Ribes S, Baudet S, Loulier K, Couvet S, et al. SponGee: A Genetic Tool for Subcellular and Cell-Specific cGMP Manipulation. Cell Rep. 2019;27:4003–4012.e6.

## O40 The sGC activator inhale mosliciguat (BAY 1237592): a new chapter in pulmonary hypertension therapy via activation of Apo-sGC

### Eva-Maria Becker-Pelster

#### Bayer AG Pharmaceuticals, Cardiovascular Research, Wuppertal North Rhine-Westphalia, Germany

##### **Correspondence:** Eva-Maria Becker-Pelster (eva.becker-pelster@bayer.com)

*J Transl Med* 2022, **21(1)**:O40

*Eva M. Becker-Pelster* ^***1***^ *is presenting on behalf of the entire research and development teams working on BAY123 (mosliciguat) in recent years, especially Michael G. Hahn* ^***1***^ *, Martina Delbeck* ^***1***^ *, Lisa Dietz* ^***1***^ *, Jörg Hüser* ^***1***^ *[EB1], Johannes Kopf* ^***1***^ *, Thomas Kraemer* ^***1***^ *, Tobias Marquardt* ^***1***^ *, Thomas Mondritzki* ^***1,2***^ *, Johannes Nagelschmitz* ^***1***^ *, Sylvia M. Nikkho* ^***1***^ *, Philippe V. Pires* ^***1***^ *, Hanna Tinel* ^***1***^ *, Gerrit Weimann* ^***1***^ *, Frank Wunder* ^***1***^ *, Peter Sandner* ^***1,3***^ *, Joachim Schuhmacher* ^***1***^ *, Johannes-Peter Stasch* ^***1,4***^ *, and Hubert K.F. Truebel* ^***1***^ *Bayer AG, Pharmaceuticals R&D, Pharma Research Center, Wuppertal, Germany,* ^***2***^ *Fakultät für Gesundheit, University Witten/Herdecke, Witten, Germany,* ^***3***^ *Department of Pharmacology, Hannover Medical School, Hannover, Germany,* ^***4***^ *Institute of Pharmacy, University Halle-Wittenberg, Halle, Germany.*

**Introduction:** Oxidative stress is associated with many cardiopulmonary diseases and leads to impairment in the nitric oxide (NO)/soluble guanylate cyclase (sGC) signaling pathway, shifting the balance from native sGC towards heme-free apo-sGC. This shift of the redox equilibrium is associated with unresponsiveness towards iNO and sGC stimulators. Therefore, it was our goal to develop a new high potent and selective inhaled sGC activator to specifically and lung-selectively target apo sGC- and to investigate its therapeutic potential in pulmonary hypertension (PH).

**Methods:** Here we report the discovery and in vitro and in vivo characterization of the sGC activator mosliciguat (BAY 1237592).

**Results:** Mosliciguat activates apo-sGC leading to improved cardiopulmonary circulation. Importantly, lung-selective effects, as reduced pulmonary artery pressure without reduced systemic blood pressure, were seen after inhaled application in a thromboxane induced pulmonary arterial hypertension minipig model. These effects were observed over a broad dose range with a long duration of action and were further enhanced under experimental oxidative stress conditions. In a unilateral broncho-occlusion minipig model, inhaled mosliciguat decreased pulmonary arterial pressure without deterioration in ventilation/perfusion match. With respect to airway resistance, mosliciguat showed additional beneficial bronchodilatory effects in an acetylcholine induced rat model. In summary, the inhaled sGC activator mosliciguat may overcome treatment limitations in patients with pulmonary hypertension by improving pulmonary circulation and airway resistance without systemic overspill or ventilation/perfusion mismatch.

**Conclusions:** Thus, mosliciguat could become a new therapeutic paradigm, exhibiting a unique mode of action and route of application, and is currently under clinical development in phase 1b for pulmonary hypertension.

## POSTER PRESENTATIONS

## P1 Small molecules enhance natriuretic peptide activity through a novel allosteric site on the natriuretic peptide receptor guanylyl cyclase-A

### Henriette Andresen^1,2^, Christina Pérez-Ternero^3^, Jerid Robinson^4^, Deborah Dickey^4^, Adrian Hobbs^3^, Lincoln R. Potter^4^, Finn Olav Levy^1^, Alessandro Cataliotti^2^, Lise Román Moltzau^1^

#### ^1^University of Oslo and Oslo University Hospital, Department of Pharmacology, Institute of Clinical Medicine, Oslo, Norway; ^2^University of Oslo and Oslo University Hospital, Institute of Experimental Medical Research, Oslo, Norway; ^3^Queen Mary University of London, William Harvey Research Institute, Barts & London School of Medicine, London, UK; ^4^University of Minnesota Medical School, Department of Biochemistry, Molecular Biology and Biophysics, Minneapolis, USA

##### **Correspondence:** Henriette Andresen (henriette.andresen@medisin.uio.no)

*J Transl Med* 2022, **21(1)**:P1

**Introduction:** Activation of natriuretic peptide receptor A (NPR-A; GC-A) by endogenous atrial natriuretic peptide (ANP) and B-type natriuretic peptide (BNP) is associated with several beneficial cardiovascular and renal effects. Recombinant BNP peptides have shown such effects. However, they have poor bioavailability and short half-life. In contrast to peptides, our novel approach is to use small molecular drugs to activate NPR‐A.

**Methods:** Cyclic GMP measurements were performed in QBI HEK293 cells expressing NPR-A, NPR-B or chimerae of the two receptors using Alpha Screen technology. Binding assays were performed in membrane preparations or whole cells using ^125^I-ANP. Vasorelaxation was measured in aortic rings isolated from Wistar rats.

**Results:** We have identified small molecular allosteric enhancers of NPR-A, which increase the efficacy and potency of BNP and ANP in their ability to activate NPR‐A, in various in vitro and ex vivo systems. These small molecular drugs are selective to NPR-A and one unique amino acid residue in NPR-A is crucial for their activity.

**Conclusions:** We describe novel allosteric enhancers of NPR-A that do not mediate their actions through previously described allosteric binding sites or via known mechanisms of action.

## P2 The sGC activator BAY 54-6544 improves vascular function and survival in an accelerated aging model

### Ehsan Ataei^1^, Keivan Golshiri^1^, Ingrid van der Pluijm^1^, Jan Danser^1^, Peter Sandner^2^, Anton Roks^1^

#### ^1^Erasmus Medical Center, Depts. of Internal Medicine, Radiology and Surgery, Rotterdam, Netherlands; ^2^Bayer AG, Leverkusen, Germany

##### **Correspondence:** Ehsan Ataei (e.ataeiataabadi@erasmusmc.nl)

*J Transl Med* 2022, **21(1)**:P2

**Introduction:** Vascular ageing is a key driver of cardiovascular diseases. Mice with partial deletion of the repair enzyme gene *Ercc1* (*Ercc1*^*∆/−*^) display accelerated vascular ageing, die prematurely between the age of 14 and 26 weeks, and can be used to test interventions in ageing. Vascular ageing in humans and in *Ercc1*^*∆/−*^ is associated with decreased nitric oxide-soluble guanylate cyclase-cyclic guanosine monophosphate (NO-sGC-cGMP) signaling, oxidative stress, inflammation, and cellular senescence. Restoring this pathway could be an effective treatment opportunity for vascular ageing. We therefore, investigated the therapeutic potential of the sGC activator BAY 56-6544 in *Ercc1*^*∆/−*^mice.

**Methods:** *Ercc1*^*∆/−*^and wild-type (WT) mice received either vehicle or BAY 54-6544 chow for 8 weeks. Blood pressure (BP) and in-vivo vasodilation (reactive hyperemia: RH) were measured at 15 and 16 weeks respectively. Mice were sacrificed at 17 weeks. Thoracic aorta (TA) endothelium-dependent (ED) and -independent (EI) relaxations were assessed (wire-myograph). Afterwards, we investigated the expression level of senescence markers *p16* and *p21* and inflammatory markers *Il-6* and *Ccl2* in aorta by qPCR.

**Results:** BP was similar in all groups. RH was decreased in *Ercc1*^*∆/−*^ vs. WT (P = 0.03) and was restored by BAY 54-6544 treatment to level of WT. In vehicle-treated *Ercc1*^*∆/−*^ mice maximal NO-mediated relaxation in isolated TA to acetylcholine (ED) and sodium nitroprusside (EI) were decreased as compared to WT (ED: 46.97 ± 7.04 vs. 72.33 ± 3.03, P = 0.0001; EI: 75.47 ± 5.27 vs. 88.30 ± 3.64, P = 0.02). Although BAY 54-6544 treatment did not improve ED response significantly, it improved EI relaxation in *Ercc1*^*∆/−*^ to the same level as in WT (EI 88.54 ± 3.26). BAY 54-6544 did not change responses in WT. The mRNA expression of *p16*, *p21* and *Ccl2* were significantly higher in *Ercc1*^*∆/−*^, and was attenuated by BAY 54-6544. BAY 54-76544 also improved survival of *Ercc1*^*∆/−*^.

**Conclusions:** DNA repair deficiency in *Ercc1*^*∆/−*^ mice causes a disruption of NO-sGC-cGMP signaling and a decrease of vasodilator response. Treatment with the BAY 54-6544 improved NO-mediated vasodilation and attenuated vascular senescence and inflammation markers. Thus, BAY 54-6544 could be a potential therapeutic treatment to attenuate vascular ageing.

**Acknowledgements:** We thank Bayer AG for in kind support, and Stichting Lijf en Leven for financial contribution.

## P3 The cGMP signaling pathway in breast cancer cell lines

### Mariagiovanna Barresi, Malte Roessing, Rahel Menges, Daniel Stehle, Robert Feil

#### University of Tübingen, Interfaculty Institute of Biochemistry, Tübingen Baden-Württemberg, Germany

##### **Correspondence:** Mariagiovanna Barresi (mariagiovanna.barresi@uni-tuebingen.de)

*J Transl Med* 2022, **21(1)**:P3

**Introduction:** Breast cancer is the most common cancer in women. Although new diagnostic tools and therapeutic strategies led to a significant reduction in breast cancer related mortality, more efficacious and less toxic drugs are needed. Recent studies suggested that the cGMP signaling pathway may be aberrantly regulated in breast cancer [1, 2], but cGMP’s functional relevance during tumorigenesis is not well understood. The aim of the present study was to improve our understanding of cGMP signaling during breast cancer development and progression.

**Methods:** The expression of cGMP pathway proteins in the human breast cancer cell lines Hs578T, MDA-MB-157 and BT549 as well as in the murine 4T1 breast cancer cell line was analyzed by Western blot analysis. Furthermore, we used the Förster resonance energy transfer (FRET)-based cGMP biosensor cGi500 [3] to monitor cGMP concentration changes in the tumor cell lines upon application of atrial natriuretic peptide (ANP), C-type natriuretic peptide (CNP), the NO donor DEA-NO, or the NO-sensitive guanylyl cyclase (NO‑GC) stimulator Riociguat. Finally, we used a wound healing assay to analyze how tumor-conditioned medium (TCM) from breast cancer cells affected the migration of vascular smooth muscle cells (VSMCs).

**Results:** The different breast cancer cell lines showed a heterogenous pattern of cGMP signaling pathway activity. Whereas none of the tested breast cancer cell lines generated cGMP in response to ANP, some (Hs578T, 4T1) showed strong increases of the intracellular cGMP levels after CNP stimulation. NO-induced cGMP generation was detected in Hs578T, MDA-MB-157 and BT549 cells, and these signals were potentiated by the NO‑GC stimulator Riociguat. Expression of NO‑GC in these cell lines could be validated by Western blot analysis. Interestingly, VSMCs treated with TCM from 4T1 breast cancer cells that have been stimulated with 8-Br-cGMP showed a decreased migration as compared to VSMCs treated with TCM from unstimulated 4T1 cells.

**Conclusions:** Here, we showed that different cGMP signaling pathway are functionally expressed in various breast cancer cell lines. Our results suggest that cGMP signaling in breast cancer cells decreases the migratory potential of VSMCs in the tumor stroma via a yet unknown paracrine factor, thereby, regulating the tumor vasculature. In the future, it must be tested in in vivo models, if the newly discovered cGMP-dependent interaction between breast cancer cells and VSMCs provides a novel target to improve the efficacy of breast cancer therapies.

**Acknowledgements:** We thank the Deutsche Forschungsgemeinschaft for the financial support of the Research Training Group “cGMP: From Bedside to Bench” (grant number 335549539/GRK 2381); Prof. A. Friebe for the NO-GC antibody; Prof. L. A. Jaffe for the PDE5 antibody; and Prof. S. Brucker, Dr. A. Koch, and Prof. H. Brauch for the human breast cancer cell lines.

**References**

1. Fajardo, A. M., Piazza, G. A., & Tinsley, H. N., 2014, The role of cyclic nucleotide signaling pathways in cancer: targets for prevention and treatment, Cancers (Basel), 6(1), 436–458, https://doi.org/10.3390/cancers6010436
2. Lv, Y., Wang, X., Li, X., Xu, G., Bai, Y., Wu, J., Piao, Y., Shi, Y., Xiang, R., & Wang, L., 2020, Nucleotide de novo synthesis increases breast cancer stemness and metastasis via cGMP-PKG-MAPK signaling pathway. PLoS biology, 18(11), e3000872, https://doi.org/10.1371/journal.pbio.3000872
3. Russwurm, M., Mullershausen, F., Friebe, A., Jäger, R., Russwurm, C., & Koesling, D., 2007, Design of fluorescence resonance energy transfer (FRET)-based cGMP indicators: a systematic approach, Biochemical Journal, 407(1), 69–77, https://doi.org/10.1042/BJ20070348

## P4 Guanylyl cyclase-A, not natriuretic peptide receptor C, mediates the endothelial actions of ANP regulating arterial blood pressure and angiogenesis

### Rebecca Bosch^1^, Franziska Werner^1^, Tamara Potapenko^1^, Lisa Krebes^1^, Katharina Völker^1^, Berin Upcin^2^, Süleyman Ergün^2^, Fubiao Shi^3^, Sheila Collins^3^, Michaela Kuhn^1^

#### ^1^University of Würzburg, Institute of Physiology I, Würzburg Bavaria, Germany; ^2^University of Würzburg, Institute of Anatomy and Cell Biology, Würzburg Bavaria, Germany; ^3^Vanderbilt University Medical Center, Division of Cardiovascular Medicine, Nashville Tennessee, USA

##### **Correspondence:** Rebecca Bosch (rebecca.bosch@uni-wuerzburg.de)

*J Transl Med* 2022, **21(1)**:P4

**Introduction:** Endothelial effects of cardiac ANP are critically involved in the regulation of arterial blood pressure (ABP) and intravascular volume. These effects are mediated by the guanylyl cyclase-A (GC-A) receptor and cyclic GMP [1]. NPs also bind with high affinity to the NP receptor C (NPR-C), which lacks a cyclase domain. Its short intracellular region contains G_i/o_ coupling sequences. Studies showed that NPR-C internalizes and clears NPs, while others revealed G-protein signalling [2]. Besides regulating ABP, ANP improves angiogenesis. Results in cultured endothelial cells indicated that NPR-C participates in this effect [3]. Here we studied human cardiac microvascular endothelial cells (hCMEC) and genetic mouse models to differentiate the role of endothelial GC-A versus NPR-C in the regulation of ABP and angiogenesis.

**Methods:** Please see below.

**Results: **In vitro: ANP enhanced cGMP levels, ERK1/2 phosphorylation and proliferation of hCMEC. Such effects were significantly reduced by Rp-8-Br-PET-cGMPs, an inhibitor of cGMP-dependent protein kinase I. Intriguingly they were mimicked by 8-Br-cGMP, a cGMP analog, and by cANP-4-23, a NPR-C ligand, suggesting that GC-A and NPR-C participate in the effects of ANP on endothelial proliferation. To study the relevance of these findings in native cells, we generated mice with endothelial (EC)-restricted deletions (KO) of either receptor using floxed *Npr1* or floxed *Npr3* mice interbred with *Tie2-Cre* [1, 4]. Ex vivo: ANP enhanced the sprouting of capillaries from cultured aortic rings prepared from *floxed* control mice. These effects were reduced in aortae from EC GC-A KO but preserved in aortae from EC NPR-C KO mice. In vivo: Non-invasive recordings showed elevated ABP in EC GC-A KO but unaltered ABP in EC NPR-C KO mice. To study the role of endothelial GC-A/cGMP versus NPR-C signalling in the effects of NPs on reparative neoangiogenesis, we subjected mice to experimental hind limb ischemia. Non-invasive laser Doppler perfusion imaging showed significant impairment of post-ischemic blood flow recovery during the phase of neoangiogenesis in EC GC-A KO but not in EC NPR-C KO mice. Histology confirmed that muscular capillary densities in the ischemic legs were reduced in EC GC-A KO but EC NPR-C KO mice in comparison to respective control littermates.

**Conclusions:** These data emphasize the central (patho)physiological role of the endothelial actions of ANP in the regulation of ABP and regenerative angiogenesis. They support the concept of pharmacological activation of GC-A as an innovative approach to treat arterial hypertension, peripheral artery disease and ischemic cardiovascular disorders.

**Funding:** This study is supported by the Deutsche Forschungsgemeinschaft (DFG KU 1037/13-1).

**References**

1. Sabrane K, Kruse MN, Fabritz L, Zetsche B, Mitko D, Skryabin BV, Zwiener M, Baba HA, Yanagisawa M, Kuhn M. 2005, ‘Vascular endothelium is critically involved in the hypotensive and hypovolemic actions of atrial natriuretic peptide’ J Clin Invest. 2005;115:1666–74.
2. Kuhn M. 2016, ‘Molecular Physiology of Membrane Guanylyl Cyclase Receptors’, Physiol Rev. 2016;96:751–804.
3. Khambata RS, Panayiotou CM, Hobbs AJ 2011, ‘Natriuretic peptide receptor-3 underpins the disparate regulation of endothelial and vascular smooth muscle cell proliferation by C-type natriuretic peptide’, Br J Pharmacol. 2011;164:584–97
4. Wu W, Shi F, Liu D, Ceddia RP, Gaffin R, Wei W, Fang H, Lewandowski ED, Collins S. 2017, ‘Enhancing natriuretic peptide signaling in adipose tissue, but not in muscle, protects against diet-induced obesity and insulin resistance’, Sci Signal. 2017;10(489):eaam6870

## P5 Identification of novel cGKIa substrates mediating axon bifurcation in embryonic DRG neurons

### Alexandra Böttcher^1^, Robert M. Blanton^2^, Boris Macek^3^, Ana Velic^3^, Robert Feil^1^, Hannes Schmidt^1^

#### ^1^University of Tübingen, Interfaculty Institute of Biochemistry, Tübingen Baden-Württemberg, Germany; ^2^Tufts Medical Center, Molecular Cardiology Research Institute, Boston Massachusetts, USA; ^3^University of Tübingen, Proteome Center Tübingen, Tübingen Baden-Württemberg, Germany

##### **Correspondence:** Alexandra Böttcher (alexandra.boettcher@uni-tuebingen.de)

*J Transl Med* 2022, **21(1)**:P5

**Introduction:** Developmental axon branching plays a critical role in the formation of neuronal circuitry. Neurons of dorsal root ganglia (DRG) are a useful tool to study these processes, as they exhibit axonal branching patterns in a highly stereotyped manner. Our group could dissect a cGMP-dependent signaling cascade controlling axon bifurcation in these neurons: binding of C-type natriuretic peptide (CNP) to the receptor guanylyl cyclase B (GC-B) leads to production of cGMP and thus activation of the cGMP-dependent kinase type Iα (cGKIα) [1]. To further elucidate this pathway, we aimed for the characterization of phosphorylation substrates of cGKI that are involved in the process of growth cone splitting.

**Methods:** Phosphoproteomic analysis was performed on tissue lysates of embryonic DRGs under three conditions. Samples of wild type (WT) mice and cGKI knock-out (KO) mice were treated with CNP, and each compared to an unstimulated WT control. Several putative cGKI phosphorylation substrates were selected for further analysis. The interaction of candidate proteins with cGKIα was characterized in GST pull down assays using the following as bait: the leucine zipper (LZ)-binding domain of cGKIα (cGKIα^1−59^), an LZ mutant (cGKIα^LZM^) with disrupted substrate binding, and a larger N-terminal piece of the kinase (cGKIα^1−236^) [2]. The functional role of the cGKIα LZ domain in sensory axon bifurcation was analyzed by DiI-labeling of DRG neurons in spinal cord whole mount preparations of a cGKIα-LZM mouse model [3].

**Results:** Mass spectrometric analysis of phospho-peptides derived from embryonic DRGs revealed 23 unique sites with significantly increased phosphorylation at the cGKIα consensus sequence after stimulation by CNP. Preliminary results from pull down assays using FLAG-tagged fusions of candidates indicate interaction to a higher degree with cGKIα^1−59^ and cGKIα^1−236^ than with cGKIα^LZM^. Labeling of embryonic DRG neurons of cGKIα-LZM mice with the lipophilic tracer DiI revealed a partially impaired bifurcation of sensory axons compared to WT mice.

**Conclusions:** Discovering further elements of the cGMP-dependent signaling cascade controlling bifurcation in sensory neurons during embryonic development should facilitate a better mechanistic understanding of axonal branching. Our preliminary results suggest that the LZ-binding domain of cGKIα plays a role in facilitating the phosphorylation of substrates in this context.

**Funding:** This work was funded by the Deutsche Forschungsgemeinschaft (DFG) with project 335549539/GRK2381. Additional financial support was granted by the Reinhard Frank-Stiftung.

**References**

1. Schmidt, H et al. 2009, ‘C-type natriuretic peptide (CNP) is a bifurcation factor for sensory neurons’, *Proceedings of the National Academy of Sciences*, 106, 16847–16852
2. Calamaras, TD et al. 2021, ‘MLK3 mediates impact of PKG1α on cardiac function and controls blood pressure through separate mechanisms’, *JCI Insight*, 6(18), e149075
3. Schmidt, H, Rathjen, FG 2011, ‘DiI-Labeling of DRG Neurons to Study Axonal Branching in a Whole Mount Preparation of Mouse Embryonic Spinal Cord’, *Journal of Visualized Experiments*, e3667

## P6 Specific function of membrane-bound cGMP generator GC-A for LTP-dependent central auditory adaptation processes

### Dila Calis^1^, Morgan Hess^1^, Philine Marchetta^1^, Wibke Singer^1^, Robert Lukowski^2^, Peter Ruth^2^, Marlies Knipper^1^, Lukas Rüttiger^1^

#### ^1^Eberhards-Karl-Universität Tübingen, Department of Otolaryngology, Head and Neck Surgery, Tübingen Baden-Württemberg, Germany; ^2^Eberhards-Karl-Universität Tübingen, Department of Pharmacology, Toxicology and Clinical Pharmacy, Institute of Pharmacy, Tübingen Baden-Württemberg, Germany

##### **Correspondence:** Dila Calis (dilacalis@gmail.com)

*J Transl Med* 2022, **21(1)**:P6

**Introduction:** We previously observed that the deletion of the mineralo- and glucocorticoid receptors (MR and GR) using a CaMKIIα-based tamoxifen-inducible *Cre*^ERT2^/loxP approach resulted in mice with normal hearing thresholds but differentially disturbed peripheral auditory processing upon central deletion of either MR and/or GR (Marchetta et al., iScience, 2022). The data also pointed to significant differences in adaptive responses: MR-deficient mice exhibited reduced auditory nerve responses that, however, could be centrally compensated through neural gain (increased ABR wave IV/I ratio). On the other hand, the deletion of central GR resulted in deteriorated synchronization of both peripheral and central auditory processing that generally reduced neural auditory output. Moreover, we observed that central neural auditory responses to age-related cochlear synaptopathy are dependent on proper hippocampal long-term potentiation (LTP) and short-term paired-pulse facilitation (PPF) responses, which are modulated by stress and cGMP signaling (Savitska et al., Front Mol Neurosci, under revision). Herein, we ask to what extent the MR- or GR-dependent feedback loops on cochlear processes are associated with altered hippocampal LTP and PPF levels and cGMP generator expression profiles.

**Methods:** Hippocampal LTP and PPF in double MRGRCaMKIIαCreERT2 and single MRCaMKIIαCreERT2 or GRCaMKIIαCreERT2 mice were studied. In addition, the expression profile of nitric oxide-sensitive (NO-GC) and membrane-bound (GC-A) guanylyl cyclase isoforms were studied in parallel to excitatory (Arc) and inhibitory marker proteins (PV) in hippocampus.

**Results:** The data reveal a surprising discrepancy between the NO-GC and GC-A expression profile in the hippocampus relative to their contribution to hippocampal LTP and PPF.

**Conclusions:** This may indicate a previously underestimated function of membrane-bound cGMP generators such as GC-A for central adaptation processes.

**Funding:** This work was funded by the Deutsche Forschungsgemeinschaft FOR 2060 project RU 713/3-2 and the GRK 2381 Projektnummer 335549539.

**Reference**

1. Marchetta, P., Eckert, P., Lukowski, R., Ruth, P., Singer, W., Rüttiger, L., & Knipper, M. 2022, ‘Loss of central mineralocorticoid or glucocorticoid receptors impacts auditory nerve processing in the cochlea’, *iScience*, Vol. 25,3

## P7 Heme-independent sGC activators: mechanism of action identifies the heme-free sGCβ-hsp90 species as the primary target for activating cGMP production in living cells

### Yue Dai, Dennis Stuehr

#### Cleveland Clinic, Inflammation and Immunity, Cleveland Ohio, USA

##### **Correspondence:** Yue Dai (daiy@ccf.org)

*J Transl Med* 2022, **21(1)**:P7

**Introduction:** BAY58-2667 (BAY58) is a typical example of a NO and heme-independent soluble guanylyl cyclase (sGC) activator that targets the heme-oxidized and heme-free forms of sGC. We sought to understand precisely how BAY58 activates either sGC species, in order to understand the mechanisms and to determine which species is the primary target for BAY58 activation in living cells.

**Methods:** We performed cell culture experiments on HEK293 cell co-transfected with wild-type sGCα and sGCβ, an hsp90-binding defective sGCβ del265-271, or a sGCα-binding defective sGCβ L269D/I272S/V275D and studied BAY58 effects on the extent and relative kinetics of (1) hsp90 dissociation from heme-free (apo) sGCβ using fluorescence polarization, (2) heme loss from ferric sGC using a FlAsH fluorescence tag, (3) sGCβ and sGCα heterodimerization using FRET, and (4) cGMP production using a FRET PKG cGMP biosensor. These approaches allowed us to continuously follow changes in real time and thus obtain the kinetics of each process.

**Results:** We found: (i) BAY58 immediately initiated hsp90 dissociation from apo-sGCβ and then initiated sGCαβ heterodimerization in cells at a slightly slower rate than hsp90 dissociation. (ii) The kinetics of BAY58-triggered cGMP accumulation in this circumstance showed an initial 4 min delay and then accumulated according to kinetics similar to sGC heterodimer formation. (iii) When a pre-formed heme-free sGC heterodimer was present in the cells (formed with sGCα and the sGCβ del265-271 variant), BAY58 initiated an immediate and faster cGMP accumulation. (iv) In contrast, BAY58 initiation of heme loss from ferric-sGC was very delayed (20 min) in cells and was then followed by a relatively slow cGMP accumulation.

**Conclusions:** The kinetics of BAY58 activation of the two different sGC species for cGMP synthesis indicate it overwhelmingly favors activation of the apo-sGCβ-hsp90 species over the ferric heme sGC species in living cells. The mechanism of BAY58 activation of apo-sGCβ-hsp90 first involves it’s causing a near-coincident loss of hsp90 and the binding of sGCα, followed by a relatively faster activation of the newly-formed heterodimer for cGMP synthesis. The BAY58-triggered protein partner exchange in sGCβ (Hsp90 dissociation/sGCα binding) is the slow step in the activation process. Our findings define the mechanism of action for this important class of sGC agonists and better explain how they function in living cells to activate cGMP production.

**Acknowledgements:** We thank NIH for support and thank Dr. A. Papapetropoulos for the pCMV5-sGCα and pCMV5-sGCβ constructs and Dr. F.O. Levy for the cGMP sensor.

## P8 C-type natriuretic peptide offsets the development of pulmonary hypertension

### Joshua P. Dignam, Cristina Perez-Ternero, Aisah Aubdool, Adrian J. Hobbs

#### Queen Mary University of London, William Harvey Research Institute, Barts & London School of Medicine, London, UK

##### **Correspondence:** Joshua P. Dignam (j.p.dignam@qmul.ac.uk)

*J Transl Med* 2022, **21(1)**:P8

**Introduction:** Pulmonary hypertension (PH) is a multi-faceted disease characterised by vascular remodelling and elevated pressure in the pulmonary arteries. Whilst targeted pharmacological and surgical treatments significantly improve outcomes in some PH subgroups, many patients still develop fatal right heart failure, even with optimal therapy. In the systemic circulation, C-type natriuretic peptide (CNP) modulates vascular tone, angiogenesis, inflammation, smooth muscle and endothelial cell proliferation, and cardiomyocyte hypertrophy and fibrosis, yet a (patho)physiological function for CNP in the cardiopulmonary circulation has not been established. Exploiting cell-specific transgenic mouse strains, we explored the role of CNP in the development of experimental PH and right ventricular hypertrophy (RVH).

**Methods:** Wildtype (WT), endothelial CNP knockout (ecCNP^−/−^), cardiomocyte CNP knockout (cmCNP^−/−^) and global natriuretic peptide receptor (NPR)-C nullanimals (NPR-C^−/−^) were exposed to normoxia (21% O_2_; 5 weeks; ‘controls’) or hypoxia (10% O_2_; 5 weeks) plus the vascular endothelial growth factor (VEGF) receptor antagonist Sugen (3 × 20 mg/kg; subcutaneous; days 0, 7 & 14). Pulmonary hypertension was quantified by right heart catherisation and determination of right ventricular systolic pressure (RVSP), and heart chamber weights were measured to evaluate RVH (right ventricle to left ventricle plus septum ratio; RV/[LV + S]). In some studies, echocardiography was employed sequentially to provide a structural and functional picture of the pulmonary circulation and right ventricle. Administration of CNP via subcutaneous mini-pumps was used to investigate the therapeutic potential of CNP in PH, and to examine the effects of receptor-specific CNP signaling.

**Results:** Endothelial CNP knockout mice exposed to hypoxia plus Sugen exhibited increased RVSP and RV/[LV + S] versus WT controls (Figure 1). NPR-C^−/−^ animals did not develop increased RVSP (Figure 1), and administration of CNP via subcutaneous mini-pump to both WT and NPR-C^−/−^ mice with established PH significantly reduced RVSP, suggesting that the effects of CNP on pulmonary vascular tone are primarily mediated by NPR-B. Despite the absence of a pressure phenotype, the development of RVH was exacerbated in NPR-C^−/−^ animals. However, neither RVSP nor RVH was aggravated in cmCNP^−/−^ mice, suggesting the cardiomyocyte is not an important source of CNP in the setting of PH.

**Conclusions:** In sum, these data elucidate the role of CNP in the regulation of pulmonary vascular tone and identify NPR-C as a modulator of RVH. Targeting this signalling pathway may be of therapeutic benefit in PH.
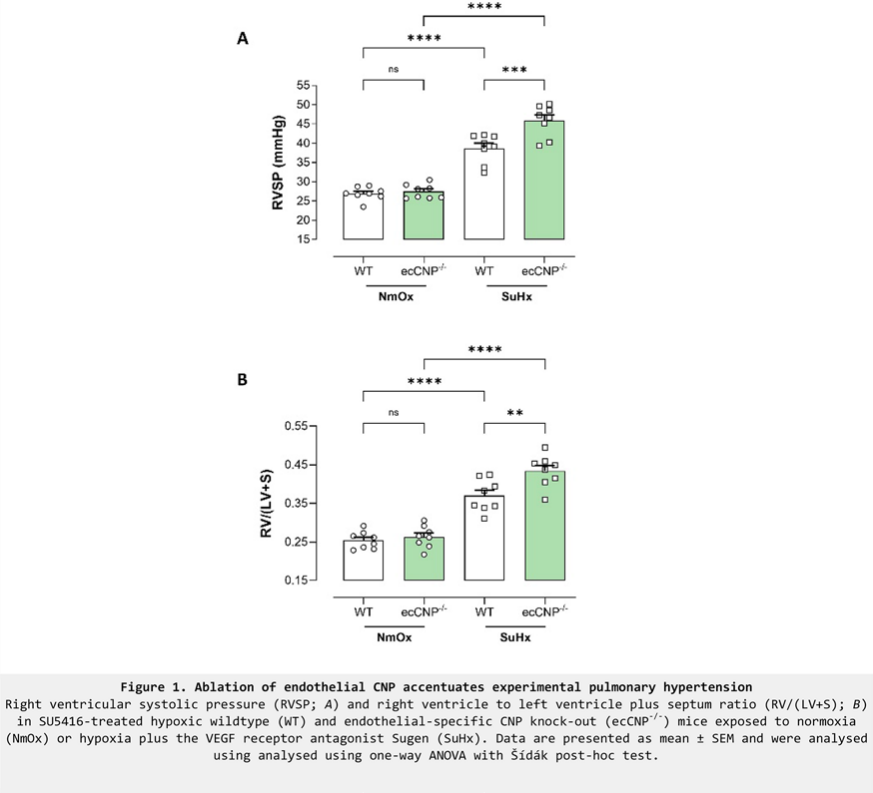


## P9 Isoform-specific interaction profiles of *human* PKG and specific cGMP analogues point to novel strategies for treatment of retinal degeneration

### Alexandra Fachinger^1^, Zhan Zhou^2^, Misheel Solongo^1^, Philipp Henning^1^, Arianna Tolone^2^, Francois Paquet-Durand^2^, Friedrich W. Herberg^1^

#### ^1^Universität Kassel, Department of Biochemistry, Kassel, Germany; ^2^Eberhards-Karl-Universität Tübingen, Cell Death Mechanisms Group, Institute for Ophthalmic Research, Tübingen, Germany

##### **Correspondence:** Alexandra Fachinger (alexfachinger@uni-kassel.de)

*J Transl Med* 2022, **21(1)**:P9

**Introduction:** cGMP is a primary messenger in the visual signalling cascade. Different lines of evidence point at cGMP-dependent protein kinase (PKG) being involved in visual signalling as well. The presence of the kinase in the human retina has been established, though isoform specific detection is not possible with available antibodies. Several forms of hereditary retinal degeneration (RD), a cluster of diseases that lead to photoreceptor degeneration and consequently, blindness of patients, display increased cGMP levels in photoreceptor cells. This renders PKG misregulation a plausible cause for rod photoreceptor cell death, which leads to a secondary loss of cone photoreceptors. In different RD mouse models, the cGMP analogue Rp-8-Br-PET-cGMPS, a known PKG antagonist, as well as the novel analogue Rp-8-Br-pMe-PET-cGMPS were found to rescue photoreceptor morphology and function.

**Methods:** For the investigation of target-specificity of Rp-8-Br-PET-cGMPS and Rp-8-Br-pMe-PET-cGMPS the three human PKG isoforms Iα, Iβ, and II were recombinantly expressed and purified. cGMP binding and kinase activity of the respective isoforms were quantified. RNAscope experiments were performed to determine which PKG isoform might be present in the murine retina and therefore which effect of the analogues might be desirable for potential drug development.

**Results:** In kinase assays both analogues displayed, to varying degrees, antagonistic effects on the activity of the three different PKG isoforms. This effect was seen most prominently for PKG Iβ, where an IC_50_-value of 250 nM was measured for Rp-8-Br-pMe-PET-cGMPS. Interestingly, Rp-8-Br-PET-cGMPS and even more so Rp-8-Br-pMe-PET-cGMPS had a partial agonistic effect on PKG Iα that was not observed for PKG Iβ and II. Initial RNAscope experiments indicate the presence of PKG II mRNA throughout the retina. To screen for potential off-target effects, both analogues were tested for direct inhibition of a PKG II construct encompassing the catalytic subunit without the cyclic nucleotide binding domain. Furthermore, the cAMP-dependent protein kinase (PKA) was tested. The established cGMP analogue Rp-8-pCPT-PET-cGMPS was able to inhibit both kinases, most likely by an ATP competitive mechanism. To a limited extent this effect was observed for Rp-8-Br-PET-cGMPS and Rp-8-Br-pMe-PET-cGMPS.

**Conclusions:** Kinase assay data strongly suggests that Rp-8-Br-PET-cGMPS and Rp-8-Br-pMe-PET-cGMPS display isoform specific effects and act via the cyclic nucleotide binding domains. Moreover, the presence of PKG II mRNA throughout the murine retina may highlight the importance of PKG II targeting analogues for further research.

**Funding:** Grant BMBF TargetRD 16GW0270 to F.W.H and F.P-D. is acknowledged.

## P10 C-type natriuretic peptide—Guanylyl cyclase B signalling counter-regulates the activation of human lung fibroblasts to myofibroblasts

### Anna-Lena Friedrich^1^, Rene Weyer^1^, Eva Lessmann^1^, Lisa Krebes^1^, Hannes Schmidt^2^, Clemens Ruppert^3^, Andreas Günther^3^, Michaela Kuhn^1^, Swati Dabral^1^

#### ^1^University of Würzburg, Institute of Physiology, Würzburg Bavaria, Germany; ^2^University of Tübingen, Interfaculty Institute of Biochemistry, Tübingen Baden-Württemberg, Germany; ^3^University of Gießen and Marburg, Lung Center (UGMLC), Gießen Hesse, Germany

##### **Correspondence:** Anna-Lena Friedrich (friedrich.anna-lena@gmx.de)

*J Transl Med* 2022, **21(1)**:P10

**Introduction:** Idiopathic Pulmonary Fibrosis (IPF) is a progressive and fatal parenchymal lung disease characterized by pathologically altered lung fibroblasts (myofibroblasts) exhibiting increased proliferation, migration and collagen production. Fibrogenic factors such as Platelet derived growth factor-BB (PDGF-BB) contribute to the initiation and progression of these pathological changes. Endogenous counter-regulating factors are barely known. Preclinical studies revealed that C-type Natriuretic Peptide (CNP), via guanylyl cyclase-B (GC-B)/cGMP signalling, attenuates pathological tissue remodelling, especially heart and lung fibrosis (1–3). To address the clinical relevance, here we compared GC-B expression, signalling and functions in cultured human lung fibroblasts obtained from “healthy” controls and patients with IPF.

**Methods:** See below.

**Results:** Human lung fibroblasts were obtained from the UGMLC biobank. Notably, western blot analyses of enriched cell membranes demonstrated that GC-B expression levels were significantly increased in IPF fibroblasts. CNP (10 and 100 nM) markedly enhanced fibroblast cGMP levels. Intriguingly, despite increased GC-B expression, the cGMP responses of IPF fibroblasts were slightly diminished in comparison to controls, suggesting that GC-B was partly desensitized. To follow-up this possibility, we performed guanylyl cyclase activity assays with crude membranes. In accordance with enhanced GC-B expression, IPF fibroblasts showed higher maximal, detergent (Triton)-stimulated guanylyl cyclase activity. We are now investigating CNP-induced guanylyl cyclase activities in control and IPF fibroblasts as well as the impact of PDGF-BB. Importantly, CNP pretreatment markedly and similarly reduced PDGF-BB-induced proliferation and migration of control and IPF lung fibroblasts as analyzed by BrdU incorporation and scratch assays, respectively. Furthermore, in both groups CNP strongly decreased PDGF-BB-induced collagen 1 and 3 expression as measured by immunocytochemistry and immunoblotting.

**Conclusions:** CNP moderates the PDGF-BB-induced activation and differentiation of human lung fibroblasts to myofibroblasts and this effect is preserved in IPF fibroblasts. Understanding the signalling pathways of CNP in fibroblasts may unravel novel targets for therapies of IPF.

**Funding:** This study was funded by the Deutsche Forschungsgemeinschaft (DFG KU 1037/6-1, to MK) and the Else-Kröner-Fresenius Stiftung (2020 EKEA.131, to SD).

**References**

1. Kuhn M. Molecular Physiology of Membrane Guanylyl Cyclase Receptors. Physiol Rev. 2016;96:751–804.
2. Moyes AJ, Chu SM, Aubdool AA, Dukinfield MS, Margulies KB, Bedi KC, Hodivala-Dilke K, Baliga RS, Hobbs AJ. C-type natriuretic peptide co-ordinates cardiac structure and function. Eur Heart J. 2020;41:1006–1020.
3. Kimura T, Nojiri T, Hino J, Hosoda H, Miura K, Shintani Y, Inoue M, Zenitani M, Takabatake H, Miyazato M, Okumura M, Kangawa K. C-type natriuretic peptide ameliorates pulmonary fibrosis by acting on lung fibroblasts in mice. Respir Res. 2016;17:19.

## P11 sGC stimulator (BAY 41-8543) for the treatment of heart failure with reduced ejection fraction (HFrEF) and cardio-renal syndrome

### Olga Gawrys^1^, Zuzana Husková^1^, Petra Škaroupková^1^, Zuzana Honetschlägerová^1^, Zdenka Vaňourková^1^, Sona Kikerlová^1^, Vojtěch Melenovský^2^, Luděk Červenka^1^

#### ^1^Institute for Clinical and Experimental Medicine, Center for Experimental Medicine, Prague, Czech Republic; ^2^Institute for Clinical and Experimental Medicine, Department of Cardiology, Prague, Czech Republic

##### **Correspondence:** Olga Gawrys (olga.gawrys@ikem.cz)

*J Transl Med* 2022, **21(1)**:P11

**Introduction:** Heart failure with reduced ejection fraction (HFrEF) is considered to be one of the major epidemics of the twenty-first century. The prognosis and life expectancy is especially dreadful for patients that develop concurrent impairment of kidney function, so called “cardio-renal syndrome”. More recent treatment strategies for HFrEF target NO-sGC-cGMP pathway by sGC stimulators. These compounds bind allosterically to the heme-moiety of the sGC and stimulate the sGC in the absence of NO (NO-independent action), but also they sensitize sGC to low levels of endogenous NO by stabilizing NO–sGC binding.

**Methods:** In the present study we investigated the effectiveness of the sGC stimulator BAY 41-8543, *in* ren-2 transgenic hypertensive rats (TGR) with aorto-caval fistula (ACF)-induced HFrEF (due to volume overload) and renal dysfunction. The long-term effectiveness of the sGC stimulator administered alone (3 mg/kg/day in food) or combined with an ACE inhibitor (Trandolapril, 0.25 mg/kg/day in drinking water) on the survival rate of ACF-induced HFrEF in TGR was investigated in a 30 week treatment study. In separate series of experiments (short treatment regimen, 2 weeks of treatment) the impact of BAY 41-8543 on blood pressure was measured by telemetry technique (Data Sciences International, DSI, USA). In addition, urine, blood and tissue samples were collected to measure cGMP levels, albuminuria and nitric oxide metabolites exertion during two-week treatment.

**Results:** BAY 41-8543 significantly improved the survival rate of ACF TGR in comparison to untreated animals with HFrEF. However, BAY 41-8543 administered together with ACEi decreased the beneficial activity of the ACEi. The overall survival in BAY 41-8543 + ACEi treated group was 50% by the end of 30 weeks, while in the group treated only with ACEi it was almost 90% (Figure 1). In addition, a minor and transient decrease in blood pressure (− 10 mmHg) was observed two days after BAY 41-8543 administration, but early on SBP started to rise again and by the end of the two week observation it was on the same level as in untreated ACF TGR (127 ± 3 vs 131 ± 6 mmHg, respectively; NS).

**Conclusions:** In summary, it seems that in this pilot-study, the NO-independent stimulation of sGC is a promising strategy to treat HFrEF in humans resulting in an improved survival. However, the results in the combination group, need confirmation and additional analysis, since dose-selection of BAY 41-8543 might not be optimal for the combination treatment (e.g. due to hypotension).
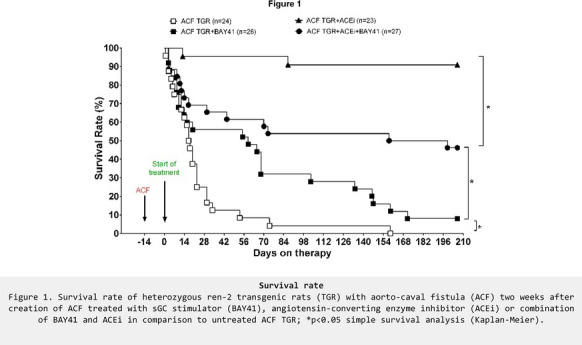


## P12 Distinct functions of natriuretic peptide receptor 2 in pain processing

### Hannah Gerninghaus^1^, Lea Kennel^1^, Anja Kaiser^1^, Fangyuan Zhou^1^, Cathrin Flauaus^1^, Jonas Petersen^2^, Tilman Groß^1^, Wiebke Kallenborn-Gerhardt^1^, Hannes Schmidt^3^, Achim Schmidtko^1^

#### ^1^Goethe University, Institute of Pharmacology and Clinical Pharmacy, Frankfurt Hesse, Germany; ^2^University of Münster, Institute of Immunology, Münster, Germany; ^3^University of Tübingen, Interfaculty Institute of Biochemistry, Tübingen, Germany

##### **Correspondence:** Hannah Gerninghaus (h.gerninghaus@em.uni-frankfurt.de)

*J Transl Med* 2022, **21(1)**:P12

**Introduction:** Increasing evidence indicates that cGMP signaling in the peripheral nervous system and the spinal cord contributes to the processing of pain. In addition to NO-dependent cGMP production, recent data suggest an involvement of particulate guanylate cyclases in nociceptive signaling. Here, we assessed the functional role of particulate guanylate cyclase B (Npr2; natriuretic peptide receptor 2) in pain processing using tissue-specific Npr2 knockout mice.

**Methods:** The expression pattern of Npr2 in dorsal root ganglia (DRG) and the spinal cord of mice was investigated by fluorescent in situ hybridization, immunohistochemistry and qRT-PCR. The behavior of mice lacking Npr2 conditionally in DRG neurons was assessed in various animal models of pain.

**Results:** We found Npr2 to be expressed in the majority of DRG neurons and co-expressed with cGMP-dependent protein kinase I. Interestingly, Npr2-deficient mice displayed altered responses in models of acute nociceptive pain and persistent inflammatory pain.

**Conclusions:** We here provide evidence for a so far unrecognized function of Npr2/cGMP signaling in DRG neurons during the processing of pain.

**Funding:** This work is supported by grants from the Deutsche Forschungsgemeinschaft (DFG; German Research Foundation). The authors have no conflicts of interest to declare.

## P13 Development of a vascularized complex human 3D in vitro skin by combination of iPSC-derived skin organoids and vascular organoids to mimic a vascular network

### Amelie Reigl^1^, Florian K. Groeber-Becker^1,3^, Philipp Wörsdörfer^4^, Andreas Friebe^2^, Dieter Groneberg^2,3^

#### ^1^University of Würzburg, Institute of Tissue Engineering and Regenerative Medicine, Würzburg Bavaria, Germany; ^2^University of Würzburg, Institute of Physiology, Würzburg Bavaria, Germany; ^3^Fraunhofer-Institute for Silicate Research, Translational Center for Regenerative Therapies, Würzburg Bavaria, Germany; ^4^University of Würzburg, Institute of Anatomy and Cell Biology, Würzburg Bavaria, Germany

##### **Correspondence:** Dieter Groneberg (dieter.groneberg@uni-wuerzburg.de)

*J Transl Med* 2022, **21(1)**:P13

**Introduction:** Human skin organoids derived from induced pluripotent stem cells (iPSC) provide unique opportunities for the study of mechanisms of morphogenesis and diseases to complement animal studies. Our current in vitro skin model consists of the two main cell types of skin, i.e., epidermal keratinocytes and dermal fibroblasts, extracted from the foreskin of human donors. Both cell types are combined with a collagen scaffold to mimic dermis and epidermis and cultivated in an air–liquid interface. These three-dimensional (3D) full-thickness skin equivalents (FTSE) are suitable as simple pharmacological test systems.

**Methods:** To improve our in vitro skin model, we use self-organized skin organoids, which create complex tissue via cell–cell interaction. These organoids mimic complex structures like hair follicles, neuronal networks and other cell types (e.g., adipocytes, chondrocytes) which can improve our current skin model. However, iPSC-derived skin organoids lack a functional vasculature. To address this drawback, we used a second iPSC-derived vascular organoid to introduce a vascular network into the in vitro skin model.

**Results:** We could show that endothelial cells (CD31^+^) and smooth muscle cells (SMMHC^+^) form tube-like structures growing into the FTSE. Cells of the vascular organoid express NO-GC, the main target for NO which plays an important role during angiogenesis and arteriogenesis. Vascular organoids represent a perfect tool to study morphogenesis of human vasculature and serve as in vitro test systems for disease modelling and drug screening. We already embedded vascular organoids into FTSE to generate vascularized FTSE. In parallel, skin organoids are generated to be embedded into the vascularised FTSE to form a vascularized complex human 3D in vitro skin.

**Conclusions:** In summary, human in vitro 3D vascularized skin will shed light on the role of NO/cGMP signalling in angiogenesis and will help to develop cGMP-modulating drugs to treat skin defects and improve skin regeneration.

## P14 The sGC stimulator *vericiguat* reduced hypertension-induced renal damage

### Sarah M. Kedziora^1,2,3,4^, Hanna Napieczynska^2^, Peter Sandner^5^, Arnd Heuser^2^, Ralf Dechend^1,3,4,6^, Dominik N. Müller^1,2,3,4^, Nadine Haase^1,2,3,4^, In collaboration with Bayer AG Pharmaceuticals

#### ^1^Experimental and Clinical Research Center, a cooperation of Max Delbrück Center for Molecular Medicine and Charité - Universitätsmedizin Berlin, Berlin, Germany; ^2^Max-Delbrück-Center for Molecular Medicine in the Helmholtz Association (MDC), Berlin, Germany; ^3^Charité –Universitätsmedizin Berlin, corporate member of Freie Universität Berlin and Humboldt-Universität zu Berlin, Experimental and Clinical Research Center, Berlin, Germany; ^4^German Center for Cardiovascular Research (DZHK), partner site Berlin, Berlin, Germany; ^5^Bayer AG, Pharmaceuticals, Global Drug Discovery, Wuppertal, Germany; ^6^HELIOS Clinic, Department of Cardiology and Nephrology, Berlin, Germany

##### **Correspondence:** Nadine Haase (nadine.haase@mdc-berlin.de)

*J Transl Med* 2022, **21(1)**:P14

**Introduction:** Chronic kidney disease (CKD) is the result of hypertension-induced end organ damage. Despite blood-pressure control, CKD is progressing, since both pressure-dependent and -independent mechanisms can contribute to renal damage. Soluble guanylyl cyclase (sGC) stimulation addresses an important signalling pathway in the cardio, renal and vascular system. We hypothesize that sGC stimulation ameliorates CKD in an established rat model of hypertension.

**Methods:** We used double transgenic rats harboring both human renin and angiotensinogen genes (dTGRs). These rats develop moderately severe hypertension but die of end-organ cardiac and renal damage by week 7. We aimed to investigate the effect of the sGC stimulator *vericiguat* on renal damage in these hypertensive rats. To investigate blood pressure-dependent and -independent effects, we used different doses of *vericiguat*. We treated 4-week-old male double transgenic rats (dTGRs) with either 0.3 mg/kg/d, 1.0 mg/kg/d, 3.0 mg/kg/d *vericiguat* or vehicle once daily for 3 weeks by oral gavage. Age-matched vehicle-treated Sprague Dawley rats served as healthy wild-type (WT) controls.

**Results:** Mean arterial pressure was dose dependently reduced after treatment with 1.0 or 3.0 mg/kg/d *vericiguat* compared to vehicle. The lowest dose of 0.3 mg/kg/d *vericiguat* had no effect on the blood pressure, whereas 1.0 or 3.0 mg/kg lead to moderate decrease of mean arterial pressure of 20 and 40 mmHg, respectively. Treatment with 1.0 or 3.0 mg/kg/d *vericiguat* resulted in less albuminuria throughout the experiment whereas the low dose reduced albuminuria only in weeks 5 to 6. Analysis of the renal microvasculature by μCT showed a reduced microvascular density in the vehicle treated dTGRs compared to wild-type. However, the treatment with *vericiguat* had no impact on these changes. Histological characterization of renal damage showed an improvement of the tubular and glomerular damage by the treatment as well as an improvement of renal fibrosis and inflammation. Levels of renal biomarkers for kidney damage were also ameliorated under *vericiguat* treatment.

**Conclusions:** The sGC stimulator *vericiguat* dose-dependently ameliorated hypertensive induced renal damage.

## P15 Fast auditory processing deficits in *Bdnf*^*Pax2*^ KO mice interact with the neuronal disbalance in soluble and particulate cGMP generator levels

### Morgan Hess^1^, Philine Marchetta^1^, Robert Lukowski^2^, Peter Ruth^2^, Kerstin Schwabe^3^, Wibke Singer^1^, Lukas Rüttiger^1^, Marlies Knipper^1^

#### ^1^University of Tübingen, University ENT Clinic Tübingen, Molecular Physiology of Hearing, Tübingen Hearing Research Center, Tübingen Baden-Württemberg, Germany; ^2^University of Tübingen, Department of Pharmacology, Toxicology and Clinical Pharmacy, Institute of Pharmacy, Tübingen Baden-Württemberg, Germany; ^3^Hannover Medical School, Department of Neurosurgery, Hannover Lower Saxony, Germany

##### **Correspondence:** Morgan Hess (morgan.hess@uni-tuebingen.de)

*J Transl Med* 2022, **21(1)**:P15

**Introduction:** Brain-derived neurotrophic factor (*Bdnf*)deletion by Pax2-driven Cre recombinase was previously shown to lead to the absence of BDNF in Pax2-derived GABAergic interneurons in the auditory periphery, while central BDNF expression levels remained unaltered [1–3]. These conditional *Bdnf*^*Pax2*^ knock-out (KO) mice have normal hearing thresholds but impaired (immature) fast auditory processing, which typically develops during a critical period of experience-dependent maturation occurring around hearing onset. The distinctly reduced auditory acuity in *Bdnf*^*Pax2*^KOs was correlated with a failed sharpening of the inhibitory strength of dorsal cochlear nucleus and inferior colliculus neurons. As a result, these regions maintained a delayed latency and narrow dynamic range, which would typically improve upon hearing onset. Such discrete alterations in auditory circuits were also correlated with impaired learning and social interaction and with deficits in the capacity to respond to auditory deprivation through compensatory increases in central neural activity [3]. Our recent findings suggested that compensatory increases in central neural activity rely on proper cGMP signaling (unpublished work).

**Methods:** Here we assessed guanylyl cyclase A (GC-A) and nitric-oxide-guanyl-cyclase (NO-GC) mRNA levels in the auditory cortex and hippocampus in *Bdnf*^*Pax2*^ KO mice and tested the effect of sound enrichment on both cGMP generator levels and on auditory and limbic function.

**Results:** Sound enrichment differentially affected auditory and limbic functions in *Bdnf*^*Pax2*^ KO mice, correlating with altered transcript levels of the cGMP generators GC-A and NO-GC in the auditory cortex and hippocampus.

**Conclusions:** Although our findings are preliminary, we conclude that the maturation and balance of cGMP generator levels may be a key step for proper development of memory-related neural adaptive responses during the critical period of development after hearing onset.

**Funding:** This work was funded by the Deutsche Forschungsgemeinschaft FOR 2060 project RU 713/3-2 and the GRK 2381; Projektnummer 335549539.

**References**

1. Zuccotti, A., Kuhn, S., Johnson, S. L., Franz, C., Singer, W., Hecker, D., Geisler, H. S., Köpschall, I., Rohbock, K., Gutsche, K., Dlugaiczyk, J., Schick, B., Marcotti, W., Rüttiger, L., Schimmang, T., & Knipper, M. 2012, ‘Lack of brain-derived neurotrophic factor hampers inner hair cell synapse physiology, but protects against noise-induced hearing loss’, *The Journal of neuroscience: the official journal of the Society for Neuroscience*, Vol. 32,25
2. Chumak, T., Rüttiger, L., Lee, S. C., Campanelli, D., Zuccotti, A., Singer, W., Popelář, J., Gutsche, K., Geisler, H. S., Schraven, S. P., Jaumann, M., Panford-Walsh, R., Hu, J., Schimmang, T., Zimmermann, U., Syka, J., & Knipper, M. 2016, ‘BDNF in Lower Brain Parts Modifies Auditory Fiber Activity to Gain Fidelity but Increases the Risk for Generation of Central Noise After Injury’, *Molecular neurobiology*, Vol. 53,8
3. Eckert, P., Marchetta, P., Manthey, M. K., Walter, M. H., Jovanovic, S., Savitska, D., Singer, W., Jacob, M. H., Rüttiger, L., Schimmang, T., Milenkovic, I., Pilz, P., & Knipper, M. 2021 ‘Deletion of BDNF in Pax2 Lineage-Derived Interneuron Precursors in the Hindbrain Hampers the Proportion of Excitation/Inhibition, Learning, and Behavior’, *Front Mol Neurosci,* Vol. 14, 642679

## P16 CY6463, a CNS-penetrant sGC stimulator, elicits benefits in preclinical models of mitochondrial complex 1 deficiency

### Emmanuel S. Buys, Susana S. Correia, Guang Liu, Joon Jung, John R. Hadcock, Peter Germano, Christopher J. Winrow, Juli E. Jones

#### Cyclerion Therapeutics, Cambridge Massachusetts, USA

##### **Correspondence:** Juli E. Jones (jjones@cyclerion.com)

*J Transl Med* 2022, **21(1)**:P16

**Introduction:** Nitric oxide (NO) is a gasotransmitter that stimulates soluble guanylate cyclase (sGC) to produce cyclic guanosine 3′,5′-monophosphate (cGMP). The NO-sGC-cGMP pathway has been reported to increase mitochondrial biogenesis and function, which are dysregulated in mitochondrial disease. We aimed to examine whether increasing cGMP via sGC stimulation alters energy reserves in cells from individuals with mitochondrial disease.

**Methods:** We studied CY6463, a clinical stage CNS-penetrant sGC stimulator, in cells from individuals with a mitochondrial complex-1 disease (Leber hereditary optic neuropathy (LHON) or Leigh syndrome). In addition, we explored the effect of CY6463 on astrogliosis in a mouse model of mitochondrial complex-1 deficiency.

**Results:** Basal ATP levels were lower in cells from LHON and Leigh syndrome patients than in those from healthy controls. ATP levels were higher in LHON cells treated with 1 µM CY6463 alone or with CY6463 (0.1 µM or 1 µM) and DETA-NO (10 µM) than in vehicle-treated LHON cells. Similarly, ATP levels were higher in Leigh syndrome cells treated with DETA-NO, CY6463 alone (0.1, 1, and 10 µM), or CY6463 at 0.1 µM with DETA-NO than in vehicle-treated Leigh syndrome cells. Expression levels of mitochondrial genes, such as TFAM, MT-CO2, MT-CO3, and MT-ND3, were lower in patient cells than in healthy cells. Expression levels of these genes were higher in patient cells treated with CY6463 than with vehicle. In an in vivo model of mitochondrial complex-1 inhibition, protein staining of glial fibrillary acidic protein (GFAP), a marker of astrogliosis that results from tissue damage and inflammation, was higher in the retina of mice challenged with a single intravitreal (IVT) injection of the mitochondrial complex-1 inhibitor rotenone than in vehicle-treated mice. Retinal astrogliosis was less pronounced in mice treated with CY6463 for 3 weeks prior to rotenone IVT injection than in vehicle-treated mice.

**Conclusions:** Together, these data suggest that, in preclinical models of mitochondrial complex-1 deficiency, CY6463 may alleviate mitochondrial dysfunction through improving cellular energetics and reducing inflammation.

## P17 Cardioprotective effect of sGC stimulator *vericiguat* in a rat model for chronic heart failure

### Sarah M. Kedziora^1,2,3,4^, Peter Sandner^5^, Kristin Kräker^1,2,3,4^, Arne Thiele^1,3,4^, Ralf Dechend^1,3,4,6^, Dominik N. Müller^1,2,3,4^, Nadine Haase^1,2,3,4^, In collaboration with Bayer AG Pharmaceuticals

#### ^1^Experimental and Clinical Research Center, cooperation of Max Delbrück Center for Molecular Medicine and Charité - Universitätsmedizin Berlin, Berlin, Germany; ^2^Max-Delbrück-Center for Molecular Medicine in the Helmholtz Association (MDC), Berlin, Germany; ^3^Charité – Universitätsmedizin Berlin, corporate member of Freie Universität Berlin and Humboldt-Universität zu Berlin, Experimental and Clinical Research Center, Berlin, Germany; ^4^German Center for Cardiovascular Research (DZHK), partner site Berlin, Berlin, Germany; ^5^Bayer AG Pharmaceuticals, Global Drug Discovery, Wuppertal, Germany; ^6^HELIOS Clinic, Department of Cardiology and Nephrology, Berlin, Germany

##### **Correspondence:** Sarah M. Kedziora (sarah.kedziora@mdc-berlin.de)

*J Transl Med* 2022, **21(1)**:P17

**Introduction:** Arterial hypertension exacerbated with left ventricular hypertrophy is a major cause for chronic heart failure. The continuously rising prevalence of HFpEF and HFrEF in humans is associated with a high rate of morbidity and mortality. Treatment strategies are still limited and improving the prognosis in heart failure remains challenging. The stimulator of the soluble guanylyl cyclase (sGC) *vericiguat* was approved for treatment of chronic heart failure (HFrEF) in 2021. In this study we aimed to characterize the mode of action of *vericiguat* on cardiovascular function and metabolism in an established rodent model of hypertension induced renal insufficiency and heart failure.

**Methods:** Four-week-old male double-transgenic rats (dTGRs) expressing human renin and angiotensinogen entered the study. We included vehicle-treated (10%/20%/70% transcutol/cremophore/water) dTGRs and wild-type control rats and dTGRs receiving 0.3, 1.0 or 3.0 mg/kg/d of *vericiguat*. We investigated the cardiac function with in vivo echocardiography including speckle tracking (STE), telemetric blood pressure measurements and profiled the cardiac metabolism in vitro. The transcriptional profile and cardiac histology were analysed.

**Results:** *Vericiguat* treatment in dTGRs significantly reduced mortality compared to vehicle-treated dTGRs (61.5%), in the 0.3, 1.0 and 3.0 mg/kg/d treatment groups 69.2%, 78.6% and 100% survived. Treatment with 3.0 mg/kg/d *vericiguat* reduced blood pressure moderately by 40 mmHg compared to vehicle-treated dTGRs. Echocardiography revealed increased ejection fraction, cardiac output, stroke volume and fractional shortening under *vericiguat* treatment, while these parameters were not changed in vehicle-treated dTGRs. STE analysis indicated diminished global and spatial longitudinal strain in vehicle-treated dTGRs and improvement by *vericiguat* in a dose-dependent manner. *Vericiguat* improved cardiac fibrosis, inflammation but cardiac hypertrophy was not attenuated. Transcriptional analysis showed improved cardiac/hypertrophy (atrial and brain natriuretic peptide, beta-myosin heavy chain) and fibrosis marker (connective-tissue growth factor) in *vericiguat*-compared to vehicle-treated dTGRs. Investigations on cardiac metabolism highlighted that *vericiguat* seems to have a positive effect on the metabolic profile (oxidative phosphorylation and glycolysis) of cardiac cells in the heart failure model.

**Conclusions:** Our data demonstrated that sGC stimulation via *vericiguat* effectively improved cardiac function and morphology, and improved survival in dTGR rat model. The results from the animal model underline the potential as a therapeutic for chronic heart failure.

## P18 Characterization of a zone-specific hepatic stellate cell subtype in the liver

### Muhammad Ashfaq Khan, Julian Fischer, Fabian Schwiering, Dieter Groneberg, Andreas Friebe

#### University of Würzburg, Institute of Physiology, Würzburg Bavaria, Germany

##### **Correspondence:** Muhammad Ashfaq Khan (muhammad.ashfaq-khan@uni-wuerzburg.de)

*J Transl Med* 2022, **21(1)**:P18

**Introduction:** Liver disease has increasingly become a health care problem affecting approx. 844 million individuals worldwide. Hepatic fibrosis is a highly dynamic process resulting in extracellular matrix accumulation and scarring of the liver; etiologies include viral infection, alcoholic liver disease, and non-alcoholic steatohepatitis (NASH). The central driver of hepatic fibrosis is the hepatic stellate cell (HSC) also known as the pericyte of the liver. HSC are the major liver cells that express NO-sensitive guanylyl cyclase (NO-GC) and NO-GC stimulators have been shown to prevent hepatic steatosis, inflammation, and fibrosis in experimental NASH in rodents. Contrary to the well-established functional zonation of hepatocytes across liver lobule (hexagonal shaped anatomical unit), the zonal heterogeneity of hepatic stellate cells across the liver lobule is less defined; so far, HSC heterogeneity has only been reported using single cell transcriptomic profile analysis. In this in vivo study, we identify and characterize a NO-GC-expressing HSC subtype specifically found in zone 1 of the healthy liver (zone 1-HSC).

**Methods:** For the lineage tracing of zone 1-HSC, tamoxifen was injected into reporter mice expressing the fluorescent dye tdTomato under the control of the smooth muscle myosin heavy chain (SMMHC) promoter (SMMHC-CreERT2). Mice were given CCl_4_ (i.p., thrice a week, 1 µg/g BW) to induce primary parenchymal fibrosis. The behavior of zone 1-HSC was examined during fibrosis progression and reversal. For immunofluorescence staining and confocal microscopy, livers were perfused with PBS, fixed in PFA/sucrose, and cryopreserved.

**Results:** In the healthy liver, SMMHC/tdTomato-positive HSC localize in zone 1 but not in other zones of the liver lobule. Liver zonation markers show that zone 1-HSC originate from progenitor cells found in proximity to the bile duct. However, upon CCl_4_ treatment, HSC zonation is lost as SMMHC/tdTomato-positive HSCs disperse and move centrally into zones 2 and 3. Furthermore, immunofluorescent confocal microscopy revealed that this HSC subtype—in contrast to regular HSC—do not transform into myofibroblasts under fibrotic conditions.

**Conclusions:** We have identified a subtype of HSC (tentatively called zone 1-HSC) in zone 1 of the liver lobule under non-fibrotic conditions. Zonation is lost after fibrosis induction, but zone 1-HSC do not transform into myofibroblasts (the main effectors of fibrosis) and do not exhibit the phenotype of activated HSC. The specific function of this HSC subtype and the role of NO-GC in this cell is under current investigation.

## P19 Soluble guanylate cyclase assembly does not occur co-translationally based on disome-selective profiling (DiSP) and selective ribosome profiling (SeRP)

### Kai Fenzl^2^, Guenter Kramer^2^, Josef J. Auburger^1^, Ilka Mathar^1^, Peter Sandner^1,3^, Jan R. Kraehling^1^

#### ^1^Bayer AG, Cardiovascular Research, Wuppertal North Rhine-Westphalia, Germany; ^2^Ruprechts-Karls-University, Zentrum für Molekulare Biologie (ZMBH), Heidelberg Baden-Württemberg, Germany; ^3^Hannover Medical School, Institute of Pharmacology, Hannover Lower Saxony, Germany

##### **Correspondence:** Jan R. Kraehling (jan.kraehling@bayer.com)

*J Transl Med* 2022, **21(1)**:P19

**Introduction:** The sGC is a heterodimeric protein complex composed of one alpha and one beta-subunit and is widely expressed in different human and animal tissues. Complex formation is obligate for enzymatic activity. The aim of this project was to investigate if the assembly of soluble guanylate cyclase (sGC) occurs co-translationally.

**Methods:** In this project the primary Rat Aortic Smooth Muscle Cell line (RAOSMC) was used, which has high endogenous expression levels of both sGC subunits (*GCYC1A1* and *GCYC1B1*).

1. Disome-selective profiling (DiSP) to explore interactions between nascent subunit for co–co assembly (Bertolini et al. 2021). DiSP relies on the separation of single ribosomes (monosomes) from nascent chain coupled ribosomes (disomes) via sucrose gradient centrifugation post RNase digestion. Sequencing of the 30 nt RNA footprints from both populations allows the detection of co–co assembly, which is revealed by a shift of ribosomes translating individual open reading frames from the monosome to the disome fraction, caused by nascent chain interactions.
2. Selective ribosome profiling (SeRP) to explore interactions between one fully synthesized subunit with its nascent interaction partner [co-post assembly (Shiber et al. 2018; Shieh et al. 2015)]. SeRP compares the mRNA distribution of translating ribosomes determined in two ribosome profiling experiments. The first profiling determines the total translatome and reveals the mRNA position of all translating ribosomes. The second profiling determines the mRNA positions of the sub-population of translating ribosomes that interact with a fully synthesized subunit of the sGC complex, purified by co-affinity purification (termed AP translatome).

**Results:** DiSP of RAOSM cells reveals established co–co assembly of Lamin A/C and Vimentin but not of the sGC alpha- and beta-subunits (Figure 1). SeRP does not support any co-post assembly mechanism of the sGC complex. Selective Ribosome Profiling indicates potential interactions of fully synthesized sGC subunits with translating ribosomes.

**Conclusions:** The hypothesis that the hetero-dimeric sGC complex assembles co-translationally, either by co–co or co-post assembly, is not supported by these results. Further studies to investigate the nature of the sGC heterodimerization will be needed.
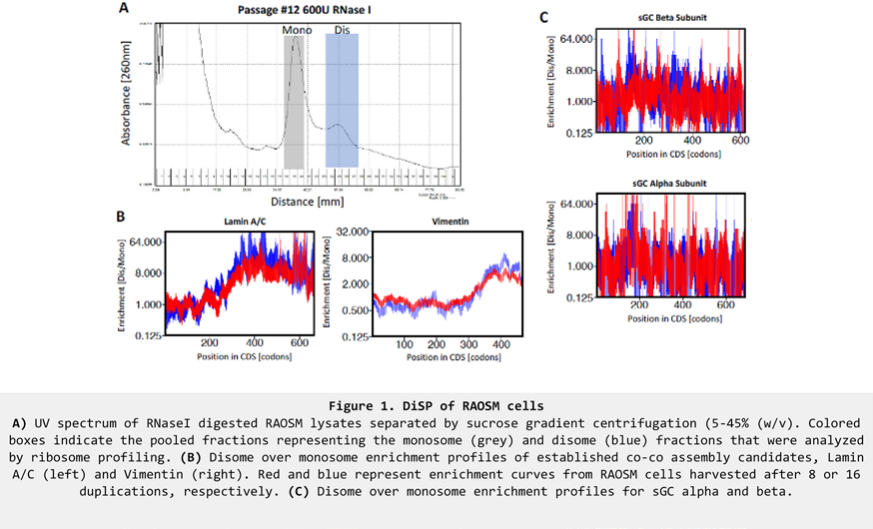


**References**

1. Bertolini, M., et al. (2021). “Interactions between nascent proteins translated by adjacent ribosomes drive homomer assembly.” Science 371(6524): 57–64.
2. Shiber, A., et al. (2018). “Cotranslational assembly of protein complexes in eukaryotes revealed by ribosome profiling.” Nature 561(7722): 268–272.
3. Shieh, Y.-W., et al. (2015). “Operon structure and cotranslational subunit association direct protein assembly in bacteria.” Science 350(6261): 678–680.

## P20 Expression and purification of 11 disease-causing sGC mutations

### Paul Kiessling^1^, Philipp Ellinger^1^, Florian Sohler^1^, Lars Linden^1^, Peter Sandner^1,2^, Jan Kraehling^1^

#### ^1^Bayer AG, Cardiovascular Research, Wuppertal North Rhine-Westphalia, Germany; ^2^Hannover Medical School, Institute of Pharmacology, Hannover Lower Saxony, Germany

##### **Correspondence:** Jan Kraehling (jan.kraehling@bayer.com)

*J Transl Med* 2022, **21(1)**:P20

**Introduction:** Mutations in sGC have been linked to diseases such as myocardial infarction (Erdmann et al. 2013; Wobst et al. 2016), pulmonary hypertension (Wilkins et al. 2014; Wallace et al. 2016), achalasia (Hervé et al. 2014; Wallace et al. 2016), and moyamoya (Hervé et al. 2014; Wallace et al. 2016). For this work 11 mutations in the alpha1-subunit identified in different human diseases (Figure 1) were selected from the literature and databases.

**Methods:** The wildtype and the selected 11 mutations have been cloned and expressed in HEK293-E6 cells. Purification of the enzymes was done by strep-affinity chromatography followed by size exclusion chromatography (SEC).

**Results:** The wildtype and all 11 mutations were cloned successfully into the pMC3025 vector. Initial expression test were done to evaluate cell viability and potential yield for these 12 enzymes. Individual enzymes expressed in HEK293-E6 cells were successfully purified by StrepTag affinity chromatography and size exclusion chromatography (SEC) in quantities varying between 20 and 500 µg per 3 l cell culture. Three enzyme mutations (G652*, T64A, and K53E) result in complete aggregation of the protein and could not be analyzed further. UV/Vis spectroscopy revealed significant heme-content (430 nm absorption/Soret band) not only for the wildtype, but also for the T229M, S478G, V587I, and the C517T mutation. Preliminary enzymatic activity data indicate remaining activity in this expression system for these four mutations: I571V, A680T, T229M, and C571T.

**Conclusions:** Overall, this works aims to systemically analyze 11 sGC mutations linked to various diseases in humans. The cloning and expression revealed three mutations causing a complete aggregation assuming a compete loss of enzymatic activity. A detailed analysis of the enzymatic activity for all soluble 8 enzyme mutations and the wildtype is currently underway and will help to understand the impact of these mutations for the NO-sGC-cGMP pathway in the affected patients and might unveil potential treatment options like NO-donors, sGC stimulators, or sGC activators.
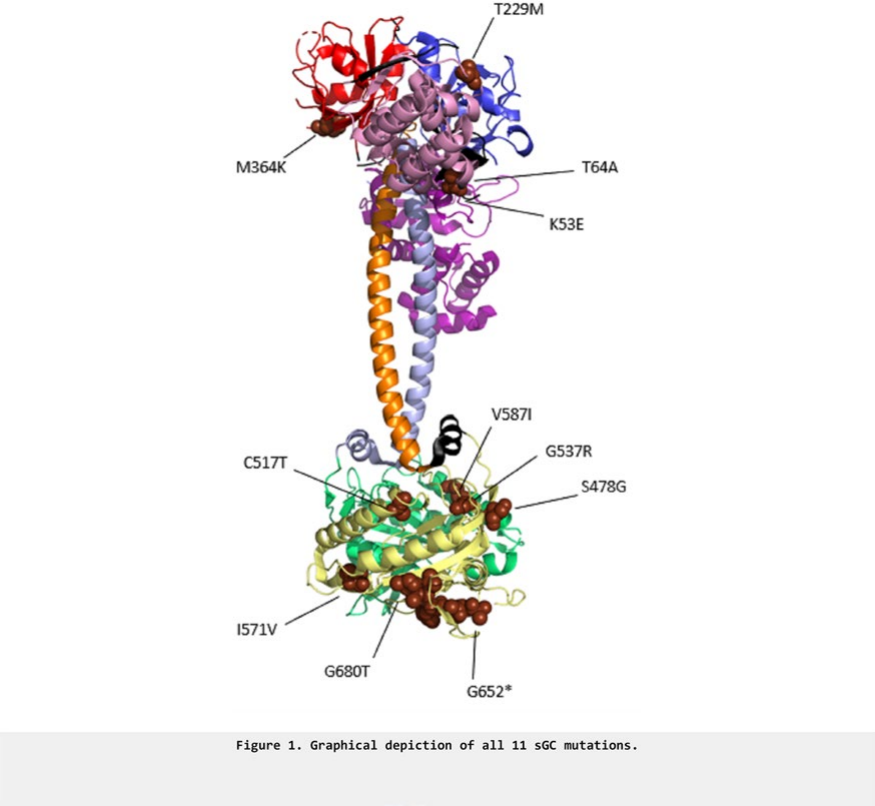


**Acknowledgements:** We would like to thank Bettina Broehland and Sophie Fahlenbock for their invaluable technical assistance.

**References**

1. Erdmann, J., et al. (2013). “Dysfunctional nitric oxide signalling increases risk of myocardial infarction.” Nature 504(7480): 432–436.
2. Wilkins, M. R., et al. (2014). “alpha1-A680T variant in GUCY1A3 as a candidate conferring protection from pulmonary hypertension among Kyrgyz highlanders.” Circ Cardiovasc Genet 7(6): 920–929.
3. Wallace, S., et al. (2016). “Disrupted nitric oxide signaling due to GUCY1A3 mutations increases risk for moyamoya disease, achalasia and hypertension.” Clin Genet 90(4): 351–360.
4. Wobst, J., et al. (2016). “Stimulators of the soluble guanylyl cyclase: promising functional insights from rare coding atherosclerosis-related GUCY1A3 variants.” Basic Res Cardiol 111(4): 51.

## P21 Runcaciguat, a novel soluble guanylate cyclase activator, shows renoprotection in hypertensive, diabetic, and metabolic preclinical models of chronic kidney disease (CKD)

### Jan R. Kraehling^1^, Tibor Schomber^1,2^, Agnes Benardeau^1,3^, Antje Kahnert^1^, Laura Popp^1^, Julia Vienenkoetter^1^, Mira Pavkovic^1^, Axel Kretschmer^1^, Heidrun Ellinger-Ziegelbauer^1^, Bettina Lawrenz^1^, Elke Hartmann^1^, Krystyna Siudak^1^, Alexius Freyberger^1^, Ina Hagelschuer^1^, Ilka Mathar^1^, Joerg Hueser^1^, Michael G. Hahn^1^, Johannes-Peter Stasch^1,4^, Volker Geiss^1^, Frank Eitner^1,5^, Peter Sandner^1,6^

#### ^1^Bayer AG, Research & Development, Pharmaceuticals, Wuppertal North Rhine-Westphalia, Germany; ^2^Vincerx, Leverkusen North Rhine-Westphalia, Germany; ^3^Novo Nordisk, Måløv, Denmark; ^4^University of Halle, Insitute of Pharmacy, Halle Saxony, Germany; ^5^RWTH Aachen, Division of Nephrology and Clinical Immunology, Aachen North Rhine-Westphalia, Germany; ^6^Hannover Medical School, Department of Pharmacology, Hannover Lower Saxony, Germany

##### **Correspondence:** Jan R. Kraehling (jan.kraehling@bayer.com)

*J Transl Med* 2022, **21(1)**:P21

*Published in: Naunyn Schmiedebergs Arch Pharmacol, 2021;394:2363–2379.* https://doi.org/10.1007/s00210-021-02149-4.

## P22 CNP/GC-B-dependent cGMP signalling characterises modulated VSMCs in culture and atherosclerotic arteries

### Moritz Lehners^1^, Maria T. K. Zaldivia^1^, Julia Adler^2^, Robert Lukowski^2^, Hannes Schmidt^1^, Susanne Feil^1^, Robert Feil^1^

#### ^1^University of Tübingen, Interfaculty Institute of Biochemistry, Tübingen Baden-Württemberg, Germany; ^2^University of Tübingen, Department of Pharmacology, Toxicology and Clinical Pharmacy, Institute of Pharmacy, Tübingen Baden-Württemberg, Germany

##### **Correspondence:** Moritz Lehners (moritz.lehners@uni-tuebingen.de)

*J Transl Med* 2022, **21(1)**:P22

**Introduction:** Atherosclerosis and restenosis are vascular diseases that involve remodelling of the arterial vessel wall. Under these conditions, vascular smooth muscle cells (VSMCs) undergo modulation from a contractile towards a synthetic or modulated phenotype. It is widely accepted that VSMCs can generate cGMP upon stimulation with NO, atrial natriuretic peptide (ANP), or C-type natriuretic peptide (CNP), but it is unknown how modulated VSMCs react to these stimuli under pathological conditions. Interestingly, analysis of global and conditional mouse models revealed that cGMP signalling in VSMCs is more important during atherogenesis [1, 2] than restenosis [3]. We hypothesised that cGMP signalling pathways in VSMCs differ between healthy and diseased arteries and that these alterations contribute to VSMC plasticity and thereby to vasculo-proliferative disorders.

**Methods:** We isolated primary VSMCs from mice expressing the cGMP indicator cGi500 [4] to characterise cGMP generation in real-time at the single-cell level in contractile and modulated VSMCs upon application of NO, ANP, and CNP. Furthermore, we analysed the expression of cGMP signalling pathway components using genetic mouse models, single-cell RNA sequencing, and immunostaining. We compared cGMP signals in isolated healthy and pathologically remodelled restenotic and atherosclerotic arteries of cGi500-expressing mice by cGMP imaging.

**Results:** We observed different cGMP signalling pathways in contractile and modulated VSMCs of the same cell culture. The expression of the CNP receptor, guanylyl cyclase-B (GC-B), in VSMCs increased during cell culture. This was accompanied by a shift from ANP/NO-dependent cGMP signalling in contractile VSMCs to CNP-dependent cGMP signalling with attenuated NO responses in modulated VSMCs. These findings were supported by single-cell RNA sequencing data that demonstrated increased levels of GC-B and reduced levels of NO-sensitive guanylyl cyclase in modulated compared to contractile VSMCs. Like in culture, we identified a shift from ANP/NO-dependent cGMP signalling in healthy arteries towards CNP-induced cGMP signalling in atherosclerotic arteries, whereas in restenotic arteries this shift in cGMP signalling was not observed.

**Conclusions:** These results suggest CNP/GC-B-dependent cGMP signalling as a new marker for phenotypically modulated VSMCs in culture and in atherosclerotic aorta, but not restenotic carotid arteries. The “CNP switch” appears to depend on the vascular disease and could explain why mouse studies revealed a role of cGMP in atherosclerosis but not restenosis. Future studies must uncover how CNP-induced cGMP signalling in VSMCs contributes to the pathophysiology of atherosclerosis.

**Acknowledgements:** We thank the Deutsche Forschungsgemeinschaft for the financial support of the Research Training Group “cGMP: From Bedside to Bench” (grant number 335549539/GRK 2381) and the FOR 2060 projects FE 438/5‑2 and FE 438/6‑2.

**References**

1. Wolfsgruber, W., Feil, S., Brummer, S., Kuppinger, O., Hofmann, F., Feil, R. 2003, ‘A proatherogenic role for cGMP-dependent protein kinase in vascular smooth muscle cells.’, *Proc Natl Acad Sci USA, 100* (23), 13519–13524
2. Segura-Puimedon, M., Mergia, E., Al-Hasani, J., Aherrahrou, R., Stoelting, S., Kremer, F., Freyer, J., Koesling, D., Erdmann, J., Schunkert, H., de Wit, C., Aherrahrou, Z. 2016, ‘Proatherosclerotic effect of the alpha1-subunit of soluble guanylyl cyclase by promoting smooth muscle phenotypic switching.’, *Am J Pathol, 186* (8), 2220–2231
3. Lukowski, R., Weinmeister, P., Bernhard, D., Feil, S., Gotthardt, M., Herz, J., Massberg, S., Zernecke, A., Weber, C., Hofmann, F., Feil, R. 2008, ‘Role of smooth muscle cGMP/cGKI signaling in murine vascular restenosis.’, *Arterioscler Thromb Vasc Biol, 28* (7), 1244–1250
4. Thunemann, M., Wen, L., Hillenbrand, M., Vachaviolos, A., Feil, S., Ott, T., Han, X., Fukumura, D., Jain, R. K., Russwurm, M., de Wit, C., Feil, R. 2013, ‘Transgenic mice for cGMP imaging.’, *Circ Res, 113* (4), 365–371

## P23 Heme Redox switch impacts the binding of sGC stimulators to H-NOX domain

### Garyfallia I. Makrynitsa^1^, Aikaterini I. Argyriou^1^, Aikaterini A. Zompra^1^, Konstantinos Salagiannis^3^, Andreas Papapetropoulos^2^, Stavros Topouzis^3^, Georgios A. Spyroulias^1^

#### ^1^University of Patras, Department of Pharmacy, Rio/Patras, Greece; ^2^National and Kapodistrian University of Athens, Department of Pharmacy, Lab. of Pharmacology, Athens, Greece; ^3^University of Patras, Department of Pharmacy, Lab. of Molecular Pharmacology, Rio/Patras, Greece

##### **Correspondence:** Garyfallia I. Makrynitsa (garyfalliamkrn@gmail.com)

*J Transl Med* 2022, **21(1)**:P23

*Published in: Front Cell Dev Biol, 2022;10:925457.* https://doi.org/10.3389/fcell.2022.925457.

**Acknowledgements:** We are indebted to Dr. Peter Sandner (BAYER AG, Wuppertal, Germany) for kindly provided BAY 41-2272. We also acknowledge the Instrumental Analysis Laboratory of University of Patras for the use of AVANCE III, 600 MHz for the acquisition of the 19F 1D spectra. Financial support by the project “INSPIRED” (MIS 5002550, Action “Reinforcement of the Research and Innovation Infrastructure”, NSRF, 2014–2020), Hellenic Foundation for Research and Innovation (HFRI) and the General Secretariat for Research and Technology (GSRT; GA 938) & EU FP7 REGPOT CT-2011-285950—“SEE-DRUG”, is also acknowledged.

## P24 Involvement of cGMP/cAMP crosstalk in proliferation and activation of splenic immune cells

### Evanthia Mergia^1^, Doris Koesling^1^, Marcus Peters^2^

#### ^1^Ruhr-Universität Bochum, Institut für Pharmakologie, Bochum North Rhine-Westphalia, Germany; ^2^Ruhr-Universität Bochum, Abteilung für Molekular Immunologiee, Bochum North Rhine-Westphalia, Germany

##### **Correspondence:** Evanthia Mergia (mergia@outlook.de)

*J Transl Med* 2022, **21(1)**:P24

**Introduction:** In immune cells, cAMP and cGMP signalling have been shown to exert diverse modulatory roles in processes involved in cellular proliferation and in activation/inhibition of the cells. Phosphodiesterase 2 (PDE2), an enzyme that hydrolysis cGMP and cAMP can connect these pathways. Moreover, PDE2 has the unique feature to be activated by cGMP thereby causing a decrease of cAMP concentration.

**Methods:** Here, we examined proliferation and inflammatory responses in splenic immune cells and how they are affected by inducing cGMP (Bay 41 2272, 10 µM) or cAMP signalling (Forskolin 10 µM). Splenic immune cells consist mainly of B- and T-lymphocytes (approx. 60%) and were stimulated with LPS (1 µg/ml, 48 h), an immunogen known to react with the Toll-like receptor 4 (TLR4), but also with the B-cell receptor (BCR) resulting in activation and polyclonal expansion of B-lymphocytes.

**Results:** The cells were found to possess a low cGMP-forming activity by NO-GCs paralleled by a high degradation by PDE2. Activation of NO-GCs resulted in decreased cAMP levels, indicating the existence of an inhibitory cGMP/cAMP crosstalk mechanism via PDE2 in these cells. It is unclear whether this mechanism can play a physiological role, as NO production is required for the crosstalk to be initiated. Under pathophysiological conditions, iNOS can serve as NO source. In splenic immune cells, LPS-stimulation evoked a 30-fold increase in iNOS expression resulting to production of large NO amounts. Further investigation revealed a 1.3-fold increase in cell number that was accompanied by changes in phosphorylation and expression of proteins regulating cell cycle entry and progression. Related to proliferation, enhanced secretion of IgM antibodies and Interleukin-6—needed for B cells maturation—were detected. Of note, cAMP levels were found to be higher in LPS-stimulated cells. Further increase of cAMP levels by Forskolin did not additionally influence the LPS-stimulated cells except of enhancing IL-6 production most likely through activation of the transcription factor CREB (cAMP response element-binding protein) located in IL-6 promoter region. On the other hand, co-administration of Bay 41 resulted in lower cAMP levels and suppressed proliferation and maturation of B cells. The Bay 41 effects were attenuated in cells of NO-GC1 KO mice and were mimicked by the PDE2 activator cBIMP (100 µM).

**Conclusions:** In sum, our results provide evidence that activation of PDE2 can weaken the responses of B-cells to LPS.

## P25 Targeting PDE9 in prostate and epididymis

### Andrea Mietens, Nina Brunner, Jorgos Megalos, Christine Rager, Ralf Middendorff

#### Justus-Liebig-University, Institute of Anatomy and Cell Biology, Gießen Hesse, Germany

##### **Correspondence:** Andrea Mietens (andrea.mietens@anatomie.med.uni-giessen.de)

*J Transl Med* 2022, **21(1)**:P25

**Introduction:** A majority of elderly men suffers from benign prostatic hyperplasia (BPH). PDE5 inhibitors like sildenafil or tadalafil were recently approved to treat lower urinary tract symptoms associated with BPH. Their mechanism of action includes the cGMP-mediated relaxation of prostatic smooth muscle cells (SMCs). In contrast to established treatments with adrenergic blockers, PDE5 inhibitors show no interference with the function of ejaculatory tissues during emission and ejaculation. Thus, they are a valuable therapeutic option in the management of BPH. Specific inhibitors for PDE9, a high affinity cGMP-specific PDE, are currently evaluated as drugs to treat heart failure or neurological conditions and have reached preclinical studies. They may also have potential as drugs for BPH. Our aim was to investigate and localize PDE9 expression in the prostate as well as in the epididymis as an organ relevant to ejaculation.

**Methods:** Western blot and immunofluorescence (IF) used a monoclonal rabbit anti-PDE9 antibody. Comparison to PDE9 KO tissue confirmed the antibody could detect PDE9 in rodent and human tissue. The more detailed cellular localization of PDE9 in prostate and epididymis could be determined by probing membrane and cytosolic protein fractions using Western blot. For IF, prostate and epididymis sections of mouse, rat and men were available. RT-PCR was used to detect PDE9 transcripts in human prostatic SMCs and qPCR served to quantify transcripts in different mouse epididymis regions.

**Results:** PDE9 was found in SMCs in human and rodent prostate, as evidenced by co-localization with the SMC marker smooth muscle actin (SMA) in IF, it was also detected in vascular SMCs. PDE9 could be assigned to the membrane fraction in rat prostate by Western blot. The SMC localization of PDE9 was confirmed by RT-PCR in isolated human prostatic SMCs. Epididymis tissue revealed a staining pattern for PDE9 comparable to the prostate with a signal in the smooth muscle layer surrounding the epididymal duct and vascular SMCs. qPCR data showed a higher expression of PDE9 in the cauda epididymis.

**Conclusions:** Besides its expression in the epididymis, the SMC localization of PDE9 in the prostate identifies this PDE as a potential therapeutic target in the treatment of BPH. Its membrane localization in these organs supports the idea of interactions of PDE9 with distinct subcellular, e.g. natriuretic peptide-dependent, pools of cGMP.

## P26 Brain-penetrant sGC stimulator BAY-747 and activator runcaciguat act as cognitive enhancers via differential neuroplastic mechanisms

### Ellis Nelissen^1^, Tim Vanmierlo^1,2^, Peter Sandner^3,4^, Jos Prickaerts^1^

#### ^1^Maastricht University, Psychiatry and Neuropsychology, Maastricht, Netherlands; ^2^Hasselt University, Biomedical Research Institute, Hasselt, Belgium; ^3^Bayer AG, Cardiovascular Research, Wuppertal, Germany; ^4^Hannover Medical School, Hannover, Germany

##### **Correspondence:** Ellis Nelissen (e.nelissen@maastrichtuniversity.nl)

*J Transl Med* 2022, **21(1)**:P26

**Introduction:** Soluble guanylyl cyclase (sGC)-derived cGMP signaling plays a role in both neurons and the vasculature: neuronal cGMP signaling is involved in memory formation, while endothelial cGMP signaling plays a role in vascular health. Therefore, sGC may be a target to treat vascular cognitive impairment (VCI).

sGC can be targeted by stimulators acting on heme-bound sGC or by activators acting on oxidized/apo-sGC. However, first generations of sGC agonists do not cross the blood–brain barrier, potentially limiting the neuronal therapeutic effectiveness. Here, we compare novel brain-penetrant sGC stimulator BAY-747 and sGC activator runcaciguat for their potential as cognitive enhancers.

**Methods:** BAY-747 and runcaciguat were tested in vivo in the object location task (OLT) in rodents to assess their potential as cognitive enhancers. In addition, a NOS-inhibitor L-NAME-induced memory deficit model was used to elucidate the mechanistic differences. In vivo effects on neuroplasticity were also measured.

**Results:** Both BAY-747 and runcaciguat enhanced long-term memory in a natural forgetting model of the OLT. Interestingly, only BAY-747 attenuated the memory impairments induced by NOS-inhibitor L-NAME. This shows that sGC stimulators and activators may differ in efficacy depending on the underlying mechanism of memory impairment.

The neuroplastic effects underlying cognitive enhancement by BAY-747 and runcaciguat were assessed in vivo. While runcaciguat was more effective on the glutamatergic system, BAY-747 more strongly enhanced the neurotrophic system.

**Conclusions:** These data show that brain-penetrant sGC agonists act as cognitive enhancers via neuroplastic effects in vivo. Based on the underlying memory deficit mechanism, sGC stimulators and activators may differ in efficacy. Additionally, while sGC stimulator BAY-747 acts on the neurotrophic system, sGC activator runcaciguat more strongly enhances the glutamatergic system. This further indicates differential effects for sGC stimulators versus sGC activators. Interestingly, BAY-747 and runcaciguat are effective in a similar dose–response window, while BAY-747 shows a larger brain penetration rate (38–60% vs 10–13%). This suggests that pharmacokinetics and enzyme binding kinetics differ between BAY-747 and runcaciguat, which may influence the specific neuroplastic and cognition enhancing effects. Therefore, conclusions on superiority of one sGC agonist over the other cannot be drawn. Nevertheless, brain-penetrant sGC agonists show promise as cognitive enhancers with clear neuronal effects.

**Funding:** Funding was provided by a restricted research grant from Bayer AG, Pharmaceuticals.

## P27 Inhibitory effects of C-type natriuretic peptide on PDGF-BB induced metabolic remodelling of lung pericytes from patients with pulmonary hypertension

### Minhee Noh^1^, Lisa Krebes^1^, Werner Schmitz^2^, Jan Dudek^3^, Christoph Maack^3^, Vinicio de Jesus Perez^4^, Hannes Schmidt^5^, Swati Dabral^1^, Michaela Kuhn^1,3^

#### ^1^University of Würzburg, Institute of Physiology, Würzburg Bavaria, Germany; ^2^University of Würzburg, Institute of Biochemistry and Molecular Biology, Würzburg Bavaria, Germany; ^3^University Hospital Würzburg, Comprehensive Heart Failure Center (CHFC), Würzburg Bavaria, Germany; ^4^Stanford University, Divisions of Pulmonary and Critical Care Medicine and Stanford Cardiovascular Institute, Stanford California, USA; ^5^University of Tübingen, Interfaculty Institute of Biochemistry, Tübingen Baden-Württemberg, Germany

##### **Correspondence:** Minhee Noh (minhee.noh@uni-wuerzburg.de)

*J Transl Med* 2022, **21(1)**:P27.

*Swati Dabral and Michaela Kuhn contributed equally*

**Introduction:** Pericytes are mural cells regulating the perfusion and barrier functions of the systemic and pulmonary microcirculation. A hyperproliferative phenotype and metabolic switch to higher glycolysis rates contribute to vascular pathologies such as pulmonary hypertension (PH) (1). Increased expression of growth factors such as platelet-derived growth factor-BB (PDGF-BB) has been implicated in the initiation and progression of these pathological alterations. We studied the effect of the endothelial hormone C-type natriuretic peptide (CNP), signalling through the cyclic GMP-producing guanylyl cyclase B (GC-B) receptor (2), on the PDGF-BB‑induced hyperproliferative and metabolic switch of cultured human lung pericytes from control donors and patients with PH (1).

**Methods:** See below.

**Results:** CNP (0.01–100 nM) markedly and similarly increased intracellular cGMP levels in control and PH pericytes, demonstrating CNP/GC-B/cGMP signalling. These cGMP responses were not altered by PDGF-BB pretreatment (30 ng/ml, 30 min). Upon PDGF-BB stimulation, PH pericytes exhibited higher proliferation coupled with enhanced rate of glycolysis in comparison to control pericytes. Notably, these effects were significantly attenuated by CNP. Mass spectrometry-based metabolomic analyses revealed an upregulation of glycolytic intermediates in response to PDGF-BB, which was prevented by CNP. To dissect the underlying mechanisms, the expression levels of various components of the glycolytic pathway as well as of their well-known regulator hypoxia-inducible factor 1 alpha (HIF-1α) were analysed by immunoblotting. PDGF-BB markedly upregulated membrane glucose transporter 1 (GLUT1) and nuclear HIF-1α levels, and CNP prevented these alterations in both control and PH pericytes.

**Conclusions:** Taken together, our data show that CNP attenuates both the PDGF-BB-induced hyperproliferation and the increased glycolysis rate of human PH lung pericytes. Mechanistically, the protective actions of CNP are partly mediated by inhibition of HIF-1α-dependent GLUT1 induction. This results in reduction of cellular glucose uptake and concomitant glycolytic flux. To further characterize these findings, we are studying the downstream signalling pathways involved in CNP/GC-B mediated HIF-1α and GLUT1 regulation as well as glucose uptake of cultured lung pericytes. Understanding the effects and signalling pathways of CNP in pericytes may unravel novel targets for therapies of PH.

**Funding:** Supported by the Deutsche Forschungsgemeinschaft (DFG KU 1037/12-1 and DFG DA 2462/1-1).

**References**

1. Yuan K, et al. 2016, ‘Increased Pyruvate Dehydrogenase Kinase 4 Expression in Lung Pericytes Is Associated with Reduced Endothelial-Pericyte Interactions and Small Vessel Loss in Pulmonary Arterial Hypertension’, *Am J Pathol*, 186, 2500–14.
2. Kuhn M, 2016, ‘Molecular Physiology of Membrane Guanylyl Cyclase Receptors’, *Physiol Rev*, 96, 751–804

## P28 Monitoring oxidation of cGMP-dependent protein kinase Iα by nitroxyl donors

### Julia Pflaumenbaum^1,2^, Fabian Gutknecht^1,2^, Konstantina Stathopoulou^1,2^, Angelika Piasecki^1,2^, Viacheslav Nikolaev^2,3^, Friederike Cuello^1,2^

#### ^1^University Medical Center Hamburg-Eppendorf, Institute of Experimental Pharmacology and Toxicology, Hamburg, Germany; ^2^DZHK (German Center for Cardiovascular Research), Partner site Hamburg/Kiel/Lübeck, Hamburg, Germany; ^3^University Medical Center Hamburg-Eppendorf, Institute of Experimental Cardiovascular Research, Hamburg, Germany

##### **Correspondence:** Julia Pflaumenbaum (j.pflaumenbaum@uke.de)

*J Transl Med* 2022, **21(1)**:P28

**Introduction:** cGMP-dependent protein kinase Iα (PKGIα) is one of the main regulators of vascular relaxation. PKGIα activation occurs for example in response to nitric oxide (NO) and subsequent generation of cGMP by the soluble guanylyl cyclase (sGC). Clinically, NO donors lower blood pressure via this mechanism but often lead to the development of drug tolerance. Therefore, the one electron reduced and protonated sibling of NO, nitroxyl (HNO), represents a promising therapeutic alternative for vasodilation without inducing tolerance. Recently, we have shown that HNO induces oxidation-mediated intradisulfide bond formation between cysteines 117 and 195 in the high affinity cGMP-binding domain of PKGIα. However, the individual contributions of these cysteines to the cGMP-independent vasorelaxation are still debated. Here, we aimed to address this question by measuring HNO-mediated changes in a set of newly generated Förster resonance energy transfer (FRET) based biosensors.

**Methods:** We performed automated real time monitoring of the HNO induced conformational changes in the cGMP binding domain of PKGIα using the wildtype (WT) cGi500 biosensor and the mutants C117S, C195S and C117/195S cGi500, which were stably expressed in HEK293 cells. In vitro kinase assays were used to measure HNO induced PKGIα activity.

**Results:** HNO donor exposure (CXL-1020, 300 µM; 1-nitrosocyclohexyl acetate, NCA, 100 µM), similarly to the NO donor sodium nitroprusside (50 µM), led to a quick and robust change of the FRET response. Unexpectedly, these responses were similar for all constructs unless sGC was inhibited by ODQ, suggesting direct sGC activation by HNO. The HNO donor responses measured in the presence of the sGC inhibitor ODQ were reduced by ~ 15% in cGi500 C117S, ~ 47% in cGi500 C195S and by ~ 67% in the double sensor mutant cGi500 C117/195S, when compared to the WT sensor cGi500. In vitro kinase assays showed that WT PKG significantly increased substrate phosphorylation in response to HNO exposure. Substrate phosphorylation was significantly reduced when experiments were performed with C117S (by ~ 60%), C195S (by ~ 75%) or the double mutant C117/195S (by ~ 83%) PKGIα.

**Conclusions:** NCA and CXL-1020 are able to stimulate sGC, resulting in cGMP production and sensor activation. When sGC is inhibited, HNO donor exposure leads to FRET responses by inducing a conformational change in the sensor that is mediated via oxidative modification of C117 and C195 in the high affinity cGMP binding site of cGi500, similar to that induced by cGMP-binding. This correlates with HNO-induced direct PKGIα activation as evidenced by increased substrate phosphorylation.

## P29 cGKI/BK interactions may be crucial for Schaffer collateral LTP and hippocampus-dependent memory formation

### Thomas Pham, Tamara Hussein, Helmut Bischof, Stefanie Simonsig, Daniel Kalina, Rebekka Ehinger, Peter Ruth, Robert Lukowski, Lucas Matt

#### University of Tübingen, Pharmacology, Toxicology and Clinical Pharmacy, Institute of Pharmacy, Tübingen, Germany

##### **Correspondence:** Thomas Pham (thomas.pham@uni-tuebingen.de)

*J Transl Med* 2022, **21(1)**:P29

**Introduction:** Mutations of the Ca^2+^- and voltage-activated channel BK are associated with cognitive decline emerging from impaired synaptic plasticity in neurodegenerative diseases [1]. BK’s role in synaptic plasticity, however, is not fully understood yet. Interestingly, mouse models of cognitive impairments could be rescued by activation of the NO-GC/cGMP pathway. Since BK is an established cGMP/cGKI substrate, it might mediate some of cGKI’s beneficial effects on cognitive function [2]. Due to cerebellar ataxia observed in globally BK-deficient mice [3] we used CA1 pyramidal neuron-specific knockout mice (CA1-BK-KO) and littermate controls to examine the role of cGKI and BK in synaptic plasticity and cognitive function.

**Methods:** Fear behavior and locomotor activity were assessed in open-field and beam-walk tests, respectively. Memory acquisition and retrieval was evaluated in the Morris Water Maze (MWM). Long-term potentiation (LTP) was investigated in acute brain slices, as well as phosphorylation of the AMPA receptor (AMPAR) subunit GluA1, a surrogate parameter for physiological LTP, after chemically induced LTP (cLTP). Intracellular potassium levels ([K^+^]_i_) and Ca^2+^ dynamics were measured in primary hippocampal neuron cultures using a genetically encoded K^+^ indicator (GEPII) [4, 5] and a Ca^2+^-sensitive fluorescent dye (Fura-2AM).

**Results:** Immunoblot and -fluorescence demonstrated effective and specific BK depletion. Open-field and beam-walk tests verified CA1-BK-KO’s suitability for behavior-based memory tasks as fear responses and locomotor activity did not differ between genotypes. In the MWM, CA1-BK-KO did not develop a target-oriented search strategy and failed during memory retrieval, indicating impaired hippocampus-dependent memory acquisition. These findings were further corroborated in CA1-BK-KO brain slices by the lack of electrically inducible LTP and the absence of increased GluA1 Ser845 phosphorylation after cLTP. In primary hippocampal neurons, GEPII revealed a massive BK-mediated reduction in [K^+^]_i_ after cLTP induction. This decrease in [K^+^]_i_, was accompanied by elevated frequencies of neuronal Ca^2+^ oscillations. Both K^+^ efflux and sustained Ca^2+^ oscillations during cLTP induction were abrogated by AP5, a selective NMDA receptor antagonist, or Nifedipine while Cinaciguat augmented Ca^2+^ oscillations during cLTP.

**Conclusions:** We propose that BK-mediated K^+^ efflux substantially contributes to hippocampal synaptic plasticity by maintaining NMDA receptor and L-Type Ca^2+^ channel-dependent oscillatory Ca^2+^ influx during cLTP. This suggests BK modulation, possibly through cGMP/cGKI, as a potential therapeutic option for the treatment of impaired cognition.

**Funding:** This project was funded by the Deutsche Forschungsgemeinschaft (DFG, German Research Foundation)—Project numbers 335549539/GRK2381 and 445878942.

**References**

1. Du, X., Carvalho-de-Souza, J. L., Wei, C., Carrasquel-Ursulaez, W., Lorenzo, Y., Gonzalez, N., Kubota, T., Staisch, J., Hain, T., Petrossian, N., Xu, M., Latorre, R., Bezanilla, F., & Gomez, C. M. (2020). Loss-of-function BK channel mutation causes impaired mitochondria and progressive cerebellar ataxia. *Proc Natl Acad Sci USA*, *117*(11), 6023–6034. https://doi.org/10.1073/pnas.1920008117
2. Prieto, G. A., Trieu, B. H., Dang, C. T., Bilousova, T., Gylys, K. H., Berchtold, N. C., Lynch, G., & Cotman, C. W. (2017). Pharmacological Rescue of Long-Term Potentiation in Alzheimer Diseased Synapses. *J Neurosci*, *37*(5), 1197–1212. https://doi.org/10.1523/JNEUROSCI.2774-16.2016
3. Sausbier, M., Hu, H., Arntz, C., Feil, S., Kamm, S., Adelsberger, H., Sausbier, U., Sailer, C. A., Feil, R., Hofmann, F., Korth, M., Shipston, M. J., Knaus, H. G., Wolfer, D. P., Pedroarena, C. M., Storm, J. F., & Ruth, P. (2004). Cerebellar ataxia and Purkinje cell dysfunction caused by Ca2^+^-activated K+ channel deficiency. *Proc Natl Acad Sci USA*, *101*(25), 9474–9478. https://doi.org/10.1073/pnas.0401702101
4. Bischof, H., Rehberg, M., Stryeck, S., Artinger, K., Eroglu, E., Waldeck-Weiermair, M., Gottschalk, B., Rost, R., Deak, A. T., Niedrist, T., Vujic, N., Lindermuth, H., Prassl, R., Pelzmann, B., Groschner, K., Kratky, D., Eller, K., Rosenkranz, A. R., Madl, T., Plesnila, N., Graier, W. F., & Malli, R. (2017). Novel genetically encoded fluorescent probes enable real-time detection of potassium in vitro and in vivo. *Nat Commun*, *8*(1), 1422. https://doi.org/10.1038/s41467-017-01615-z
5. Ehinger, R., Kuret, A., Matt, L., Frank, N., Wild, K., Kabagema-Bilan, C., Bischof, H., Malli, R., Ruth, P., Bausch, A. E., & Lukowski, R. (2021). Slack K(+) channels attenuate NMDA-induced excitotoxic brain damage and neuronal cell death. *FASEB J*, *35*(5), e21568. https://doi.org/10.1096/fj.202002308RR

## P30 The natriuretic peptide receptors in vascular smooth muscle cells: cell culture the approach of choice?

### Christine Rager, Tobias Klöpper, Andrea Mietens, Ralf Middendorff

#### Justus-Liebig-University, Institute of Anatomy and Cell Biology, 35392 Hesse, Germany

##### **Correspondence:** Christine Rager (christine.rager@anatomie.med.uni-giessen.de)

*J Transl Med* 2022, **21(1)**:P30

**Introduction:** The natriuretic peptides (NPs) ANP and BNP, produced in the heart, induce vasodilation in arterial vessels. They bind to a specific receptor GC-A. CNP is associated with long-bone growth and oocyte maturation but also affects vascular function. CNP binds to the receptor GC-B. In addition, all NPs are bound by the clearance receptor (CR). Numerous data on vascular smooth muscle cells (SMCs) is based on experiments with cultured cells. Aim of this study was to investigate whether culturing of primary vascular SMCs results in a subtype switching of the NP receptors compared to intact vascular tissue. Therefore, aorta with its thick SM layer (tunica media) was selected as model vessel.

**Methods:** Rodent aortae of both genders were prepared. Outer connective tissue (tunica adventitia) and inner endothelium (tunica intima) were removed. One half of the tissue was directly used for experiments whereas the other half was used to isolate SMCs. Therefore, an enzymatic digest was performed, and cells were separated through a cell strainer. Isolated SMCs were identified by SM actin and calponin expression using immunofluorescence, and contamination with endothelial cells could be excluded after negative staining for endothelial markers. NP receptors’ expression was determined via RT-qPCR in tissue and cells, the latter right after extraction and in the first passage after extraction (approx. 10 days of culturing). Moreover, functional activity of GC-A and GC-B was revealed by ANP and CNP induced cGMP production measured by ELISA again in intact tissue and cultured cells.

**Results:** Within the intact vessel wall GC-A was found to be expressed higher than GC-B, whereas in cultured SMCs it was vice versa for both genders. Also, the expression of other key players within the NP/cGMP system showed an altered expression pattern in cell culture. The CR for instance was expressed significantly higher in cultured SMCs of female rats in comparison to the intact tissue. The expression of the soluble guanylyl cyclase was significantly lower in the cultured SMCs of both genders. In addition, the switch in GC-A and GC-B expression was confirmed functionally by ELISA. Here the ANP-treatment of intact tissue resulted in a higher cGMP release, whereas in the corresponding primary cells treated with CNP the cGMP level was higher.

**Conclusions:** Our data shows that culturing affects the expression of NP receptors and cGMP related genes in cultured vascular SMCs. Hence, in vivo or total tissue approaches might reflect NP related signalling more realistically. A reason for this could be a general expression switch of all SMCs or the predominance of a SMC subpopulation.

## P31 Effects of uroguanylin’s GC-C/cGMP signalling pathway on ischemic stroke

### Martina Ratko^1,2^, Nikola Habek^1,3^, Marina Dobrivojević Radmilović^1^, Siniša Škokić^1^, Helena Justić^1^, Anja Barić^1^, Aleksandra Dugandžić^1,3^

#### ^1^School of Medicine, University of Zagreb, Croatian Institute for Brain Research, Zagreb, Croatia; ^2^School of Medicine, University of Zagreb, Centre of Excellence for Basic, Clinical and Translational Neuroscience, Zagreb, Croatia; ^3^School of Medicine, University of Zagreb, Department of Physiology, Zagreb, Croatia

##### **Correspondence:** Martina Ratko (martina.ratko@mef.hr)

*J Transl Med* 2022, **21(1)**:P31

**Introduction:** Stroke is one of the leading causes of mortality and disability in industrialized countries. Guanylate cyclase (GC) A activation after ischemic stroke is neuroprotective [1]. The aim of this study is to investigate what effect GC-C activation by uroguanylin (UGN) has on ischemic stroke.

**Methods:** Middle cerebral artery occlusion (MCAO) was performed on GC-C knock out (GC-C KO), UGN KO mice and their WT littermates. MR images were acquired before and 24 h after stroke induction. Ca^2+^ response was recorded in astrocytes of peri-ischemic and contralateral cortices 48 h after MCAO. Systolic (SP), diastolic (DP) and mean arterial blood pressure (MAP), heart rate, tail blood volume and flow were recorded with a non-invasive tail-cuff method.

**Results:** Animals lacking GC-C develop significantly smaller ischemic lesions compared to their WT littermates. Though GC-C KO mice have higher SP, DP and MAP, blood flow or the reduction of blood flow during MCAO did not differ between KO and WT animals. UGN KO and WT mice have a stronger Ca^2+^ response to UGN stimulation in cortical peri-ischemic astrocytes compared to the healthy hemisphere while this stronger activation is absent in GC-C KO mice. The observed difference in response may result in smaller ischemic lesions observed in GC-C KO mice. This was explained when immunohistochemical staining showed GC-C expression in peri-ischemic astrocytes, while under normoxic conditions GC-C expression is present only in neurons [2].

**Conclusions:** Contrary to GC-A, GC-C activation is not neuroprotective but instead leads to the development of larger ischemic lesions. Its expression in peri-ischemic astrocytes causes a stronger activation of the Ca^2+^-dependent signalling pathway which could stimulate the Na^+^/H^+^ exchanger causing tissue acidification and neuronal death.

**Funding:** Research was funded by the Scientific Centre of Excellence for Basic, Clinical and Translational Neuroscience (project “Experimental and clinical research of hypoxic-ischemic damage in perinatal and adult brain”; GA KK01.1.1.01.0007 funded by the European Union through the European Regional Development Fund). The work of doctoral student Anja Barić has been fully supported by the “Young researchers’ career development project—training of doctoral students” and project BRADISCHEMIA (UIP-2017-05-8082) of the Croatian Science Foundation funded by the European Union from the European Social Fund.

**References**

1. Dobrivojević, M, Špiranec, K, Gorup, D, Erjavec, I, Habek, N, Radmilović, M, Unfirer, S, Ćosić, A, Drenjančević, I, Gajović, S, Sinđić, A 2016, ‘Urodilatin reverses the detrimental influence of bradykinin in acute ischemic stroke’, *Exp Neurol,* 284, 1–10.
2. Habek, N, Ratko, M, Dugandžić, A 2021, ‘Uroguanylin increases Ca2+ concentration in astrocytes via guanylate cyclase C-independent signaling pathway’, *Croat Med J,* 62, 250–263.

## P32 Effects of NO-GC stimulators and activators on platelet biomechanics

### Johanna G. Rodríguez^1^, Aylin Balmes^1^, Jan Seifert^1^, Daniel Pinto^2^, Andreas Friebe^3^, Francesca Seta^4^, Robert Feil^2^, Sussanne Feil^2^, Tilman E. Schäffer^1^

#### ^1^University of Tübingen, Institute of Applied Physics, Tübingen, Germany; ^2^University of Tübingen, Interfakultäres Institut für Biochemie (IFIB), Tübingen, Germany; ^3^Julius Maximilian University of Würzburg, Physiological Institute, Würzburg, Germany; ^4^Boston University School of Medicine, Vascular Biology Section, Boston, USA

##### **Correspondence:** Johanna G. Rodríguez (johanna.rodriguez@uni-tuebingen.de)

*J Transl Med* 2022, **21(1)**:P32

**Introduction:** Cyclic guanosine monophosphate (cGMP) is a second messenger produced by the enzyme guanylyl cyclase (GC) in response to nitric oxide (NO). NO-sensitive GC (NO-GC) is expressed in a variety of cell types including smooth muscle cells and platelets. In platelets, the NO-GC/cGMP pathway is thought to inhibit platelet aggregation via changes of the cytoskeleton. However, the molecular mechanism underlying platelet inhibition and its correlation with cytoskeleton stiffness is poorly understood.

**Methods:** To study the role of NO-GC/cGMP in platelet stiffness and inhibition, we applied scanning ion conductance microscopy (SICM), a nanopipette-based noncontact imaging method, to resolve the morphology and stiffness of single platelets at high spatial resolution. We quantified morphological changes in platelets using a deep learning convolutional neural network. Actin polymerization and platelet activation were measured by F-actin and P-selectin co-staining, respectively.

**Results:** We found that stimulation of human and murine platelets with the NO-GC stimulator Riociguat (10 μM, ten minutes) or with the NO-GC activator Cinaciguat (10 μM, ten minutes) downregulated P-selectin expression and decreased stiffness by fifty percent compared to vehicle (DMSO) treated control. Also, the platelet shape became more round, while the platelet area was unaffected. F-actin polymerization was inhibited by fifty percent by Riociguat and Cinaciguat.

**Conclusions:** These observations can be linked to the decreased stiffness of the drug-treated platelets. Remarkably, none of these changes were observed in platelets isolated from platelet-specific NO-GC knockout mice,

demonstrating a functional role of the NO-GC/cGMP pathway and the feasibility of its pharmacological targeting to modulate platelet cytoskeleton biomechanics.

**Funding:** This project is funded by the Deutsche Forschunggemeinschaft (DFG, German Research Foundation-Projektnummer 335549539/GRK2381).

## P33 Real-time analysis and therapeutic potential of NO/cGMP signaling in VSMCs and atherosclerosis

### Malte Roessing^1^, Moritz Lehners^1^, Anastasia Gurskaya^1^, Peter Sandner^2,3^, Robert Feil^1^, Susanne Feil^1^

#### ^1^University of Tübingen, Interfaculty Institute of Biochemistry, Tübingen, Germany; ^2^Bayer AG, Pharma Research Center, Wuppertal, Germany; ^3^Hannover Medical School, Department of Pharmacology, Hannover, Germany

##### **Correspondence:** Malte Roessing (malte.roessing@uni-tuebingen.de)

*J Transl Med* 2022, **21(1)**:P33

**Introduction:** Vascular smooth muscle cells (VSMCs) play a key role in atherosclerosis [1]. They may contribute to the formation of atherosclerotic plaques through migration, clonal expansion, and transdifferentiation [1, 2]. The molecular mechanisms leading to the alteration of VSMC behavior in atherogenesis are not fully understood. It is known that activation of NO-sensitive guanylyl cyclase (NO-GC) in VSMCs leads to an intracellular increase in cyclic guanosine monophosphate (cGMP) and subsequent vascular relaxation [3]. Drugs modulating the NO-GC/cGMP pathway could influence the behavior and phenotype of VSMCs but have not been extensively studied in atherogenesis. We hypothesize that modulation of the NO/cGMP pathway alters transdifferentiation of VSMCs in atherosclerosis.

**Methods:** cGMP signals were visualized in real-time in isolated primary VSMCs and aortic ex vivo preparations from transgenic mice expressing the cGMP sensor cGi500 [4]. Effects of NO-GC modulating drugs on cGMP levels and on the growth of VSMCs were monitored in real-time. To determine NO-GC expression, immunofluorescence staining with a NO‑GC-specific antibody was performed on aortic sections of atherosclerotic mice. The smooth muscle origin of immunostained plaque cells was validated using sections from smooth muscle lineage tracing mice.

**Results:** In cultured primary VSMCs and in isolated aortic preparations measurements of cGMP showed a concentration-dependent increase in response to the NO donor DEA/NO as well as to NO-GC modulating drugs like NO-GC stimulators (Vericiguat, BAY NO-GC stimulator tool compound) or NO-GC activator (BAY NO-GC activator tool compound). The cGMP increases correlated with growth-promoting effects of the tested agents in primary VSMCs. The combination of NO-GC stimulators with the NO donor potentiated the cGMP signals and VSMC growth in comparison to NO donor or drug alone. Potential target cells for NO-GC modulating drugs were determined via NO-GC immunostaining. In atherosclerotic plaques NO-GC expression was detected in medial, fibrous cap and core region cells, which could be identified as SMCs or smooth muscle cell-derived cells.

**Conclusions:** Taken together, these results suggest that NO-GC modulating drugs enhance cGMP signaling and thereby promote the growth of VSMCs. Future in vivo studies are needed to investigate whether NO-GC modulators have pro- or anti-atherogenic effects.

**Acknowledgements:** We thank the Deutsche Forschungsgemeinschaft for the financial support of the Research Training Group “cGMP: From Bedside to Bench” (grant number 335549539/GRK 2381), ERA-CVD JTC2017-044 SCAN for funding and the BAYER AG, Pharmaceuticals for providing the BAY NO-GC stimulator and NO-GC activator tool compounds.

**References**

1. Basatemur, G.L., Jorgensen, H.F., Clarke, M.C.H., Bennett, M.R., and Mallat, Z., 2019, Vascular smooth muscle cells in atherosclerosis, Nature Reviews Cardiology, 16(12), 727–744, https://doi.org/10.1038/s41569-019-0227-9.
2. Feil, S., Fehrenbacher, B., Lukowski, R., Essmann, F., Schulze-Osthoff, K., Schaller, M., and Feil, R., 2014, Transdifferentiation of vascular smooth muscle cells to macrophage-like cells during atherogenesis, Circulation Research, 115(7), 662–667, https://doi.org/10.1161/CIRCRESAHA.115.304634.
3. Murad, F., 2006, Shattuck Lecture. Nitric oxide and cyclic GMP in cell signaling and drug development, The New England Journal of Medicine, 355(19), 2003–11, https://doi.org/10.1056/NEJMsa063904.
4. Thunemann, M., Wen, L., Hillenbrand, L., Vachaviolos, A., Feil, S., Ott, T., Han, X., Fukumura, D., Jain, R.K., Russwurm, M, de Wit, C., Feil, R., 2013, Transgenic mice for cGMP imaging, Circulation Research, 113(4), 365–71, https://doi.org/10.1161/CIRCRESAHA.113.301063.

## P34 NO-induced cGMP generated in cardiac fibroblasts increases cardiac cAMP and phospholamban phosphorylation via inhibition of PDE3

### Doris Koesling, Lukas Menges, Jan Giesen, Michael Russwurm

#### Ruhr-Universität Bochum, Pharmakologie, Bochum, Germany

##### **Correspondence:** Michael Russwurm (michael.russwurm@ruhr-uni-bochum.de)

*J Transl Med* 2022, **21(1)**:P34

**Introduction:** Whether NO can induce cGMP signals in cardiomyocytes has been a matter of debate. In our hands, NO-induced cGMP signals are undetectable in isolated adult cardiac myocytes using a FRET-based cGMP indicator. In contrast, cardiac fibroblasts display substantial NO-induced cGMP increases. In a fibroblast/myocyte co-culture model, we were able to demonstrate transfer of NO-induced cGMP from fibroblasts to cardiac myocytes via gap junctions.

**Methods:** Resident cardiac fibroblasts are characterised by expression of Tcf21. Cardiac Slices of mice expressing a FRET-based cGMP indicator either specifically in Tcf21-positive fibroblasts or specifically in cardiac myocytes were used to analyse cGMP signals in fibroblasts and cardiac myocytes, respectively.

**Results:** In cardiac slices of mice expressing a cGMP indicator specifically in fibroblasts, we demonstrate that resident fibroblasts in native cardiac tissue indeed display cGMP signals brought about by stimulation of NO-sensitive guanylyl cyclase. Furthermore, using mice with cardiac myocyte-specific expression of a FRET-based cGMP indicator, we demonstrate gap junction-mediated cGMP transfer from cardiac fibroblasts to myocytes in intact cardiac tissue. Because transferred NO-induced cGMP was mainly under control of PDE3, we speculated about a cross talk between cGMP and cAMP via PDE3. Indeed, stimulation of NO-sensitive guanylyl cyclase enhanced Forskolin- and Isoproterenol-induced cAMP which was accompanied by enhanced phospholamban phosphorylation.

**Conclusions:** Thus, in native cardiac tissue, heterocellular coupling of fibroblasts and myocytes allows fibroblast-generated, NO-induced cGMP to enter cardiac myocytes. Here, the resulting reduction of PDE3-mediated cAMP degradation amplifies β receptor-induced cAMP and downstream events. Hence, enhancement of the β receptor-induced contractile response appears as one of NO/cGMP’s functions in the non-failing heart.

## P35 Functional characterization in vitro of new multi-substituted 1*H*-pyrazolo[3,4-c]pyridin-7(6*H*)-ones as soluble guanylyl cyclase (sGC) stimulators

### Dionysios Kintos^1^, Konstantinos Salagiannis^2^, Sotirios Nikolaropoulos^1^, Manolis Fousteris^1^, Antonios Sgouros^1^, Stavros Topouzis^2^

#### ^1^University of Patras, Department of Pharmacy, Lab. of Pharmaceutical Chemistry, Rio/Patrras, Greece; ^2^University of Patras, Department of Pharmacy, Lab. of Molecular Pharmacology, Rio/Patras, Greece

##### **Correspondence:** Konstantinos Salagiannis (salas.kons21@gmail.com)

*J Transl Med* 2022, **21(1)**:P35

**Introduction:** The NO-sGC-cGMP axis regulates multiple functions in the cardiovascular system. Its dysfunction in humans contributes to the development of cardiorenal and cardiopulmonary disorders. For this reason, its pivotal component, sGC, is a prime therapeutic target for the development of novel sGC agonists.

**Methods:** New sGC stimulator molecules were designed by a rational design process involving analysis of the structural features of known sGC-stimulator scaffolds and their binding at the H-NOX of cyanobacterium *Nostoc* sp., synthesized and purified. BAY 41-2272, which exhibits the typical profile of a potent and selective sGC stimulator, was used as a reference tool, throughout these studies.

The ability to stimulate sGC and their mechanism of action were studied in vitro in A7r5 rat aortic smooth muscle cells by ELISA immunoassay (cGMP levels) and by Western blotting (detection of VASP protein phosphorylation at Ser_239_). Modulation of cell function was characterized by assessing their effect on the proliferation of A7r5, using an MTT assay. Subsequently, the evaluation of selected compounds to mitigate the inflammatory effects of TNFα and IL-1β was tested in primary endothelial cells (HUVECs) by (a) the detection of ICAM-1 and P/E-Selectin surface adhesion molecules by ELISA and (b) by the measurement of U-937 leukocyte adhesion.

**Results:** The novel molecules do not increase cGMP by themselves in A7r5 cells but are able to elicit cGMP increases comparable with those seen with the NO donor sodium nitroprusside (SNP) in HUVECs. In addition, they strongly synergize with SNP, inducing cGMP synthesis comparable to that observed by BAY 41-2272 in both cell types; these effects are inhibited by oxidation of the heme by ODQ, suggesting the typical mechanism of action of sGC “stimulators”. Moreover, these molecules are capable of inducing VASP phosphorylation at Ser_239_ in A7r5 cells either alone or in synergy with SNP, indicating the engagement of the downstream cGMP-dependent protein kinase, PKG.

Furthermore, these compounds display functional responses characteristic of similar sGC agonists: compound DPK-382 reduces the proliferation of A7r5 cells, whereas compound DPK-384 dampens the expression of ICAM-1 and P/E-Selectin on the surface of HUVECs as well as the adherence of U937 cells to their surface, in response to TNFα and IL-1β.

**Conclusions:** In summary, we describe the characterization novel 1*H*-pyrazolo[3,4-c]pyridin-7(6*H*)-one derivatives as functional heme-dependent, NO-independent and NO-synergizing sGC stimulators. These results pave the way for further investigation of their therapeutic potential and optimization of this class of compounds.

**Funding:** BAY 41-2272 was kindly provided by Dr. Peter Sandner (Bayer AG, Wuppertal, Germany). We acknowledge support of this work by the project “INSPIRED-The National Research Infrastructures on Integrated Structural Biology, Drug Screening Efforts and Drug target functional characterization” (MIS 5002550) which is implemented under the Action “Reinforcement of the Research and Innovation Infrastructure”, funded by the Operational Programme “Competitiveness, Entrepreneurship and Innovation” (NSRF, 2014–2020) and co-financed by Greece and the European Union (European Regional Development Fund). We also acknowledge the Instrumental Analysis Laboratory of University of Patras for the use of AVANCE III, 600 MHz for the acquisition of the ^19^F 1D spectra.

## P36 Modulating enzymatic activity of the promiscuous nucleotidyl cyclase ExoY of *Pseudomonas aeruginosa*

### Bastian Schirmer, Solveig Kälble, Jannik Boog, Roland Seifert

#### Hannover Medical School, Institute of Pharmacology, Hannover Lower Saxony, Germany

##### **Correspondence:** Bastian Schirmer (schirmer.bastian@mh-hannover.de)

*J Transl Med* 2022, **21(1)**:P36

**Introduction:** The effector protein Exotoxin Y (ExoY) of *Pseudomonas aeruginosa* is a promiscuous nucleotidyl cyclase activated by F-actin. Infected cells thus show significantly elevated concentrations of the 3′,5′-cyclic nucleotides cAMP, cGMP, cCMP and cUMP. Currently, the role of cUMP and cCMP in *P. aeruginosa* infection remains unknown but there are first indications that the pyrimidine cyclic nucleotides serve distinct second messenger roles in the infected host and the bacteria themselves [1]. Although the structure of ExoY has been elucidated, detailed analyses of determinants of substrate selectivity are still missing [2].

**Methods:** Based on the X-ray structure and molecular dynamic simulation, amino acids predicted to be crucial for base-specific enzyme catalysis were altered by site-directed mutagenesis and the resulting mutants were screened for altered substrate selectivity applying highly specific and sensitive HPLC–MS/MS-detection of cyclic nucleotides. Furthermore, we screened for modulators of ExoY activity using a small molecule library including over 700 drugs that are already approved or have been tested in clinical trials. Selected hits of the screening were then subjected to computational docking analyses with AutoDock Vina software [3].

**Results:** All tested site-directed mutations led to an almost complete loss of function of ExoY as assessed by our enzymatic assay. Of the drugs tested, there were multiple modulators of ExoY activity. Some of these drugs were structurally similar and could be grouped according to this similarity. Interestingly, most of the tested substances increase the enzymatic activity of ExoY, although doing so without altering substrate selectivity. Molecular docking studies complemented these mechanistic studies by uncovering potential interaction sites on the enzyme, which can be investigated in future experiments.

**Conclusions:** Although we were not able to identify structural determinants of substrate selectivity, we could show that alteration of amino acids predicted to contact the nucleobases led to a loss of function regarding all substrates. The small molecules we tested did not show to be tools for altering substrate selectivity, but for enhancing overall catalytic activity, which could add to the pharmacological toolbox regarding ExoY research.

**References**

1. Tal, N, Morehouse, BR, Millman, A, Stokar-Avihail, A, Avraham, C, Fedorenko, T, Yirmiya, E, Brandis, A, Mehlman, T, Oppenheimer-Shaanan, Y, Kaszei, AFA, Shao, S, Amitai, G, Kranzusch, PJ, Sorek, R 2021, ‘Cyclic CMP and cyclic UMP mediate bacterial immunity against phages’, *Cell*, 184(23), 5728–5739
2. Khanppnavar, B, Datta, S 2018, ‘Crystal structure and substrate specificity of ExoY, a unique T3SS mediated secreted nucleotidyl cyclase toxin from Pseudomonas aeruginosa’, *Biochim Biophys Acta Gen Subj*.,1862(9), 2090–2103
3. Trott, O, Olson, AJ 2010, ‘AutoDock Vina: improving the speed and accuracy of docking with a new scoring function, efficient optimization and multithreading’, *Journal of Computational Chemistry, 31, 455–461*

## P37 Function of IRAG2 is modulated by NO/cGMP in murine platelets

### Sally Prueschenk, Jens Schlossmann

#### University Regensburg, Pharmacology and Toxicology, Institute of Pharmacy, Regensburg Bavaria, Germany

##### **Correspondence:** Jens Schlossmann (jens.schlossmann@chemie.uni-regensburg.de)

*J Transl Med* 2022, **21(1)**:P37

**Introduction:** IRAG2 (synonym: Jaw1, LRMP) is a protein located in the endoplasmic reticulum and is associated with IP_3_R e.g. in exocrine acinar cells (Behrens et al. 1994; Prüschenk et al. 2021). IRAG2 is a homologue of IRAG1, a substrate protein of cGMP-dependent protein kinase I (PKGI) in platelets. Previously, it was reported that IRAG1 prevents platelet function via NO/cGMP-signalling (Antl et al. 2007). Here, we studied whether IRAG2 is also located in platelets, might be a substrate protein of PKGI and a modulator of platelet function.

**Methods:** Global *Irag2* deficient mice (IRAG2-KO mice) were generated as described before (Chang et al. 2021). Murine platelets were isolated as described previously (Antl et al. 2007). Platelet aggregation was analyzed by optical aggregation analysis as described (Schinner et al. 2011). Co-immunoprecipitation with anti-IRAG2 antibody (Sigma‑Aldrich®) was studied as described (Prüschenk et al. 2021). Proteins were analyzed by SDS-Page and Western Blot. Further primary antibodies for immunoblotting were: anti-IP_3_R1 (Novusbio), anti-IP_3_R2 (Santa Cruz), anti-IP_3_R3 (BD Transduction Laboratories™): anti-IRAG1 (in house production), anti-LRMP (IRAG2) (Sigma‑Aldrich®), anti-β-Galactosidase (Abcam), anti-PKGIβ (in house production), anti Phospho-(Ser/Thr) PKA substrate (Cell Signaling Technology). After secondary antibody treatment (anti‑mouse (Sigma‑Aldrich®), anti‑rabbit (Dianova GmbH) or anti-goat (Dianova GmbH, Hamburg, Germany) detection was carried out by ECL and ChemiDoc™ MP Imaging System (Bio‑Rad Laboratories).

**Results:** IRAG2 protein was detected by immunoblotting with IRAG2 antibodies in wild type (WT) compared to IRAG2-KO-platelets. Co-immunoprecipitation analysis revealed that IRAG2 interacts with IP_3_R1-3, but is not stably associated with PKGI or with IRAG1. Phosphorylation of IRAG2 in murine platelets was detected upon cGMP-stimulation. Interestingly, platelet aggregation induced by thrombin or collagen was reduced in the absence of IRAG2. Pretreatment of WT or IRAG2-KO platelets with sodium nitroprusside (SNP) revealed a further reduction of platelet aggregation in the absence of IRAG2.

**Conclusions:** These results indicate that IRAG2 is a substrate of PKGI in murine platelets. Furthermore, IRAG2 might be involved in the induction of thrombin- or collagen-induced platelet aggregation and this effect is augmented by cGMP-dependent IRAG2 phosphorylation. Therefore, IRAG2 might exert an opposing function compared to IRAG1 in murine platelets.

**Funding:** The work was supported by the Bavarian state. Technical support by Astrid Seefeld and Simon Kerler is highly acknowledged.

**References**

1. AntlM, von Brühl ML, Eiglsperger C, Werner M, Konrad I, Kocher T, Wilm M, Hofmann F, Massberg S, Schlossmann J, 2007, IRAG mediates NO/cGMP-dependent inhibition of platelet aggregation and thrombus formation. *Blood* 15, 109:552–559.
2. Behrens TW, Jagadeesh J, Scherle P, Kearns G, Yewdell J, Staudt LM, 1994, Jaw1, A lymphoid-restricted membrane protein localized to the endoplasmic reticulum. *J. Immunol. 153*, 682–690.
3. Chang CY, Wang J, Zhao Y, Liu J, Yang X, Yue X, Wang H, Zhou F, Inclan-Rico JM, Ponessa J et al., 2021, Tumor suppressor p53 regulates intestinal type 2 immunity. *Nature communications 12*, 3371
4. Schinner E, Salb K, Schlossmann J, 2011, Signaling via IRAG is essential for NO/cGMP-dependent inhibition of platelet activation. *Platelets* 22, 217–227.
5. Prüschenk S, Majer M, SchreiberR, Schlossmann J, 2021,^.^ IRAG2 Interacts with IP_3_-Receptor Types 1, 2, and 3 and Regulates Intracellular Ca^2+^ in Murine Pancreatic Acinar Cells. *Int J Mol Sci.* 14, 22:13409.

## P38 Natriuretic peptides signalling in the heart is affected by the cytohesin inhibitor SecinH3

### Daiana Sedneva-Lugovets, Monica Aasrum, Kurt A. Krobert, Soheil Naderi, Lise Román Moltzau

#### University of Oslo, Department of Pharmacology, Institute of Clinical Medicine, Oslo, Norway

##### **Correspondence:** Daiana Sedneva-Lugovets (daiana.sedneva-lugovets@medisin.uio.no)

*J Transl Med* 2022, **21(1)**:P38

**Introduction:** The cardiac hormones natriuretic peptides (NPs) have a significant role in cardiac function, and are considered as potential candidates for heart failure treatment. NPs increase cGMP levels by activating the two NP receptors NPR-A (ANP and BNP) and NPR-B (CNP). Previously, our group has found that CNP causes a negative inotropic response (NIR) through the activation of protein kinase G (PKG). The aim of our project is to study if effects of natriuretic peptides are regulated by cytohesins, a group of proteins we discovered to be associated with NPRs.

**Methods:** The expression of cytohesins 1–4 was determined in rat and mouse ventricular cardiomyocytes by Western blotting and in rat left ventricle tissue by qPCR. Interaction of cytohesins 1–4 with NPR-A and NPR-B was assessed by co-immunoprecipitation in transfected HEK293 cells. Cyclic GMP levels were measured in rat ventricular cardiomyocytes in the presence of the cytohesin inhibitor SecinH3 using an ELISA cGMP assay. Functional responses to NP stimulation and cytohesin inhibition were investigated as changes in contractility of the isolated rat left ventricular muscle strips.

**Results:** We found that cytohesins 1–4 were expressed in rat and mouse ventricular cardiomyocytes and tissue, and that cytohesin-2 and -4 interacted with both NPR-A and NPR-B, with the strongest association found with cytohesin-4. SecinH3 increased NPR-A-mediated cGMP levels, but decreased NPR-B-mediated cGMP. In line with the decreased cGMP levels, SecinH3 reduced the CNP-induced NIR but enhanced its lusitropic effect.

**Conclusions:** Obtained results provide new evidence of the interaction between NPs and cytohesins and support the hypothesis that cytohesins may act as regulators of the NP system in the heart.

## P39 cGMP modulation as a novel therapeutic target for Alzheimer’s disease

### Burcu Seker^1^, Peter Sandner^2^, Rebecca Sienel^1^, Maximilian Dorok^1^, Nikolaus Plesnila^1,3^

#### ^1^University of Munich Medical Center, Institute for Stroke and Dementia Research, Munich Bavaria, Germany; ^2^Bayer AG, Research & Development, Pharmaceuticals, Wuppertal, Germany; ^3^Munich Cluster of Systems Neurology (Synergy), Munich Bavaria, Germany

##### **Correspondence:** Burcu Seker (burcu.seker@med.uni-muenchen.de)

*J Transl Med* 2022, **21(1)**:P39

**Introduction:** Alzheimer’s Disease (AD), is believed to be a future epidemic in industrialized countries. Since no treatment options are available yet, AD will have an unsurpassed socio-economic impact on developed and developing countries, specifically in societies with an aging population. Therefore, novel therapeutic strategies beyond current concepts are in urgent need. Cyclic guanosine monophosphate (cGMP) is a second messenger, which is primarily produced in the brain upon stimulation of soluble guanylyl cyclase (sGC), regulates many physiological processes in the vasculature as well as in synaptic transmission and plasticity. A potential role of sGC-cGMP signaling in AD is further suggested by reduced levels of cGMP in CSF samples from AD patients and an association of cGMP CSF levels and memory decline. Hence, cGMP may play a role in the pathogenesis of AD, and restoring cGMP levels may have a therapeutic potential in AD. In this study, we investigated whether stimulation of sGC improves outcomes in an AD mouse model which expresses human APP and PSEN1 transgenes with a total five AD-linked mutations (5xFAD).

**Methods:** 5xFAD mice were fed with sGC stimulator (Bay41-2272, 45 ppm/kg p.o) or vehicle for up to one year. In addition, wild-type (WT) littermates were fed with normal pellets used as control mice. Memory function was assessed by the Barnes maze test periodically throughout the disease’s progress. At the end of the experiment plasma and brain were collected and drug levels, cGMP/pathway molecules, Aβ plaque load, neuronal survival, and microglia activation were assessed.

**Results:** Our results show that Bay41-2272 has a decent plasma half-live and crosses the blood–brain barrier in sufficient amounts (cBrain/cPlasma: 0.33) and 5xFAD mice show significantly decreased cerebral cGMP levels suggesting reduced sGC activity in our murine AD model. In addition to that, treatment increased mRNA levels of cGMP/pathway molecules by 30% in comparison to vehicle. Bay41 significantly improved memory functions, decreased Aβ plaque load by 35%, and increased neuronal survival and as well as microglial activation by 22% and 23% respectively.

**Conclusions:** We show that sGC stimulation has beneficial effects on memory, plaque load, and neuronal survival in 5XFAD mice. Our results suggest that activation of microglia underpins how cGMP modulation promotes Aβ clearance and may thus represent a promising therapeutic strategy for the treatment of Alzheimer’s disease.

## P40 Heme group increases smooth muscle relaxation of mice corpus cavernosum induced by stimulation of the NO-cGMP signaling pathway: implication for priapism in sickle cell disease

### Dalila A. Pereira, Fabiano B. Calmasini, Fábio H. Silva

#### Sao Francisco University, Laboratory of Multidisciplinary Research, Bragança Paulista, Brazil

##### **Correspondence:** Fábio H. Silva (fabiohsilva87@gmail.com)

*J Transl Med* 2022, **21(1)**:P40

**Introduction:** Priapism is a urologic emergency and is an important clinical problem for patients with sickle cell disease (SCD). Priapism is defined as a prolonged and painful penile erection that can cause damage to penile tissue and progress to permanent erectile dysfunction. Clinical studies point to a strong and positive correlation between priapism and high levels of intravascular hemolysis in patients with SCD [1–2]. Intravascular hemolysis leads to the release of hemoglobin into the plasma that can consume nitric oxide and be oxidized. During these reactions, the heme group is released from hemoglobin and metabolized by heme oxygenase, generating carbon monoxide (CO). CO can promote smooth muscle relaxation via GCs-cGMP [3]. However, to date, no study has evaluated the role of heme group in the erectile function of mice. We hypothesized that CO production, as a byproduct of heme metabolism, may contribute to priapism through activation of the GCs-cGMP pathway in corpus cavernosum.

**Methods:** C57BL/6 mice (control) aging 3 to 4 month-old were used. Strips of CC were mounted in isolated organ baths, and the relaxing responses to acethylcholine (ACh; endothelium-dependent responses) and sodium nitroprusside (SNP; endothelium-independent responses), as well to electrical-field stimulation (EFS; nitrergic relaxations) were obtained in cavernosal strips precontracted with the α1-adrenergic receptor agonist phenylephrine (10 µM). Contracting responses to phenylephrine, KCl and EFS were also obtained in corpus cavernosum.

**Results:** Pre-incubation of corpus cavernosum with heme (100 µM) reduced (p < 0.05) the maximal response (Emax) induced by phenylephrine and KCl (0.57 ± 0.11 and 0.37 ± 0.02 mN, respectively, n = 6) compared to the control group (0.9 ± 0.08 and 0.47 ± 0.04 mN, respectively, n = 6), as well as by EFS. Pre-incubation of corpus cavernosum with heme (100 µM) increased (p < 0.05) the maximal response (Emax) induced by ACh and SNP (85 ± 5 and 105 ± 4%, respectively, n = 6) compared to the control group (66 ± 2 and 80 ± 4%, respectively, n = 6), as well as by EFS. Pre-incubation of corpus cavernosum with heme-oxygenase inhibitor (1 J, 100 µM) or GCs inhibitor (ODQ, 10 µM) inhibited (p < 0.05) the effects of heme (100 µM) on the contractile (phenylephrine, KCL and EFS) and relaxation (ACh, SNP and EFS) response, without changing in the control group.

**Conclusions:** The heme group reduced the smooth muscle contraction of the mice corpus cavernosum, as well as increased relaxation induced by the NO-cGMP pathway in erectile tissue. Therefore, it is likely that heme group may contribute to priapism in SCD.

**Funding:** This work was supported by São Paulo Research Foundation (Grant Number: 2017/08122-9).

**References**

1. Kato, G. J., McGowan, V., Machado, R. F., Little, J. A., Taylor, J., Morris, C. R., et al. Lactate dehydrogenase as a biomarker of hemolysis-associated nitric oxide resistance, priapism, leg ulceration, pulmonary hypertension, and death in patients with sickle cell disease. *Blood* 107, 2279–2285, 2006.
2. Nolan, V. G., Wyszynski, D. F., Farrer, L. A., and Steinberg, M. H. Hemolysis-associated priapism in sickle cell disease. *Blood* 106, 3264–3267, 2005.
3. Stone, J. R., and Marletta, M. A. Soluble guanylate cyclase from bovine lung: activation with nitric oxide and carbon monoxide and spectral characterization of the ferrous and ferric states. *Biochemistry* 33, 5636–5640, 1994.

## P41 Mechanistic insights into inorganic nitrite-mediated vasodilation of isolated aortic rings under oxidative/hypertensive conditions and S-nitros(yl)ation of proteins in germ-free mice

### Paul Stamm^1,2^, Sanela Kalinovic^1^, Matthias Oelze^1^, Sebastian Steven^1,3^, Alexander Czarnowski^1^, Miroslava Kvandova^1^, Franziska Bayer^3^, Christoph Reinhardt^2,3^, Thomas Münzel^1,2^, Andreas Daiber^1,2^

#### ^1^University Medical Center Mainz, Department of Cardiology, Cardiology I, Mainz Rhineland-Palatinate, Germany; ^2^German Center for Cardiovascular Research (DZHK), Partner Site Rhine-Main, Mainz Rhineland-Palatinate, Germany; ^3^University Medical Center Mainz, Center for Thrombosis and Hemostasis Mainz, Mainz Rhineland-Palatinate, Germany

##### **Correspondence:** Paul Stamm (paul.stamm@unimedizin-mainz.de)

*J Transl Med* 2022, **21(1)**:P41

*Published in: Biomedicines, 2022;10:730.* https://doi.org/10.3390/biomedicines10030730.

**Acknowledgements:** We are indebted to Jörg Schreiner and Alexandra Rosenberger for technical assistance.

## P42 Heterogeneity and therapeutic potential of cGMP signaling in the melanoma tumor microenvironment

### Daniel Stehle, Annemarie Stotz, Jennifer Schulz, Robert Feil

#### University of Tübingen, Interfaculty Institute of Biochemistry, Tübingen, Germany

##### **Correspondence:** Daniel Stehle (daniel.stehle@uni-tuebingen.de)

*J Transl Med* 2022, **21(1)**:P42

**Introduction:** Despite improvements through the development of targeted therapies and immune checkpoint blockade during the last decades, late-stage melanoma patients still suffer from high recurrence and suboptimal response rates. This urges the need for new therapeutic approaches. Several reports have linked the cGMP signaling pathway with melanoma. However, while some studies have indicated adverse effects of cGMP on melanoma [1, 2], others observed presumably beneficial effects of cGMP on tumor inflammation [3] and perfusion [4]. We hypothesized that these discrepancies of cGMP’s effects on melanoma development might by due to the involvement of different cell types that express different cGMP signaling pathways.

**Methods:** Here, we used an ex vivo real-time cGMP imaging approach to assess spatiotemporal heterogeneities in cGMP generation in different cells of melanoma tumors. We assessed cGMP concentration changes induced by the guanylyl cyclase ligands NO, atrial natriuretic peptide (ANP) and C-type natriuretic peptide (CNP) in tumors of transgenic mice expressing the cGMP sensor cGi500 in specific cell types [5]. Moreover, we combined a fluorescent reporter system with in situ immunofluorescence staining to analyze the cellular identity of stromal cells and the expression of cGMP pathway proteins in melanoma tumors.

**Results:** We observed the presence of several distinct cGMP response patterns in melanoma tumors: (1) Melanoma cells generated cGMP in response to CNP. (2) Endothelial cells of tumor blood vessels generated cGMP in response to ANP. (3) Pericytes of tumor blood vessels generated cGMP in response to NO. These NO-induced cGMP signals in the pericytes of tumor blood vessels were potentiated by the NO‑sensitive guanylyl cyclase (NO‑GC) stimulator riociguat.

**Conclusions:** Taken together, we uncovered previously unknown cell type-specific cGMP signaling patterns in the stroma of melanoma tumors. These pathways can be utilized to increase cGMP in the tumor microenvironment without inducing cGMP’s deleterious effects in melanoma cells. Based on the previous work of Kashiwagi et al. [4] we suggest that increasing cGMP selectively in tumor pericytes with NO‑GC stimulators might provide a strategy to normalize the defective tumor vasculature. Future studies will show whether NO‑GC modulators can be used as a co-therapy for anti-cancer treatments.

**Acknowledgements:** We’d like to thank Rohit Jain PhD, Shweta Tikoo Ph.D. and Prof. Dr. Wolfgang Weninger (previously Centenary Institute, Sydney; currently Medical University of Vienna) for providing amelanotic B16F10 cells, and Prof. Dr. Andreas Friebe for providing an antibody against the β_**1**_-subunit of NO‑GC. We also thank the Elisabeth und Franz Knoop-Stiftung and the Deutsche Forschungsgemeinschaft (grant number 335549539/GRK 2381; FOR 2060 projects FE 438/5‑2 and FE 438/6‑2) for financial support.

**References**

1. Dhayade, S, Kaesler, S, Sinnberg, T, Dobrowinski, H, Peters, S, Naumann, U, Liu, H, Hunger, RE, Thunemann, M, Biedermann, T, Schittek, B, Simon, HU, Feil, S, & Feil, R 2016, ‘Sildenafil potentiates a cGMP-dependent pathway to promote melanoma growth’, *Cell Rep,* 14(11), 2599–2610, https://doi.org/10.1016/j.celrep.2016.02.028.
2. Arozarena, I, Sanchez-Laorden, B, Packer, L, Hidalgo-Carcedo, C, Hayward, R, Viros, A, Sahai, E, & Marais, R 2011, ‘Oncogenic BRAF induces melanoma cell invasion by downregulating the cGMP-specific phosphodiesterase PDE5A’, *Cancer Cell,* 19(1), 45–57, https://doi.org/10.1016/j.ccr.2010.10.029.
3. Meyer, C, Sevko, A, Ramacher, M, Bazhin, AV, Falk, CS, Osen, W, Borrello, I, Kato, M, Schadendorf, D, Baniyash, M, & Umansky, V 2011, ‘Chronic inflammation promotes myeloid-derived suppressor cell activation blocking antitumor immunity in transgenic mouse melanoma model’, *Proc Natl Acad Sci USA,* 108(41), 17111–17116, https://doi.org/10.1073/pnas.1108121108.
4. Kashiwagi, S, Izumi, Y, Gohongi, T, Demou, ZN, Xu, L, Huang, PL, Buerk, DG, Munn, LL, Jain, RK, & Fukumura, D 2005, ‘NO mediates mural cell recruitment and vessel morphogenesis in murine melanomas and tissue-engineered blood vessels’, *J Clin Invest,* 115(7), 1816–1827, https://doi.org/10.1172/JCI24015.
5. Thunemann, M, Wen, L, Hillenbrand, M, Vachaviolos, A, Feil, S, Ott, T, Han, X, Fukumura, D, Jain, RK, Russwurm, M, de Wit, C, & Feil, R 2013, ‘Transgenic mice for cGMP imaging’, *Circ Res,* 113(4), 365–371, https://doi.org/10.1161/CIRCRESAHA.113.301063.

## P43 Endothelial C-type natriuretic peptide/guanylyl cyclase-B signalling prevents systolic hypertension, aortic stiffness and atherosclerosis in female mice

### Franziska Werner^1^, Lydia Schröder^1^, Takashi Naruke^1^, Sarah Schäfer^2^, Melanie Rösch^2^, Katharina Völker^1^, Lisa Krebes^1^, Marco Abeßer^1^, Kai Schuh^1^, Hideo A. Baba^3^, Frank Schweda^4^, Alma Zernecke^2^, Michaela Kuhn^1^

#### ^1^University of Würzburg, Institute of Physiology, Würzburg Bavaria, Germany; ^2^University Hospital Würzburg, Institute of Experimental Biomedicine, Würzburg Bavaria, Germany; ^3^University Hospital Essen, University Duisburg-Essen, Institute of Pathology, Essen, Germany; ^4^University of Regensburg, Institute of Physiology, Regensburg Bavaria, Germany

##### **Correspondence:** Franziska Werner (franziska.werner@uni-wuerzburg.de)

*J Transl Med* 2022, **21(1)**:P43

**Introduction:** C-Type Natriuretic Peptide (CNP) participates in the paracrine communication of endothelial cells with neighbours like pericytes and perivascular mast cells (1, 2). Thereby CNP regulates microcirculatory flow and blood pressure and stabilizes the vascular barrier. In addition, its´ cyclic GMP-producing guanylyl cyclase-B (GC-B) receptor is expressed in endothelial cells. To elucidate the role of endothelial CNP/cGMP signalling in vascular homeostasis, we studied mice with endothelial GC-B deletion (KO).

**Methods:** See below.

**Results:** Telemetric and tail cuff recordings revealed that female KO mice have increased systolic blood pressure. Doppler Ultrasound demonstrated enhanced aortic pulse wave velocity, indicating stiffness. Indeed, stainings of aortic sections showed that the total, media and adventitial thickness and the number of elastin breaks were increased, whereas the collagen/elastin ratio was decreased in KO compared to controls. Concomitantly the levels of the endothelial adhesion proteins E-Selectin and VCAM-1 were increased in the former. Notably, such genotype-dependent changes did not occur in male mice.

Aortic stiffness and hypertension are risk factors for atherosclerosis. To delineate a possible role of CNP, we studied its’ mRNA expression in aortic regions. CNP levels were greater in the root and arch as compared to thoracic and abdominal segments. Feeding mice a western type diet (21% fat, 10 weeks) increased CNP expression in the aortic root. To dissect a possible protective endothelial role of CNP in atherosclerosis, we intercrossed the EC GC-B KO and control mice with low density lipoprotein receptor deficient (LDLR^−/−^) mice. Western diet provoked marked atherosclerosis, with accumulation of plaques in the aortic root and arch. Notably the plaque areas and heights in the aortic roots (aldehyde fuchsin stainings) were significantly increased in the double KO females. This was accompanied by enhanced macrophage infiltration (immunostainings with Mac-2) and a higher number and area of necrotic cores, indicating unstable plaques. Remarkably such genotype-dependent changes again were only observed in females.

**Conclusions:** Endothelial CNP/GC-B/cGMP signalling protects against aortic stiffness and atherosclerosis. Our own and published observations (3) indicate sex differences in vascular cGMP signalling, CNP outweighing in females and nitric oxide in males. Notably LCZ696, inhibiting neprilysin-mediated CNP degradation, reduced the risk of hospitalization more in women than in men with HFpEF (4). Our observations of increased protective effects of CNP in female mice provide possible mechanistic hints for this clinical finding.

**Funding:** Supported by the Deutsche Forschungsgemeinschaft (DFG KU 1037/8-1, to MK) and by the Universitätsbund Würzburg (AZ 21-25, to FW).

**References**

1. Špiranec K, Chen W, Werner F, Nikolaev VO, Naruke T, Koch F, Werner A, Eder-Negrin P, Diéguez-Hurtado R, Adams RH, Baba HA, Schmidt H, Schuh K, Skryabin BV, Movahedi K, Schweda F, Kuhn M. Endothelial C-Type Natriuretic Peptide Acts on Pericytes to Regulate Microcirculatory Flow and Blood Pressure. Circulation. 2018;138:494–508.
2. Chen W, Werner F, Illerhaus A, Knopp T, Völker K, Potapenko T, Hofmann U, Frantz S, Baba HA, Rösch M, Zernecke A, Karbach S, Wenzel P, Kuhn M. Stabilization of Perivascular Mast Cells by Endothelial CNP (C-Type Natriuretic Peptide). Arterioscler Thromb Vasc Biol. 2020;40:682–696.
3. Moyes AJ, Khambata RS, Villar I, Bubb KJ, Baliga RS, Lumsden NG, Xiao F, Gane PJ, Rebstock AS, Worthington RJ, Simone MI, Mota F, Rivilla F, Vallejo S, Peiró C, Sánchez Ferrer CF, Djordjevic S, Caulfield MJ, MacAllister RJ, Selwood DL, Ahluwalia A, Hobbs AJ. Endothelial C-type natriuretic peptide maintains vascular homeostasis. J Clin Invest. 2014;124:4039–51.
4. McMurray JJV, et al. Effects of Sacubitril-Valsartan Versus Valsartan in Women Compared With Men With Heart Failure and Preserved Ejection Fraction: Insights From PARAGON-HF. Circulation. 2020;141:338–351.

## P44 Tyrosine 112 in the sGC β_1_ subunit: activity enhancer for BAY60-2770

### Theresa Wittrien^1^, Christin Elgert^1^, Anne Rühle^1^, Peter Sandner^2^, Sönke Behrends^1^

#### ^1^Technical University Braunschweig, Institut of Pharmacology, Toxicology and Clinical Pharmacy, Braunschweig Lower Saxony, Germany; ^2^Bayer AG, Cardiovascular Research, Wuppertal North Rhine-Westphalia, Germany

##### **Correspondence:** Theresa Wittrien (t.wittrien@tu-bs.de)

*J Transl Med* 2022, **21(1)**:P44

**Introduction:** Based on their studies with the mutant α_1_/β_1_Y112A, Rekowski et al. suggested Y112 as important interaction site for cinaciguat-like activators with a long lipophilic tail [1]. In their EM and activity studies, Liu et al. also highlighted the importance of Y112 for the binding of cinaciguat [2]. Recently, we introduced the partial agonist BAY‑543 and proposed that BAY‑543 with its rather short site chain might be unable to reach the activity enhancer Y112, contributing to its weaker activation [3]. To test our hypothesis, we performed activity measurements with the mutant α_1_/β_1_Y112A comparing the activators BAY60-2770 with a long lipophilic tail and BAY‑543 with a short lipophilic tail.

**Methods:** Enzyme overexpression in HEK 293 and Sf-9 cells. Purification from Sf-9 cytosolic fraction. UV/VIS absorbance analyses. Activity measurements with [α-32P]-GTP.

**Results:** Activity measurements with purified α_1_/β_1_ and α_1_/β_1_Y112A revealed a decrease in the efficacy of BAY60-2770 in the mutant, whereas the efficacy of BAY‑543 was not affected by the mutation of Y112. Interestingly, in HEK 293 cells both BAY‑543 and BAY60-2770 showed an increased efficacy in α_1_/β_1_Y112A compared to α_1_/β_1_. The activity increase was more pronounced for BAY‑543, resulting in a higher V_max_ of BAY‑543 than BAY60-2770 in the mutant. This is a reversion of the situation in wildtype, where the efficacy of the partial agonist BAY‑543 was lower than the efficacy of the full agonist BAY60-2770.DEA/NO dose response curves and UV/VIS absorbance analysis showed a lower heme content of α_1_/β_1_Y112A compared to α_1_/β_1_. Furthermore, comparison of dose response curves with BAY60-2770, BAY‑543 and DEA/NO in HEK 293 cells and purified protein indicated a higher sGC heme content in the HEK 293 cell system than in purified enzyme.

**Conclusions:** Activity measurements in purified enzyme supported our hypothesis of Y112 in the β_1_ H-NOX domain to enhance the activation by the full agonist BAY60-2770, but not by the partial agonist BAY‑543. Data from HEK 293 cells seemed to contradict these observations. However, we identified differences in heme content as confounding factor. The mutant α_1_/β_1_Y112A exhibited a lower heme level than α_1_/β_1_. As activators preferably activate heme free sGC, this explains the increased activator influence in α_1_/β_1_Y112A. This increase was not detected in purified protein. Again, differences in heme content serve as explanation: the heme content of α_1_/β_1_ overexpressed in HEK 293 cells appeared multifold higher than that of purified α_1_/β_1_. Accordingly, the heme loss introduced by the mutation of Y112 preponderates in the high heme content cell system HEK 293.

**References**

1. Rekowski, Margarete von Wantoch; Kumar, Vijay; Zhou, Zongmin; Moschner, Johann; Marazioti, Antonia; Bantzi, Marina et al. (2013): Insights into soluble guanylyl cyclase activation derived from improved heme-mimetics. In: *Journal of medicinal chemistry* 56 (21), S. 8948–8952. https://doi.org/10.1021/jm400539d.
2. Liu, Rui; Kang, Yunlu; Chen, Lei (2021): Activation mechanism of human soluble guanylate cyclase by stimulators and activators. In: *Nature communications* 12 (1), S. 5492. https://doi.org/10.1038/s41467-021-25617-0.
3. Rühle, Anne; Elgert, Christin; Hahn, Michael G.; Sandner, Peter; Behrends, Sönke (2020): Tyrosine 135 of the β1 subunit as binding site of BAY-543: Importance of the Y-x-S-x-R motif for binding and activation by sGC activator drugs. In: *European journal of pharmacology* 881, S. 173203. https://doi.org/10.1016/j.ejphar.2020.173203.

## P45 Anti-proliferative effects of cGMP, cAMP, cCMP and cUMP in human carcinoma cell lines

### Sabine Wolter, Sarah Sharifi, Anna M. Postels, Roland Seifert

#### Hannover Medical School, Institute of Pharmacology, Hannover Lower Saxony, Germany

##### **Correspondence:** Sabine Wolter (wolter.sabine@mh-hannover.de)

*J Transl Med* 2022, **21(1)**:P45

**Introduction:** Cancer is the first or second leading cause of death before the age of 70 in many countries. In 2020, there were an estimated 19.3 million new cancer cases and nearly 10.0 million cancer deaths worldwide. Female breast cancer has overtaken lung cancer as the most commonly diagnosed cancer, with an estimated 2.3 million new cases. Ovarian cancer is also a common cancer in women^1^. Previously, we demonstrated the apoptosis-inducing potential of cCMP and cUMP in S49 mouse lymphoma cells^2^ and in a human erythroleukemia cell line^3^.

Therefore, we analysed the anti-proliferative effect of the second messengers cGMP, cAMP, cCMP and cUMP in human breast and ovarian cancer cell lines.

**Methods:** We used the alamarBlue assay to analyse proliferation of the human estrogen receptor (ER) and progesterone receptor (PR)-positive MCF-7, the ER- and PR-negative MDA-MB-231 breast cancer cell line and ovarian cancer cell lines PA-1, SK-OV-3 and SW626. Cells in a volume of 100 µl were plated in a 96-well plate in triplicates. After 24 h, cells were treated with cNMP-AMs for 48 h, and alamarBlue (10 µl per well) was added 4 h before measurement. The second messenger functions of cNMPs were imitated by the membrane-permeable acetoxymethyl ester analogues, which release cNMP after intracellular hydrolysis. Expression of multidrug resistant proteins (MRPs) 4 and 5, some selected PDE isoforms were analysed by qRT-PCR analysis. The transporters were inhibited by probenecid and the PDEs by IBMX to alter anti-proliferative effects of cNMP-AMs.

**Results:** All cNMP-AMs showed a time- and concentration-dependent anti-proliferative effect in MCF-7 and MDA-MB-231 cells. Proliferation of PA-1 cells was reduced after treatment with all cNMP-AMs. cAMP-AM inhibited proliferation of SK-OV-3 and SW626 cells were resistant (Figure 1). MRP4, MRP5 and the PDE isoforms (PDE3a, 3b, 5a, 7a and 9a) are expressed at special levels in the different cell lines. Pre-incubation with probenecid increased the activity of cUMP-AM in MCF-7 cells and of cAMP-AM in MDA-MB-231 cells. It reduced proliferation of SW626 cells after cCMP-AM treatment and, surprisingly, increased proliferation of SK-OV-3 cells. IBMX treatment showed only a small effect in breast cancer cells and no significant effect in ovarian cancer cell lines.

**Conclusions:** cNMP export is important in termination of cNMP activity only in specific cells. Expession of PDE isoforms may be crucial for the reaction after IBMX and cNMP treatment. Therefore, isoform-specific PDE inhibitors, also in combination with probenecid should be used in further experiments. In addition, comparative expression-studies could help to understand the different responses of cell lines to the treatment with cNMP-AMs.
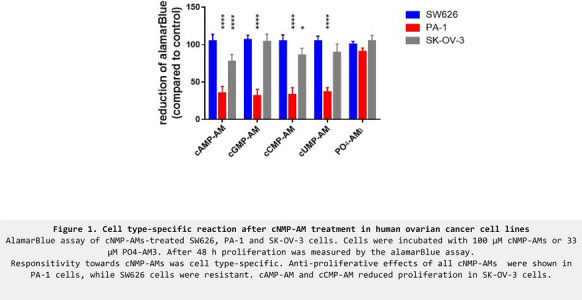


**References**

1. Siegel, RL, Miller, KD, Fuchs, HE, Jemal, A, ‘Cancer Statistics, 2021’, *J Clin*, 7, 209–249, 2021
2. Wolter, S, Kloth, C, Golombek, M, Dittmar F, Försterling, L, Seifert, R, ‘cCMP causes caspase-dependent apoptosis in mouse lymphoma cell lines’, *Biochem Pharmacol,* 98, 119–131, 2015
3. Dittmar, F, Wolter, S, Seifert, R, ‘Regulation of apoptosis by cyclic nucleotides in human erythroleukemia (HEL) cells and human myelogenous leukemia (K-562) cells’, *Biochem Pharmacol*, 111, 13–23, 2016

## P46 A new generation of sGC stimulators: discovery and characterization of BAY-747, member of a novel, long-acting class of sGC stimulators for the use in hypertension

### Frank Wunder^1^, Alexandros Vakalopoulos^1^, Gorden Redlich^1^, Andreas Knorr^1^, Thomas Mondritzki^1^, Damian Brockschnieder^1^, Hanna Tinel^1^, Eva-Maria Becker-Pelster^1^, Ingo V. Hartung^1,2^, Peter Sandner^1^, Johannes-Peter Stasch^1^, Markus Follmann^1^

#### ^1^Bayer AG Pharmaceuticals, Research and Development, Wuppertal, Germany; ^2^Merck KGaA, Merck Healthcare, Darmstadt, Germany

##### **Correspondence:** Frank Wunder (frank.wunder@bayer.com)

*J Transl Med* 2022, **21(1)**:P46

**Introduction:** First-generation sGC stimulators have shown significant clinical benefit in pulmonary arterial hypertension (riociguat), and chronic heart failure (vericiguat). However, given the very broad therapeutic opportunities for sGC stimulators, more tailored molecules for distinct indications with special pharmacokinetics, tissue distribution and physicochemical properties will be required in the future.

**Methods:** Here, we report the ultra-High-Throughput (uHTS)-based discovery of a second-generation of sGC stimulators from the imidazo[1,2-*a*]pyridines lead series. The initial screening hit was intensively optimized by a medicinal chemistry optimization program. The optimized sGC stimulator BAY-747 was broadly characterized in vitro, ex vivo and in vivo in pharmacological model systems relevant for vasodilatation and hypertension. In addition, the pharmacokinetic profile of BAY-747 was determined in different species.

**Results:** Through the broad, intensive and staggered optimization of the initial screening hit, potency, metabolic stability, permeation and solubility could substantially be improved and optimized in parallel. These efforts resulted, finally, in the discovery of the new sGC stimulator BAY-747. With the differentiated and long-acting pharmacodynamic effects, BAY-747 turned out to be optimal for indications and tailored uses where a very low peak/trough ratio is required. Thus, BAY-747 could be an ideal treatment alternative for patients with hypertension, especially those not responding to standard therapy.

**Conclusions:** In summary, we discovered the novel imidazo[1,2-*a*]pyridine BAY-747 as a potent, orally available sGC stimulator. Our initial uHTS screening hit served as the starting point for this research work. The intensive optimization of the initial screening hit led to the identification of BAY-747 with superior pharmacokinetic and pharmacodynamic profiles. In addition, experiments in hypertensive animals suggest that BAY-747 might provide an effective therapy option for malignant and therapy resistant hypertension.

